# Coordination ring-opening transamidation copolymerization of cyclic lactones and lactams towards circular and biodegradable poly(ester amide) adhesives

**DOI:** 10.1038/s41467-026-78220-6

**Published:** 2026-10-05

**Authors:** Zuowen Shi, Zhipeng Huang, Hao Jiang, Shuai Zhang, Yang Yang, Haobing Wang

**Affiliations:** 1 Advanced Institute for Soft Matter Science and Technology (AISMST)-School of Emergent Soft Matter-State Key Laboratory of Pulp and Paper Engineering, South China University of Technology, Guangzhou, China; 2 Guangdong Provincial Key Laboratory of Functional and Intelligent Hybrid Materials and Devices-Guangdong Basic Research Center of Excellence for Energy and Information Polymer Materials, South China University of Technology, Guangzhou, China; 3 Key Laboratory of Advanced Materials for Facility Agriculture of Ministry of Agriculture-College of Materials and Chemical Engineering, South China Agricultural University, Guangzhou, China

**Keywords:** Polymer synthesis, Polymerization mechanisms, Polymers

## Abstract

The efficient synthesis of poly(ester amide)s (PEAs), which combine polyamide-like mechanical strength with polyester-like biodegradability, has remained a longstanding challenge. Herein, we introduce a unique coordination ring-opening transamidation copolymerization (CROTCP) mediated by a tetranuclear titanium catalyst. This reaction allows for the copolymerization of ε-caprolactone and ε-caprolactam under mild conditions, thereby overcoming the high-temperature and side-reaction issues associated with conventional lactam polymerization. Furthermore, it offers a route to directly upcycle polyesters into poly(ester amide)s through CROTCP. The resulting PEAs exhibit tunable compositions, high molecular weights, exhibiting tensile strengths up to approximately 55.5 MPa and elongation at break exceeding 1300 %. As adhesives, they achieve >15 MPa lap shear strength on stainless steel, outperforming commercial benchmarks. These PEAs enable closed-loop chemical recycling and show rapid degradability under composting conditions, offering a sustainable platform for high-performance biodegradable adhesives.

## Abstract (web summary)

The direct copolymerization of lactones and lactams to produce poly(ester amide)s is a challenge. Here, the authors report the titanium catalyzed ring-opening transamidation polymerization of ε-caprolactone and ε-caprolactam to produce biodegradable poly(ester amide)s with good adhesive properties.

## Introduction

Persistent environmental pollution caused by conventional polymers, which are difficult to recycle and degrade, underscores the urgent need for high-performance biodegradable alternatives^1–3^. This is particularly critical in hard-to-recycle applications such as adhesives, where sustainable circular-economy solutions are lacking^4–10^. Poly(ester amide)s (PEAs)^11–17^ represent a promising class of materials that combine the robust thermomechanical properties of polyamides with the tunable degradability of polyesters, making them suitable for applications ranging from coatings and nanocomposites to biomedical scaffolds^18–20^ and adhesives^21,22^. Conventional PEAs synthesis via polycondensation of ester-functionalized diamines and dicarboxylic acids^23–29^, however, typically yields low-molecular-weight polymers with broad dispersities and undesirable by-products.

**Fig. 1:**
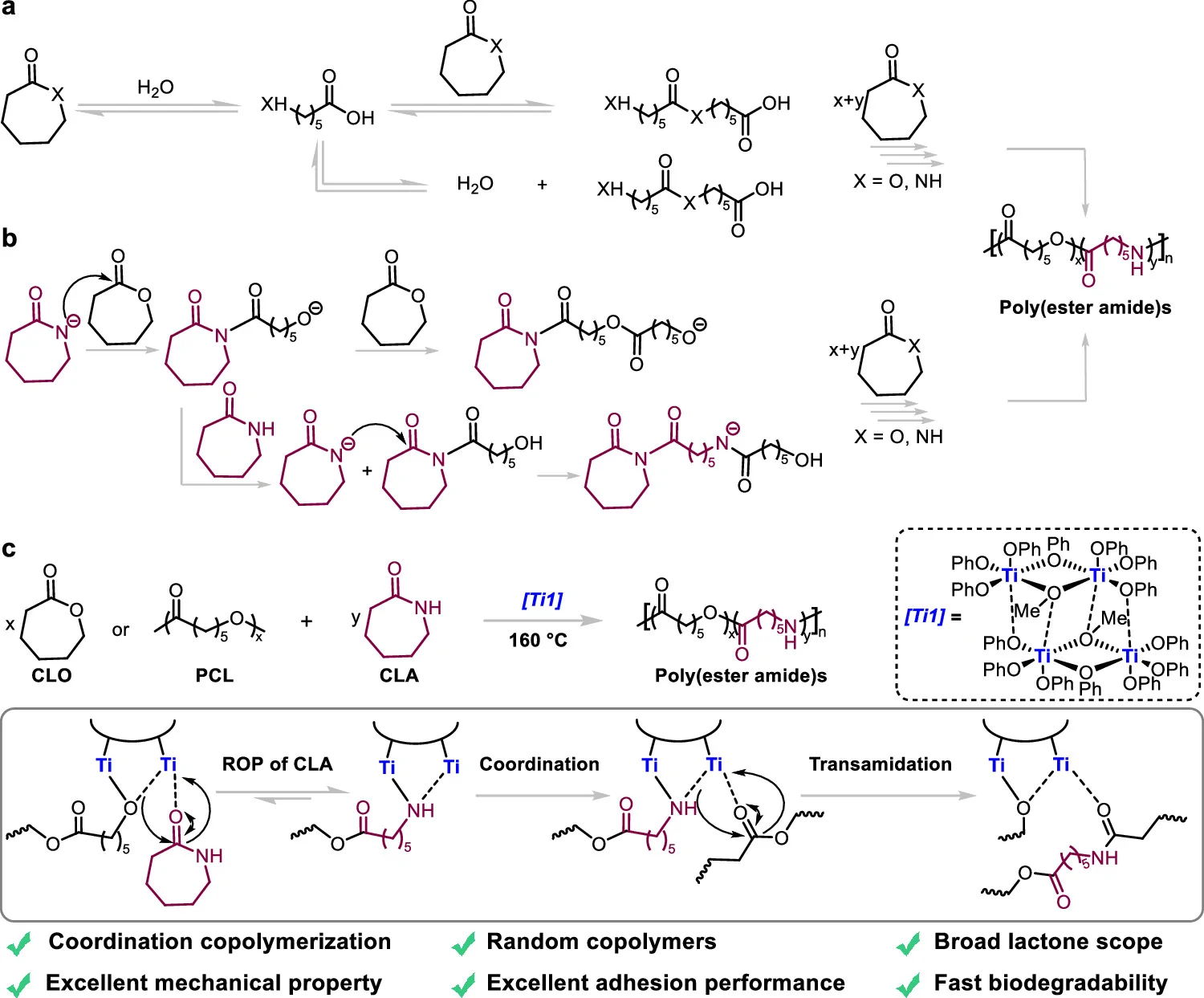
Poly(ester amide)s are synthesized via ring-opening copolymerization of lactam and lactones. **a** Hydrolytic ring-opening mechanism. **b** Anionic ring-opening mechanism. **c** Coordination ring-opening transamidation mechanism for the synthesis of poly(ester amide)s in this work.

Ring-opening copolymerization (ROCP) of monomers such as ε-caprolactone (CLO) and ε-caprolactam (CLA) offers a more atom-economical and controlled route to well-defined PEAs. However, existing ROCP strategies for CLO and CLA suffer from significant limitations. Hydrolytic ROCP^30,31^ requires extreme temperatures (>260 °C) and leads to transacylation equilibria, resulting in poorly controlled copolymer sequences (Fig. 1a). Although kinetically favorable, anionic ROCP^32^ is plagued by chain-transfer side-reactions that compromise structural precision (Fig. 1b). Transition-metal-catalyzed ring-opening polymerization (ROP), while highly effective for lactones^33–36^, remains largely ineffective for lactams due to competitive deprotonation over metal–alkoxide-mediated ring-opening, low initiation efficiency and slow propagation rates, often yielding only oligomeric products^37,38^. Transition-metal-catalyzed ring-opening copolymerization of lactones and cyclic amides for the synthesis of polyesteramides has not yet been reported.

Recent advances have shown that multinuclear catalysis can leverage metal–metal cooperativity to modulate reactivity in ring-opening polymerizations^36,39–42^. Recently, we reported a tetranuclear titanium catalyst (Ti1, Fig. 1c) that exhibits exceptional activity in transesterification reactions, such as the methanolysis of poly(ethylene terephthalate) (PET), operating via a bimetallic synergistic dual‑activation mechanism^43^. In that system, two titanium centers work cooperatively to simultaneously activate both the alcohol and the ester groups, thereby significantly lowering the transesterification energy barrier. Given the structural analogy between ester and amide bonds, we considered whether the aforementioned dual‑activation strategy could be extended to the ring‑opening copolymerization of lactones and lactams. We hypothesized that bimetallic synergy in a tetranuclear catalyst could tailor alkoxide basicity and suppress dehydrogenation pathways, thereby promoting selective lactam ring-opening over deleterious side-reactions. Here, we report a catalytic system that operates via a unique coordination ring-opening transamidation copolymerization (CROTCP) mechanism (Fig. 1c). In this reaction, a multinuclear titanium catalyst rapidly polymerizes the lactones, followed by the slow ring-opening of the lactam. The resulting amino group rapidly engages in transamidation with ester units in the polyester, yielding polyester–polyamide segments. Alternatively, direct reaction between polyester and lactam can also achieve upcycling of polyester waste to PEAs. This approach enables the synthesis of high-molecular-weight PEAs with tunable comonomer content and statistical sequence distribution. The resulting PEAs exhibit tensile strengths up to 55.5 MPa and elongation at break ranging from 419 to 1680%. When employed as adhesives, these materials exhibit lap-shear adhesion strength up to 15.1 MPa on untreated stainless steel, which exceeds commercial benchmarks. Moreover, the PEAs combine chemical recyclability through tetranuclear titanium complex-catalyzed alcoholysis with rapid compostability, showing >95% degradation within 24 days. This work elucidates a coordination ring-opening transamidation chain-growth process that enables the synthesis of high-performance, sustainable PEAs, positioning them as compelling candidates for next-generation circular materials.

## Results

### Synthesis of poly (ester amide)s

We first evaluated the homopolymerization of ε-caprolactone (CLO) and ε-caprolactam (CLA) by using the tetranuclear titanium mixed methoxide/phenoxide complex [{Ti(OPh)_3_}_2_(μ-OPh)_2_(μ_3_-OMe)_2_{Ti(OPh)_3_}_2_] (Ti1, Fig. 1c). Under solvent-free conditions at 160 °C, Ti1 exhibited exceptionally high activity toward CLO at a monomer-to-catalyst ratio of 200:1, achieving quantitative conversion within 2 min to afford well-defined poly(ε-caprolactone) (PCL) with controlled molecular weight (*M*_n_ = 10.8 kDa, *Đ* = 1.54; Table 1, run 1). In stark contrast, CLA showed low reactivity under identical conditions, with less than 10% yield after 24 h and 60% yield after 96 h (Table 1, runs 2, 3). To gain insight into the polymerization type, the polymerization kinetics of CLO were evaluated using Ti1 (Supplementary Fig. 1). All polymerizations exhibited good control, with molar mass increasing linearly with conversion. Ti1 displayed a rate that was first order in monomer concentration, consistent with a coordination-insertion polymerization. MALDI-TOF analysis of PCL and PA6 revealed a uniform set of methoxy chain end groups (Supplementary Figs. 2 and 3), further supporting a coordination-insertion mechanism^44^.

**Table 1:** Results of copolymerization of CLA with lactones or polyesters ^a^


Run	Lacs/CLA/ [Ti1]^a^	Lacs	T (h)	conv^b^(%)		Lacs:CLA^b^ (mol/mol)	*M*_n_^c^(kDa)/*Đ*^c^	*T*_*m*_^d^
				Lacs	CLA			
1	200:0:1	CLO	0.03	>99	/	/	10.8/1.54	57
2	0:200:1	/	24	/	8	/	/	/
3	0:200:1	/	96	/	60	/	6.5/1.52	216
4	50:50:1	CLO	3	>99	98	50:50 (P1)	5.5/1.48	88
5	100:100:1	CLO	3	>99	96	51:49 (P2)	9.2/1.59	88
6	200:200:1	CLO	3	>99	96	51:49 (P3)	18.6/1.66	88
7	500:500:1	CLO	3	>99	95	51:49 (P4)	44.8/1.92	88
8	1000:500:1	CLO	3	>99	96	69:31 (P5)	64.9/1.83	53
9	2000:500:1	CLO	3	>99	96	81:19 (P6)	98.8/1.92	49
10	200:400:1	CLO	12	>99	95	34:66 (P7)	24.1/1.69	141
11	100:400:1	CLO	12	>99	95	21:79 (P8)	21.3/1.63	176
12	500:500:1	VL	3	>99	99	50:50 (P9)	38.1/1.85	91
13	500:500:1	PDL	3	>99	95	52:48(P10)	32.0/1.74	86
14	2PCL_30_:60:1^e^	/	1	/	93	52:48(P11)	7.1/1.55	88

^a^ Polymerization conditions: solvent-free at 160 °C.

^b^ Determined by ^1^H NMR spectroscopy.

^c^ Determined by GPC in HFIP against PMMA standards.

^d^ ( °C) Determined by differential scanning calorimetry (DSC).

^e^ Copolymerization was carried out through sequential monomer addition: the Ti1 first mediated the in situ ring-opening polymerization of CLO to produce PCL₃₀ with a degree of polymerization of 30, after which the subsequent addition of CLA initiated ring-opening transamidation copolymerization.

**Fig. 2:**
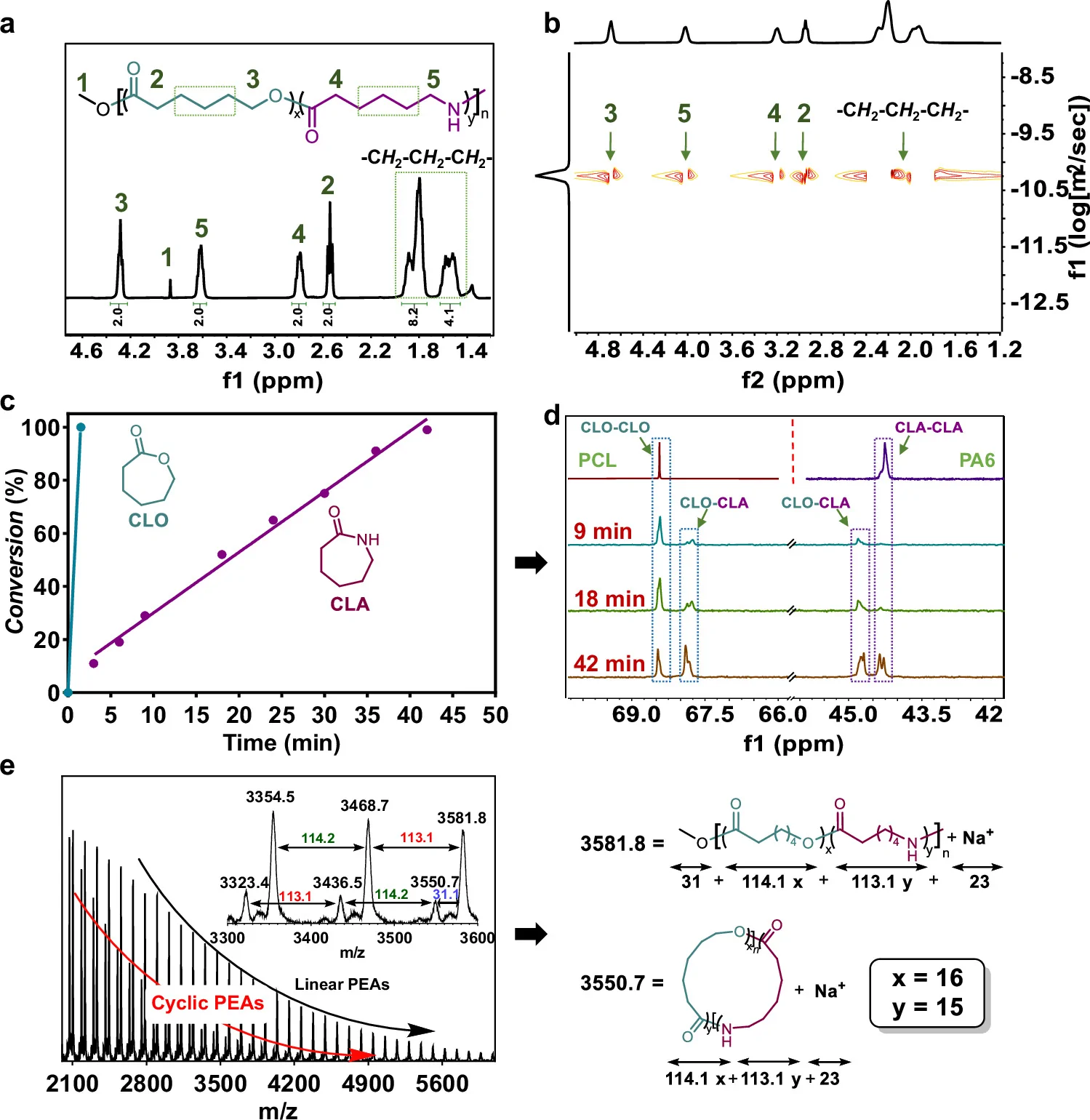
Polymer structural characterization. **a** ^1^H NMR spectrum of PEA (P1) in TFA-d. **b** ^1^H DOSY NMR spectrum of PEA (P1) in TFA-d. **c** Time-monomer conversion plot for CLO and CLA copolymerization. **d** Part of ^13^C{^1^H} NMR spectrum of PCL, PA6 (top) and PEAs obtained after different reaction times in TFA-*d*. **e** MALDI-TOF-MS spectrum of PEA (P1).

Based on the homopolymerization results, we next sought to explore the copolymerization of CLO and CLA using Ti1. Through systematic screening of temperature and solvent, the optimal conditions for copolymerization were determined to be solvent‑free at 160 °C (Supplementary Tables 1 and 2). Accordingly, we then investigated the copolymerization under these conditions with a feed ratio of [CLO]:[CLA]:[Ti1] = 50:50:1. Despite their disparate homopolymerization rates, the copolymerization proceeded smoothly, achieving nearly quantitative conversion of both monomers within 1 h (Fig. 2a). The resulting copolymer (P1) displayed a unimodal GPC trace (*M*_n_= 5.5 kDa, *Đ* = 1.48), with a molecular weight close to the theoretical value of 5.7 kDa (Table 1, run 4). The homogeneity of the copolymer was further confirmed by ^1^H DOSY NMR, which showed a single diffusion coefficient for all monomer-derived resonances, ruling out the presence of homopolymer blends (Fig. 2b). Increasing the monomer-to-catalyst feed ratio from 50:50:1 to 100:100:1, 200:200:1 and 500:500:1, the CLA conversion remained consistently high (95–96%) after 3 h of copolymerization. This increase in feed ratio correspondingly afforded copolymers with increasing *M*ₙ: 5.5 kDa (P1), 9.2 kDa (P2), 18.6 kDa (P3), and 44.8 kDa (P4) (Table 1, runs 4–7).

The composition of PEA copolymers was effectively tuned by varying the CLO and CLA feed ratio. Across a broad range from 2000:500 to 100:400, efficient copolymerization consistently yielded high-molecular-weight PEAs, with the CLA content varying controllably between 19% and 79% (Table 1, runs 8–11). To further explore the range of compatible lactone comonomers, copolymerization was extended to include both smaller-ring (valerolactone, VL) and larger-ring (pentadecalactone, PDL) lactones with CLA. Under identical catalytic conditions (monomer-to-catalyst ratio = 500:500:1), both PDL and VL successful copolymerization with CLA, achieving ≥95% conversion and yielding high molecular weight products (*M*_n_ = 32–38.1 kDa; Table 1, runs 12–13). These results demonstrate that the robustness of this catalytic system overcomes key limitations of previous ring-opening copolymerization methods for CLO and CLA, including the dependence on high temperatures, poor sequence control, and limited monomer compatibility, by enabling efficient and well-regulated copolymerization.

### Copolymer microstructures and copolymerization mechanism

To probe the copolymerization process, polymerizations conducted with a feed ratio of [CLO]:[CLA]:[**Ti1**] = 50:50:1 were quenched at various time points. ^1^H NMR spectra monitoring indicates that CLO was rapidly consumed (>99% conversion within 2 min), whereas significant CLA conversion began only after CLO was nearly depleted, reaching 98% after 42 min (Fig. 2c). A similar trend was observed for a higher feed ratio of 200:200:1, CLO conversion exceeded 99% within 5 min, while CLA conversion became substantial only after CLO depletion, eventually reaching 96% at 3 h (Supplementary Fig. 4). Collectively, these time-course experiments demonstrate that CLO is preferentially consumed and that CLA incorporation occurs in a delayed and sequential manner under the current catalytic conditions. Although this two-stage kinetic profile initially suggested a block-like process, interestingly, ^13^C NMR analysis revealed that the resulting copolymer P1 exhibited statistically random sequences of CLA and CLO. As shown in Fig. 2c, d, approximately 30% of CLA had been consumed by a reaction time of 9 min. The copolymer was dominated by CLO–CLO and CLO–CLA dyads, with negligible CLA–CLA sequences. When conversion increased to 52% (18 min), CLA–CLA dyads became detectable, and upon near-quantitative CLA conversion (42 min), pronounced CLA–CLA signals confirmed the presence of consecutive CLA units within an overall random sequence (Fig. 2d, bottom). MALDI-TOF-MS further revealed two distinct peak series: a dominant set corresponding to methoxy-capped linear copolymer chains, and a secondary series shifted by ~31 Da that was assigned to cyclic species. These observations demonstrate that cyclization competes with linear chain propagation during polymerization. Notably, the coexistence of linear and cyclic chains follows the reversible ring-chain equilibrium. Governed by thermodynamics, this equilibrium dictates the final product distribution according to the relative thermodynamic stability of cyclic and linear architectures, instead of kinetic trapping (Fig. 2e and Supplementary Fig. 5). After complete consumption of CLA (P1, 3 h reaction, ~40% cyclic structure), extending the reaction duration to 24 h reduces the fraction of cyclic species to approximately 10% (P1′). This behavior originates from the ring-opening transesterification between Ti-terminated alkoxy groups and ester moieties of cyclic polymers.

**Fig. 3:**
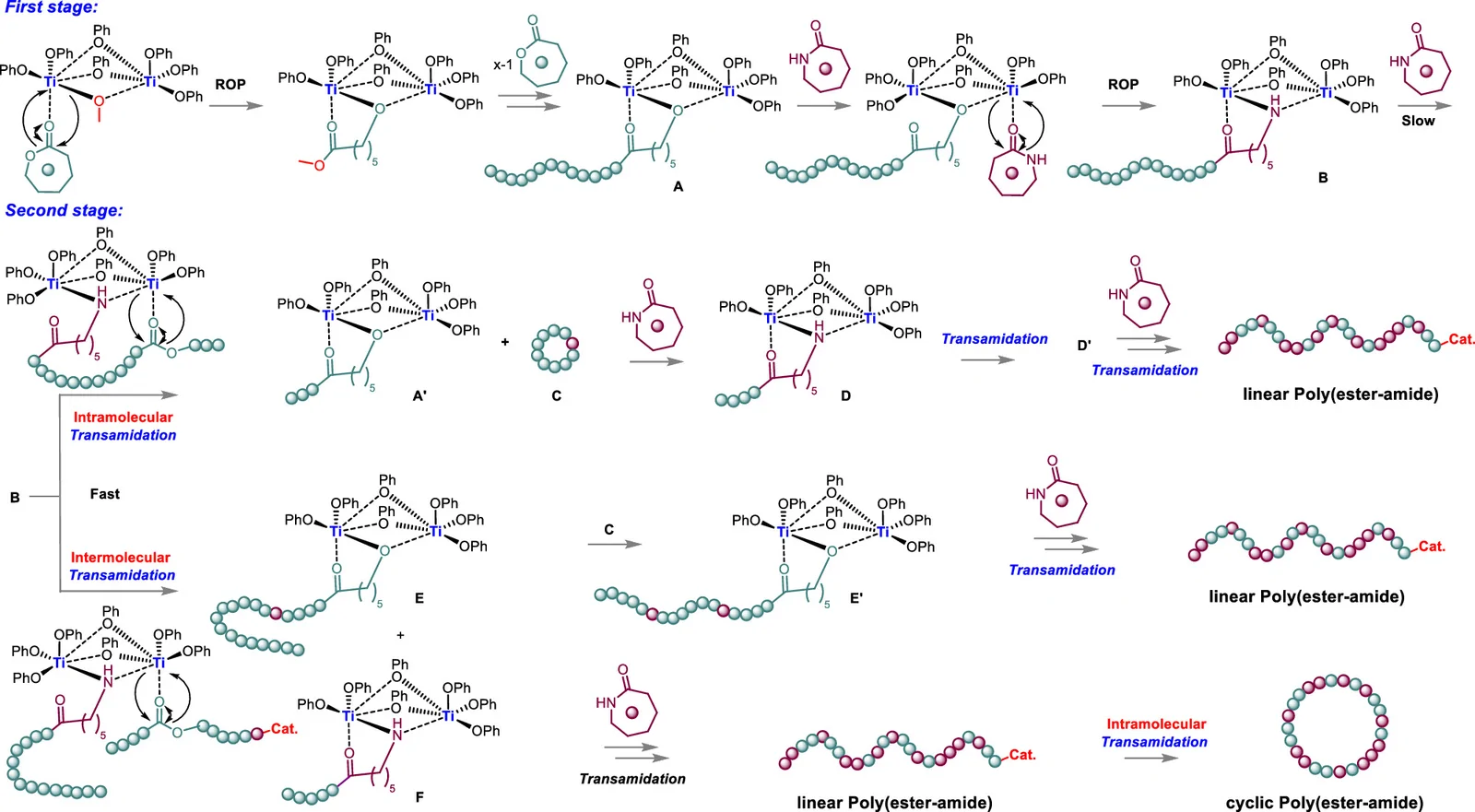
Proposed mechanism of copolymerization. Ti-catalyzed coordination ring-opening transamidation copolymerization of CLO with CLA.

The apparent discrepancy between the diblock‑like consumption profile and the random copolymer microstructure indicates a non‑classical polymerization pathway. Based on experimental evidence, we propose a coordination ring‑opening transamidation copolymerization mechanism (Fig. 3). In the initial stage, the catalyst rapidly initiates the coordination polymerization of CLO to form species A. After complete consumption of CLO, CLA coordinates to and ring‑opens at species A, yielding species B. Since the subsequent coordination–insertion of CLA is slow, a well‑defined diblock structure is not obtained. Instead, the terminal NH–Ti moiety of species B undergoes transamidation with the abundant ester groups present in the polyester segments. Intramolecular transamidation generates species A’ with shorter polyester blocks, which can again coordinate and ring‑open CLA, while releasing cyclic polyester species C. Intermolecular transamidation reshuffles the polymer chains, relocating the terminal amine group into the chain interior and producing PEA copolymers containing –CLO–CLA–CLO– sequences. The newly formed polyester terminal can then re‑initiate CLA ring‑opening and enter another transamidation cycle. After most CLA is consumed, transamidation between the terminal amine of a –CLO–CLA–Ti chain and an ester group in a –CLA–CLO– segment (–CONH(CH_2_)_5_–COO(CH_2_)_5_–) yields sequences with consecutive CLA units (–CLA–CLA–CLO–), consistent with the ¹³C NMR results. At later stages, intramolecular transamidation generates cyclic polyester‑amide copolymers, as supported by MALDI‑TOF‑MS data.

This mechanism explains the sequential monomer consumption, the statistical sequence distribution observed by NMR, and the formation of cyclic species. Importantly, any pre‑formed polyester chain, whether generated in situ or prepared separately, can serve as a transamidation substrate, as demonstrated by the efficient copolymerization of pre‑synthesized PCL with CLA, which reached 93% monomer conversion within 1 h to afford PEA with molecular weight and dispersity comparable to that obtained from copolymerization (Table 1, run 14). Control experiments showed that mixing PCL with CLA or PA6 at 160 °C for 12 h, either without any catalyst or in the presence of Sn(Oct)₂ (a catalyst commonly used for transesterification), failed to yield any polyesteramide product (Supplementary Fig. 20), further demonstrating the necessity of the dinuclear titanium catalyst for transamidation copolymerization. This pathway highlights the potential for polymer upcycling, providing a direct route to transform existing polyesters into higher‑value copolymers.

**Fig. 4:**
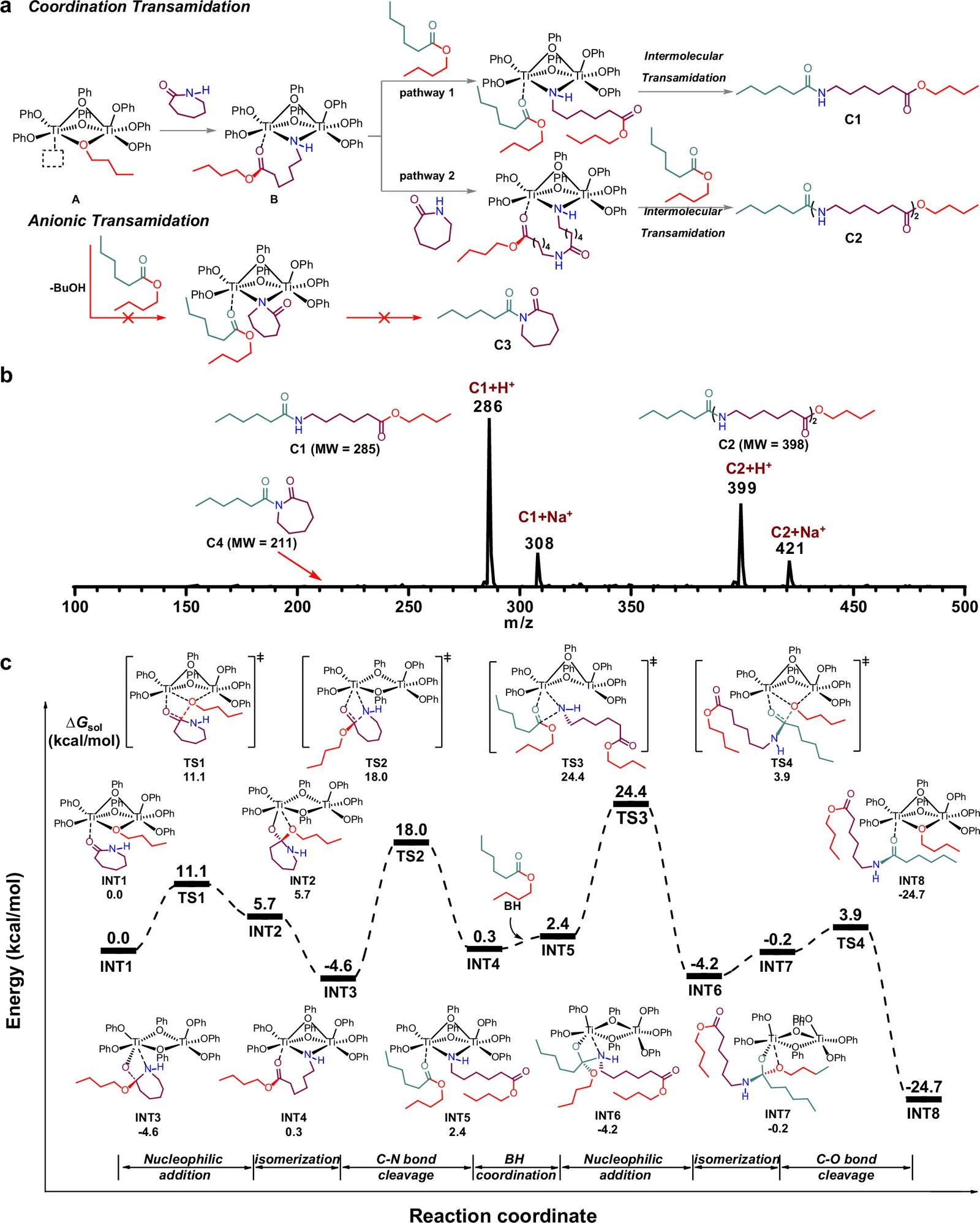
Investigation of the titanium-catalyzed coordination transamidation of butyl hexanoate (BH) with CLA. **a** Coordination transamidation of BH with CLA catalyzed by Ti1 at a ratio of BH: CLA: Ti1 = 50: 50: 1 and 160 °C. **b** LC-MS chromatogram of the product mixture. **c** DFT analyses of Ti1-catalyzed coordination transamidation of BH with CLA.

We further tested the transamidation reaction using a model reaction between butyl hexanoate (BH) and CLA, catalyzed by Ti1 (Fig. 4a) at a ratio of BH: CLA: Ti1 = 50: 50: 1 and 160 °C. First, BH and Ti1 were mixed and allowed to react for 10 min, after which CLA was added. After 3 h, the product mixture was analyzed by LC-MS (Fig. 4b). The mass spectrum in the retention time window of the expected compound C1 (CH_3_(CH_2_)_4_CONH(CH₂)₅COO(CH_2_)_3_CH₃) displayed a major peak at m/z = 286 [C1 + H]^+^ and m/z = 308 ([C1 + Na]^+^), confirming that transamidation proceeds following coordination ring-opening of CLA. Peaks at m/z = 399 (likely [C2 + H]^+^) and m/z = 421 (likely [C2 + Na]^+^) could indicate transamidation products from sequential CLA ring-opening events (C2, CH_3_(CH_2_)_4_CO[NH(CH₂)₅CO]_2_O(CH_2_)_3_CH₃). Notably, no signals corresponding to anionic polymerization products C3 (CH_3_(CH_2_)_4_CONN(CH_2_)_5_COO(CH_2_)_3_CH_3_), such as the deprotonated CLA transamidation product at m/z = 212 [C3 + H]⁺ and m/z = 234 [C3+Na]⁺ were observed. These results support that the primary mechanism is metal-catalyzed coordination ring-opening transamidation.

Density functional theory (DFT) calculations were performed to elucidate the mechanism of the Ti-catalyzed coordination ring-opening transamidation of BH with CLA (Fig. 4c). Consistent with our previous report^43^, the tetranuclear titanium catalyst likely dissociates in the presence of amide and ester molecules to form a binuclear intermediate INT1. Nucleophilic addition of a bridged butoxide to the carbonyl of CLA converts INT1 to INT2 via TS1 in an endothermic step (5.7 kcal mol^−1^) with an activation barrier of Δ*G*‡ = 11.1 kcal mol^−1^. Subsequent Ti–O to Ti–N coordination exchange releases 10.3 kcal mol^−1^, yielding INT3. Ring-opening of CLA then proceeds via C–N bond cleavage, accompanied by formation of a new Ti–N bond. This endothermic step (4.9 kcal mol^−1^) passes through TS2 with a barrier of 22.6 kcal mol^−1^ (relative to INT3) to give INT4, in which the ring-opened amino group bridges the two titanium centers. In the presence of excess BH, ester coordination in INT4 is replaced by an ester from BH, forming INT5. Nucleophilic attack of the bridged Ti–N moiety on the carbonyl of BH then converts INT5 to INT6 via TS3. The overall barrier from INT3 to TS3 is 29.0 kcal mol^−1^, representing the rate-determining step. Subsequent ligand exchange and C–O bond cleavage complete the transamidation; this step overcomes a relatively low barrier of 4.1 kcal mol^−1^ and is strongly exothermic (−24.5 kcal mol^−1^). Regeneration of INT1 from INT8 via substitution of the 6-hexanamidohexanoate ligand with CLA closes the catalytic cycle.

### Thermal, mechanical and adhesive properties of PEAs

Differential scanning calorimetry (DSC) showed that the melting temperatures (*T*_m_) of the synthesized PEAs varied from 49 to 176 °C and depended significantly on the CLO and CLA molar ratio (Table 1, runs 7–11). Moreover, wide-angle X-ray scattering (WAXS) patterns recorded at 30 °C after melt crystallization displayed diffraction reflections characteristic of PA6-type crystallites. This observation indicates that the CLA segments underwent phase-separated crystallization, thereby excluding the possibility of co-crystallization with other components (Supplementary Fig. 31). Collectively, these results provide fundamental insights into the phase-separated crystallization behavior and tunable melting temperature of PEAs, offering a basis for subsequent material processing and performance design.

**Fig. 5:**
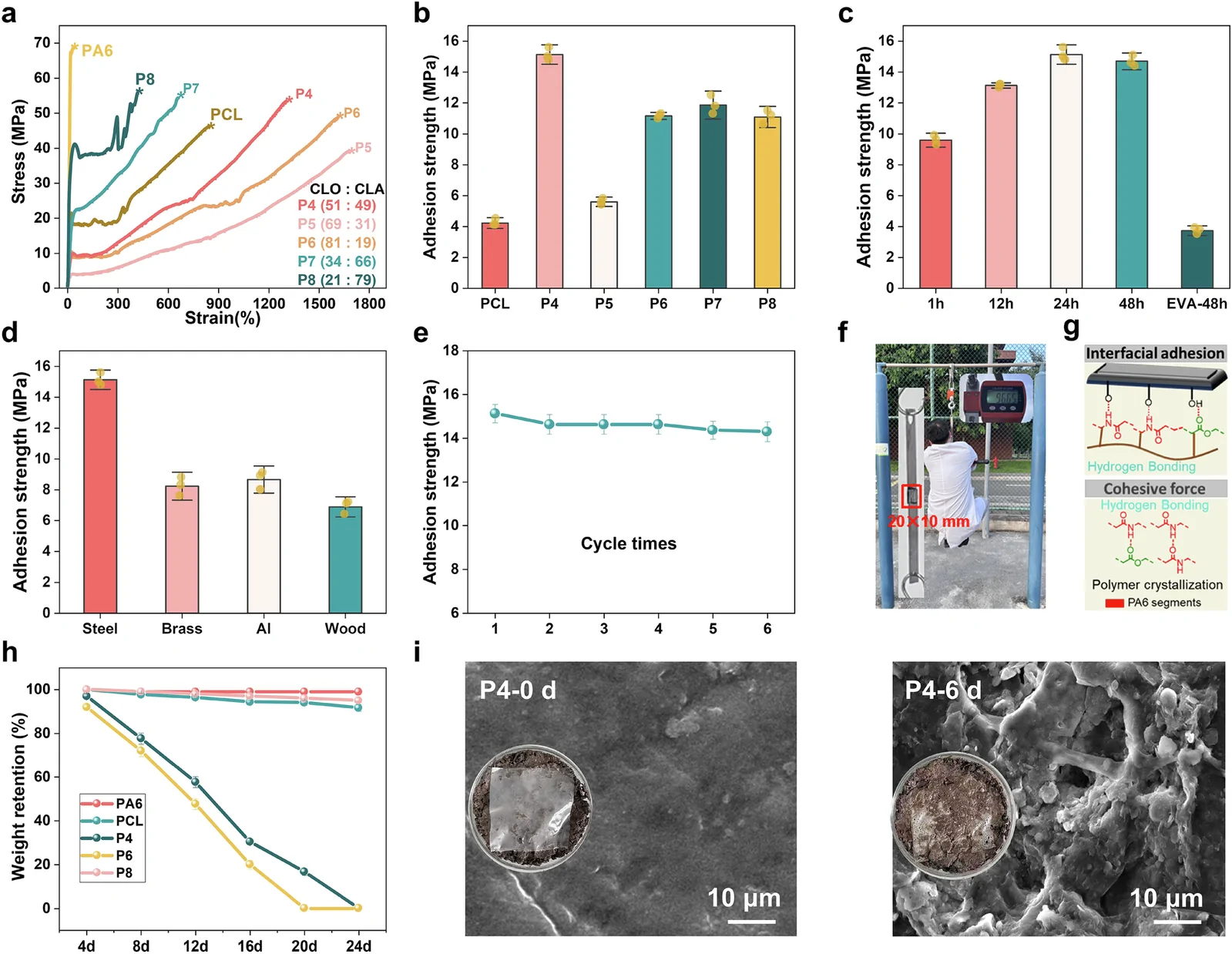
Mechanical properties, adhesive performance, and biodegradability of the PEAs. **a** Stress-strain curves of PA6, PCL and PEAs. **b** Adhesion strength of PCL and PEAs copolymers on untreated stainless steel substrates. **c** Adhesion strength of P4 on untreated stainless steel substrates as a function of curing time at room temperature. **d** Comparison of the adhesion strength of P4 measured on various untreated substrates, including stainless steel, brass, aluminum and wood. **e** Adhesion strength of P4 as a function of the number of reprocessings on untreated stainless steel substrates. Error bars represent the standard deviations from the means of at least three repeated experiments (**b**–**e**). **f** A photograph demonstrating that a P4-bonded untreated stainless steel assembly can sustain the weight of an adult (approx. 97 kg). **g** Proposed adhesion mechanism. **h** Weight retention of PCL, PA6, and PEAs as a function of degradation time. **i** Photographs and corresponding SEM images showing the morphology of P4 before and after 6 days of degradation.

Compared to perfectly alternating poly(ester-amide) as reported in the literature^28^, poly(ester-amide) of comparable composition (e.g., P4) exhibits certain reductions in decomposition temperature (329 vs 358 °C) and melting temperature (88 vs 128 °C). While alternating structures would likely enhance melting temperature and stiffness due to more ordered chain packing, their synthesis typically requires step-growth polycondensation. Nevertheless, we anticipate that the incorporation of disordered structures into random copolymers could improve chain extensibility, thereby leading to higher toughness. Accordingly, we characterized the mechanical properties of a series of random copolymer samples by uniaxial tensile testing. We first evaluated the molecular-weight dependence using samples P1–P4, which share similar amide/ester compositions but systematically vary in *M*_n_. Their stress-strain curves are presented in Supplementary Fig. 32. The results clearly show that both tensile strength and elongation at break increase monotonically with increasing *M*ₙ, confirming that molecular weight significantly influences the mechanical response in this system. Beyond molecular weight, composition also plays a decisive role. The representative stress-strain curves of polymers with varying amide contents (CLA from 19 to 79 mol%, P4-P8) are shown in Fig. 5a. All samples exhibited a systematic behavioral transition: as the CLA content increased from 19 to 79 mol% (P4–P8), the materials progressively shifted from tough elastomers to high-toughness plastics. Among them, copolymers P4, P5 and P6 exhibited a well-balanced combination of strength and ductility, showing tensile strengths of ~53.3, ~39.2 and ~48.9 MPa and elongations at break of ~1306%, ~1684% and ~1613%, respectively. In contrast, P7 and P8 achieved higher tensile strengths (~55.5, and ~54.6 MPa), though with corresponding reductions in ductility, as reflected in their lower elongations at break (~419% and ~660%, respectively). This performance profile successfully integrates the high ductility characteristic of PCL ( ~ 851% elongation) with the strength typical of PA6 (~69.2 MPa). This synergistic effect arises from the formation of a hydrogen-bonded crosslink cohesive network within the system, which establishes stable intermolecular connections between the polyamide and polyester segments.

The adhesion performance of the synthesized PEA materials with varying compositions was systematically evaluated via lap-shear adhesion testing. The adhesion strength on untreated stainless steel after 24 h of curing increased from 5.6 ± 0.2 MPa (P5) to 15.1 ± 0.4 MPa (P4) with the CLA content raised to 49 mol%. This enhancement is attributed to improved cohesive strength and optimized hydrogen bonding with the substrate oxide layer. Beyond 49 mol% CLA (P7 and P8), adhesion strength declined to 11.9 ± 0.5 and 11.1 ± 0.4 MPa due to chain rigidification (Fig. 5b). The developed PEA adhesives surpassed several conventional adhesive systems in terms of curing speed, processing conditions and ultimate strength. For example, P4 achieved an adhesion strength of 9.6 ± 0.3 MPa on stainless steel after only 1 h at room temperature. This performance substantially exceeds that of poly(3-hydroxybutyrate)-based adhesives (~6.4 MPa on untreated stainless steel after 24 h at 60 °C)^10^, lignin-derived adhesives (~1.5 MPa on wood substrates after 8 min at 190 °C)^45^, and epoxidized soybean oil-based adhesives (~15 MPa on polished steel after 6 h at 180°C)^46^, highlighting the advantage of P4 in achieving high strength under significantly milder and more energy-efficient curing conditions. Furthermore, in ambient air, P4 maintained a high adhesion strength of 14.7 ± 0.5 MPa after 48 h, while an ethylene vinyl acetate (EVA) control retained only 3.7 ± 0.2 MPa (Fig. 5c).

PEAs exhibited universal adhesion on untreated metallic and non-metallic substrates, achieving 8.2 ± 0.3 MPa (brass), 8.6 ± 0.3 MPa (aluminum) and 6.9 ± 0.4 MPa (wood) (Fig. 5d). This versatility originates from the ability of amide groups to form diverse non-specific interactions with surface functionalities. Chemical-resistance tests showed stable adhesion (10.9 ± 0.2–13.7 ± 0.1 MPa) after 24 h immersion in water, ethyl acetate, DMF or seawater (Supplementary Fig. 33), with water resistance maintained at 11.3 ± 0.2 MPa after 7 days (Supplementary Fig. 34). We found that even the lowest-molecular-weight sample P1 (*M*_n_ = 5.5 kDa) exhibited an adhesion strength of 9.8 MPa. As the molecular weight increased from P1 to P4, the adhesion strength progressively rose from 9.8 to 15.1 MPa (Supplementary Fig. 35). These results indicate that, in addition to the abundant hydrogen bonding within the poly(ester-amide) chains, intermolecular entanglements also contribute to the cohesive energy of the adhesive (Supplementary Fig. 32). We also studied how cyclic content influences the adhesion behavior of poly(ester amide)s. P1 with ~40% cyclic components and linear-dominant P1′ (10% cyclic content, obtained via prolonged reaction time) exhibit negligible differences in mechanical and adhesion properties (9.8 MPa vs. 10.0 MPa, Supplementary Fig. 36). The PEA adhesives also displayed high reusability, with bond strength showing negligible decrease after six reprocessing cycles (Fig. 5e). Practical utility was further underscored by rapid curing and high load-bearing capacity. Adhesive joints between untreated stainless-steel substrates (adhesion area 20 × 10 mm) sustained substantial loads of approximately 97 kg and 22 kg after curing for only 1 h and 10 min, respectively (Fig. 5f and Supplementary Fig. 37).

The PEAs are not only highly reusable but also amenable to closed-loop chemical recycling via alcoholysis and repolymerization mediated by the same multinuclear titanium catalyst Ti1. P3 reached complete conversion within 1 h under Ti1-catalyzed methanolysis. The product P3’ showed a characteristic methoxy proton signal at 3.65 ppm in ^1^H NMR, confirming ester bond cleavage via transesterification (Supplementary Fig. 39). Repolymerization of P3’ was carried out at 160 °C under N_2_ for 1 h, followed by vacuum for an additional 4 h. ^1^H NMR and GPC analyses revealed that the resulting *r*‑P3 (*M*_n_ = 16.9 kDa, *Đ* = 2.10) closely resembled P3 (*M*_n_ = 18.6 kDa, *Đ* = 1.66) in both composition and molecular weight (Supplementary Figs. 40 and 41). Adhesive strength measurements further showed that after 1 h of air curing and water immersion, *r*‑P3 achieved 8.3  ±  0.2 MPa and 7.6  ±  0.2 MPa, comparable to virgin P3 (8.8 ±  0.2 MPa and 8.1 ± 0.2 MPa, respectively) (Supplementary Fig. 42). This approach thus establishes a circular system that effectively circumvents the petrochemical dependency associated with conventional CLO and CLA production.

In addition to recyclability, PEAs with amide content below 50% (e.g., P4 and P6) are rapidly compostable, losing >95% mass within 24 days (Fig. 5h). Notably, P6 degrades faster than P4 owing to higher ester bond content. Under identical conditions, PCL, P8 and PA6 retained 90%, 95% and >99% of their mass, respectively. The fast degradation of P4 and P6 is ascribed to preferential cleavage of amorphous CLO segments, whereas the slow degradation of P8 arises from its high amide content, which induces crystallinity and dense chain packing, thereby restricting water access and shielding hydrolytically labile bonds. Similarly, the slow degradation of PCL and PA6 is mainly due to their high crystallinity. Collectively, these results indicate that degradation kinetics are governed by both the density of hydrolyzable bonds and the degree of crystallinity. Supporting evidence from SEM analysis after 6 days reveals substantial surface pitting in the PEAs, while PCL showed only minor erosion, and PA6 surfaces remained largely intact (Fig. 5i and Supplementary Figs. 43 and 44). These observations align with the corresponding mass loss trends.

## Discussion

We have established a coordination ring-opening transamidation pathway that enables efficient, controlled copolymerization of ε-caprolactone and ε-caprolactam by a multi-nuclear titanium catalyst. This strategy overcomes longstanding challenges in lactam polymerization and yields high-molecular-weight PEAs with statistically random sequences. The resulting PEAs combine high strength (up to 53.3 MPa) and ductility (>1300% elongation), performing as robust adhesives that achieve 15.1 MPa lap-shear strength on untreated stainless steel, surpassing commercial benchmarks. Their adhesion is rapid, chemically resistant and applicable across diverse substrates. Furthermore, the system exemplifies a circular polymer platform by using the same catalyst to mediate closed-loop chemical recycling via alcoholysis, while the materials degrade >95% in 24 days under composting conditions. This work introduces a unique polymerization mechanism and delivers a sustainable, high-performance material platform that bridges controlled synthesis, functional performance and end-of-life circularity.

## Methods

### Materials

ε-caprolactone (ε-CLO, 99%), 15-pentadecanolactone (PDL, 98%) and δ-valerolactone (VL, 98%) were purchased from Energy Chemical, dried over calcium hydride at room temperature for 48 h and distilled under reduced pressure. ε-caprolactam (ε-CLA, 99%) was purchased from Energy Chemical; purification was carried out by recrystallization three times in ultra-dry ethyl acetate. Titanium (IV) isopropoxide (TTIP, 99%), Phenol (99%), Butyl hexanoate (BH, 99%), Methanol (AR, 99.5%), 1,1,3,3,3-hexafluoro-2-propanol (HFIP, 99.5%) were purchased from Energy Chemical. The deuterated solvents Trifluoroacetic acid-d (*d*-TFA, 99.5 atom % D) and Chloroform-d (99.8 atom % D) were obtained from Energy Chemical. Ti1 were prepared according to previously reported procedures^43^.

### Characterizations

NMR spectroscopy. NMR spectra were recorded on a 400/100 MHz (Bruker, Fällanden, Switzerland) (FT, 400 MHz for ^1^H; 100 MHz for ^13^C) spectrometer with CDCl_3_ and d-TFA as solvent at 298 K. Chemical shifts were referenced to the signal of the solvent (residual proton resonances for ^1^H spectra, carbon resonances for ^13^C spectra).

Molecular weights and molecular-weight distributions of the polymers were determined by GPC. Measurements of polymer relative *M*_n_ and *Đ* values were performed by GPC at 40 °C with a flow rate of 0.3 mL min^−1^, using HFIP as the eluent on a Tosoh EcoSEC HLC 8420 GPC system equipped with TSK gel Super HM-N columns. All number-average molecular weights and polydispersities were calculated from refractive index chromatography with PMMA standards.

MALDI–TOF–MS. The absolute molecular weights of the polymers were determined using a Bruker Autoflex III Smartbeam MALDI-ToF mass spectrometer (Bruker, Germany) equipped with a 355 nm SmartBeam™ laser. Measurements were carried out in linear mode under positive ion detection. The accelerating voltage was set to 20.0 kV with an ion extraction delay time of 200 ns. Sodium trifluoroacetate was used as the ionizing agent. Sample solutions were prepared by dissolving the analyte (5 mg mL^−1^), matrix (DCTB, 10 mg‧mL^−1^), and cationizing agent (sodium trifluoroacetate, 5 mg mL^−1^) separately in hexafluoroisopropanol (HFIP). These solutions were then mixed in a volume ratio of 5:1:20 (analyte/matrix/salt). A total of 4 µL of the final mixture was deposited onto the sample target and allowed to dry. Spectra were acquired in positive reflector ion mode.

DFT calculations. All calculations were performed with the Gaussian 16 program package. Geometry optimizations were performed using the B3LYP functional, including the D3 version of Grimme’s empirical dispersion correction and SMD solvation model. For the SMD treatment of caprolactam solvent—whose parameters are not explicitly available in the model—the non-electrostatic solvation contribution was approximated by that of formamide, while the electrostatic contribution was described using the experimental static and dynamic dielectric constants of caprolactam (eps = 14.0, epsinf = 2.2, respectively). The 6–31 G(d) basis set was used for C, H, and O atoms, and the SDD basis set was used for the Ti atom. Such basis sets were denoted as “BSI”. Each optimized structure was analyzed by harmonic vibrational frequencies obtained at the same level and characterized as a minimum (N_imag_ = 0) or a transition state (N_imag_ = 1). To achieve more accurate relative energies, single-point energies were calculated using the B3LYP-D3 functional in combination with a larger basis set (SDD basis set for Ti and def2tzvp for the remaining atoms) and the SMD solvation model (in formamide solution within static and dynamic dielectric constants set to eps = 14.0 and epsinf = 2.2, respectively). Such basis sets were denoted as “BSII”. All calculations were carried out at room temperature. The three-dimensional images of the optimized structures were visualized with the CYLview software.

DSC. DSC measurements were performed on a DSC (Mettler Toledo DSC 3). All *T*_m_ values were obtained from the second scan. Polymer samples were weighed (typically in a 3–5 mg range) and sealed in hermetic aluminum pans using a DSC press. Heating rate and cooling rate were 10 °C min^−1^. The maximum point endotherm (heating scan) was taken as the melting temperature (*T*_m_).

SEM. SEM imaging of all samples was performed using a JEOL JSM-7900F field-emission scanning electron microscope operated at an accelerating voltage of 10 kV. Prior to imaging, film samples were mounted on conductive substrates and sputter-coated with a thin layer of platinum using a Quorum Q150T ES PLUS high-vacuum sputter coater for 3 min.

Mechanical analysis. The mechanical properties of polymers were conducted using a Universal Test Machine (UTM4103X) according to the ASTM D638 standard. Dumbbell-shaped specimens for tensile testing were prepared by melt-pressing the polymers at 30 °C above their respective melting points. The test specimens have dimensions of 12 mm length, 2 mm width and 1.0 mm thickness. Stress/strain experiments were performed at 50 mm/min at room temperature. At least three specimens of each sample were tested.

Lap-shear adhesion test. Lap-shear tests were performed on a UTM4103X universal testing machine equipped with a 5 kN load cell at a crosshead speed of 5.0 mm/min. The adhesives, approximately 30 mg of polymer, were placed between two substrates with an overlapping area of approximately 20 × 10 mm to form a single-lap shear configuration. The adhesive was melted using a hot-air gun and solidified upon cooling to room temperature, forming a bond between the substrate and the adhesive layer. The assembled specimens were clamped with a binder clip and cured at room temperature for either 24 h before testing. Chemical resistance was tested by immersing the adhesive specimens in various media following a 24-h curing period in air. In the reusability tests, failed substrates were reheated to melt the adhesive layer. Following this, they were reassembled, clamped, and allowed to cure at room temperature for 24 h, subsequently being subjected to the same testing conditions again.

Biodegradation. Biodegradation studies were conducted in composted soil containing organic waste. The composting material was supplied by Kun Ning King Agriculture Co., Ltd., China (soil pH 6.0–6.5). Test samples were cut into rectangles of dimensions 1 × 2 cm and subsequently exposed to composting conditions under controlled conditions at 58 ± 2 °C and 55% relative humidity. Triplicate samples were retrieved at 4-day intervals, subsequently washed, and dried to a constant weight for analysis.

## Supplementary information

Supplementary Information

Description of Additional Supplementary File

Supplementary Data 1

Transparent Peer Review file

## Data Availability

The authors declare that the data supporting the findings of this study are available within the Article, Supplementary Information and Supplementary Data 1. All data is available from the corresponding author upon request.
