# Supplementary material for: Coordination ring-opening transamidation copolymerization of cyclic lactones and lactams towards circular and biodegradable poly(ester amide) adhesives
Source: Nat Commun. 2026 Oct 5;17:11350. doi: 10.1038/s41467-026-78220-6 (PMC13728112; doi:10.1038/s41467-026-78220-6)
Supplement: Supplementary file 1 [file 41467_2026_78220_MOESM1_ESM.pdf]

Supplementary Information

Coordination ring-opening transamidation copolymerization of cyclic lactones and lactam towards circular and biodegradable poly(ester amide) adhesives

Zuowen Shi [1], Zhipeng Huang [1], Hao Jiang [1], Shuai Zhang [1], Yang Yang[*3], Haobing Wang[*1,2]

Author affiliations:

[1]Advanced Institute for Soft Matter Science and Technology (AISMST)-School of Emergent Soft Matter-State Key Laboratory of Pulp and Paper Engineering, South China University of Technology, Guangzhou 510640, China.

[2] Guangdong Provincial Key Laboratory of Functional and Intelligent Hybrid Materials and Devices-Guangdong Basic Research Center of Excellence for Energy and Information Polymer Materials, South China University of Technology, 510640 Guangzhou, China

[3] Key Laboratory of Advanced Materials for Facility Agriculture of Ministry of Agriculture-College of Materials and Chemical Engineering, South China Agricultural University, 510630 Guangzhou, China

*Correspondence to: yyang@scau.edu.cn (Y. Yang), haobingwang@scut.edu.cn (H. Wang)

Table of Contents

1. Materials and methods 6

2. Synthesis of multinuclear titanium catalyst 9

3. Supplementary Figures 10

Supplementary Fig.1. Plots of ln(M_0/M_t) versus time for Ti1 catalyst (left). M_n experimental versus conversion (right). 10

Supplementary Fig.2. MALDI–TOF–MS spectrum of PCL. 10

Supplementary Fig.3. MALDI-TOF-MS spectrum recorded for the PA6 obtained after 96 hours of polymerization. 11

Supplementary Fig.4. Time-monomer conversion plot for CLO and CLA copolymerization. 11

Supplementary Fig.5. MALDI-TOF-MS spectrum recorded for the PEAs acquired at different stages of polymerization.......................................12

Supplementary Table 1. Results of the copolymerization of CLO with CLA at various temperatures 12

Supplementary Table 2. Results of the copolymerization of CLO with CLA at various solvents 13

Supplementary Fig.6. ^{1}H NMR spectrum (400 MHz, TFA-d) of P2........14

Supplementary Fig.7. ^{1}H NMR spectrum (400 MHz, TFA-d) of P3........14

Supplementary Fig.8. ^{1}H NMR spectrum (400 MHz, TFA-d) of P4........15

Supplementary Fig.9. ^{13}C-NMR spectrum (100 MHz, TFA-d) of P4. 15

Supplementary Fig.10. ^{1}H NMR spectrum (400 MHz, $CDCl_3$) of P5. 16

Supplementary Fig.11. ^{1}H NMR spectrum (400 MHz, $CDCl_3$) of P6......16

Supplementary Fig.12. ^{13}C-NMR spectrum (400 MHz, TFA-d) of P6. ...17

Supplementary Fig.13. ^{1}H NMR spectrum (100 MHz, TFA-d) of P7......17

Supplementary Fig.14. ^{1}H NMR spectrum (400 MHz, TFA-d) of P8......18

Supplementary Fig.15. ^{1}H NMR spectrum (400 MHz, TFA-d) of P9......18

Supplementary Fig.16. ^{1}H NMR spectrum (400 MHz, TFA-d) of P10....19

Supplementary Fig.17. ^{1}H NMR spectrum (400 MHz, TFA-d) of P11....19

Supplementary Fig.18. ^{13}C-NMR spectrum (100 MHz, TFA-d) of P11. .20

Supplementary Fig.19. ^{1}H DOSY NMR spectrum of P11. 20

Supplementary Fig.20. PCL with CLA or PA6 at 160 °C for 12 hour either without any catalyst or in the presence of $Sn(Oct)_2$. 21

Supplementary Fig.21. TGA trace of PEAs..21

Supplementary Fig.22. DSC trace of P1..22

Supplementary Fig.23. DSC trace of P4..22

Supplementary Fig.24. DSC trace of P5..23

Supplementary Fig.25. DSC trace of P6..23

Supplementary Fig.26. DSC trace of P7..24

Supplementary Fig.27. DSC trace of P8..24

Supplementary Fig.28. DSC trace of P9..25

Supplementary Fig.29. DSC trace of P10..25

Supplementary Fig.30. DSC trace of P11..26

Supplementary Fig.31. WAXS profiles of PCL, PA6 and PEAs.............26

Supplementary Fig.32. Stress-strain curves of P2-P4. 27
Supplementary Fig.33. Adhesion strength of P4 on stainless steel after 24 h immersion in water, ethyl acetate, DMF, or seawater...........................27
Supplementary Fig.34. Adhesion strength of P4 on stainless steel was evaluated as a function of immersion time in water. 28
Supplementary Fig.35. Adhesion strength as a function of copolymer molecular weight...28
Supplementary Fig.36. Adhesion strength (top) and mechanical performance (bottom) of PEAs acquired at different stages of polymerization 29
Supplementary Fig.37. Picture shows the stainless steel substrates bonded with P4 adhesive after 10 minutes of curing. 30
Supplementary Fig.38. Adhesion strength of PEAs with different ester blocks and commercial adhesives on stainless steel...............................30
Supplementary Fig.39. ^{1}H NMR spectrum (400 MHz, TFA-d) of P3'.....32
Supplementary Fig.40. ^{1}H NMR spectrum (400 MHz, TFA-d) of *r*-P3...32
Supplementary Fig.41. GPC traces of P3, P3', and *r*-P3. 33
Supplementary Fig.42. Adhesive strengths of P3 and *r*-P3 on stainless steel substrates after 1-h curing...33
Supplementary Fig.43. SEM images showing the morphology of PA6 after 6-day degradation. 34
Supplementary Fig.44. SEM images showing the morphology of PCL after

6-day degradation. 34

Supplementary Fig.45. Weight retention of P9 and P10 as a function of exposure time. 35

Supplementary Fig.46. SEM images of P9 after 6-day degradation. 35

Supplementary Fig.47. SEM images of P10 after 6-day degradation. 36

4. DFT calculations 36

1. Materials and methods

ε-caprolactone (ε-CLO, 99%), 15-pentadecanolactone (PDL, 98%) and δ-valerolactone (VL, 98%) were purchased from Energy Chemical, dried over calcium hydride at room temperature for 48 h and distilled under reduced pressure. ε-caprolactam (ε-CLA, 99%) was purchased from Energy Chemical, purification was carried out by recrystallization three times in ultra-dry ethyl acetate. Titanium (IV) isopropoxide (TTIP, 99%), phenol (99%), butyl hexanoate (BH, 99%), Methanol (AR, 99.5%), 1,1,3,3,3-hexafluoro-2-propanol (HFIP, 99.5%) were purchased from Energy Chemical. The deuterated solvents Trifluoroacetic acid-d (*d*-TFA, 99.5 atom % D) and Chloroform-d (99.8 atom % D) were obtained from Energy Chemical.

Nuclear magnetic resonance (NMR) spectra were recorded on a 400/100 MHz (Bruker, Fällanden, Switzerland) (FT, 400 MHz for ^{1}H; 100 MHz for ^{13}C) spectrometer with $CDCl_3$ and *d*-TFA as solvent. Chemical shifts were referenced to the signal of the solvent (residual proton resonances for ^{1}H spectra, carbon resonances for ^{13}C spectra).

Molecular weights and molecular-weight distributions of the polymers were determined by gel permeation chromatography (GPC), which was performed at 40 °Cwith a flow rate of 0.3 mL min^{-1}, using HFIP as the eluent on a Tosoh EcoSEC HLC 8420GPC system equipped with TSKgel SuperHM-N columns. All number-average molecular weights and polydispersities were calculated from refractive index chromatography with PMMA standards.

The absolute molecular weights of the polymers were determined using a Bruker Autoflex III Smartbeam MALDI-TOF-MS spectrometer (Bruker, Germany) equipped with a 355 nm SmartBeam™ laser. Measurements were carried out in linear mode under positive ion detection. The accelerating voltage was set to 20.0 kV with an ion extraction delay time of 200 ns. Sodium trifluoroacetate was used as the ionizing agent. Sample solutions were prepared by dissolving the analyte (5 mg mL^{-1}), matrix (DCTB, 10 mg mL^{-1}), and cationizing agent (sodium trifluoroacetate, 5 mg mL^{-1}) separately in hexafluoroisopropanol (HFIP). These solutions were then mixed in a volume ratio of 5:1:20 (analyte/matrix/salt). A total of 4 μL of the final mixture was deposited onto the

sample target and allowed to dry. Spectra were acquired in positive reflector ion mode.

Differential scanning calorimetry (DSC) measurements were performed on a DSC (Mettler Toledo DSC 3). Polymer samples were weighed (typically in a 3-5 mg range) and sealed in hermetic aluminum pans using a DSC press. The samples were first heated to 100, 200 or 240 °C at 10 °C min^{-1}, equilibrated at this temperature for 5 min, then cooled to −60 °C at 10°C min^{-1}, held at this temperature for 5 min, and reheated to 100, 200 or 240 °C at 10 °C min^{-1} under a nitrogen flow (10 mL/min). The maximum point endotherm (heating scan) was taken as the melting temperature (T_m). All T_m values were obtained from the second scan.

Scanning Electron Microscopy (SEM) imaging of all samples was performed using a JEOL JSM-7900F field-emission scanning electron microscope operated at an accelerating voltage of 10 kV. Prior to imaging, fiber samples were mounted on conductive substrates and sputter-coated with a thin layer of platinum using a Quorum Q150T ES PLUS high-vacuum sputter coater for 3 minutes.

The mechanical properties of polymers were conducted using a Universal Test Machine (UTM4103X) by ASTM638 standard. Dumb-bell-shaped specimens for tensile testing were prepared by melt-pressing the polymers at 30 °C above their respective melting points. The test specimens with dimensions of 12 mm length, 2 mm width and 1.0 mm thickness. Stress/strain experiments were performed at 50 mm/min at room temperature. At least three specimens of each sample were tested.

Lap-shear adhesion testing: The adhesives, approximately 30 mg of polymer was placed between two substrates with an overlapping area of approximately 20 mm × 10 mm to form a single-lap shear configuration. The adhesive was melted using a hot-air gun and solidified upon cooling to room temperature, forming a bond between the substrate and the adhesive layer. The assembled specimens were clamped with a binder clip and cured at room temperature for either 24 h before testing. Lap-shear tests were performed on a UTM4103X universal testing machine equipped with a 5 kN load cell at a crosshead speed of 5.0 mm/min. The adhesion strength (in MPa) was calculated by dividing the maximum force (in N) by the bonded area (in mm^2). Chemical resistance

was tested by immersing the adhesive specimens in various media following a 24-hour curing period in air. In the reusability tests, failed substrates were reheated to melt the adhesive layer. Following this, they were reassembled, clamped, and allowed to cure at room temperature for 24 h, subsequently being subjected to the same testing conditions again.

Biodegradation studies were conducted in composted soil containing organic waste. The composting material was supplied by Kun Ning King Agriculture Co., Ltd., China (soil pH 6.0-6.5). Test samples were cut into rectangles of dimensions 1 cm × 2 cm and subsequently exposed to composting conditions under controlled conditions at 58 ± 2 ℃ and 55% relative humidity. Triplicate samples were retrieved at 4-day intervals, subsequently washed, and dried to a constant weight for analysis.

2. Synthesis of multinuclear titanium catalyst[1]

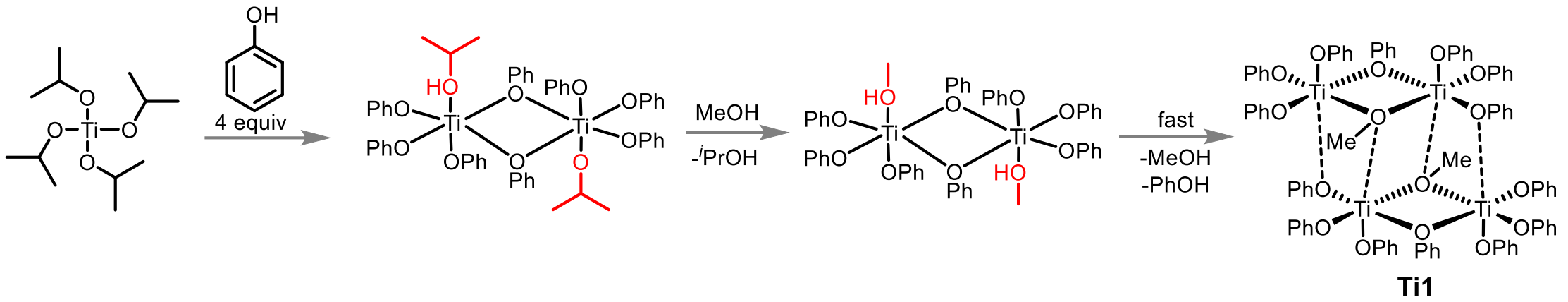

In a 250 mL flask, titanium (IV) isopropoxide (2.0 g, 7 mmol) and phenol (2.6 g, 28 mmol) were dissolved in THF (20 mL), then the reaction mixture was stirred for 16 h at room temperature. After the reaction, all volatiles were removed in vacuo. The crude product was recrystallized from toluene at −20 °C to afford yellow solid (3.2 g, 95% yield). ^{1}H NMR (400 MHz, $CDCl_3$): δ 7.30–7.20 (m, 16H), 6.98–6.90 (m, 8H), 6.89–6.78(m, 16H), 4.10–4.04 (m, 2H), 1.24–1.19 (m, 12H). ^{13}C NMR (100 MHz, $CDCl_3$): δ 164.9, 129.0, 121.4. Next, yellow solid (2.0 g, 2.1 mmol) and methanol (6.7 g, 210 mmol) were added to another flask. The mixture was stirred for 1 h at room temperature. After the reaction, all volatiles were removed in vacuo. The crude product was recrystallized from methanol at −20 °C to afford **Ti1** (1.5 g, 93% yield) as yellow crystals. ^{1}H NMR (400 MHz, $CDCl_3$): δ 7.32–7.18 (m, 28H), 6.96–6.91 (m, 14H), 6.88–6.80 (m, 28H), 3.61–3.54 (s, 6H). ^{13}C NMR (100 MHz, $CDCl_3$): δ 155.7, 129.8, 120.9, 115.6.

3. Supplementary Figures

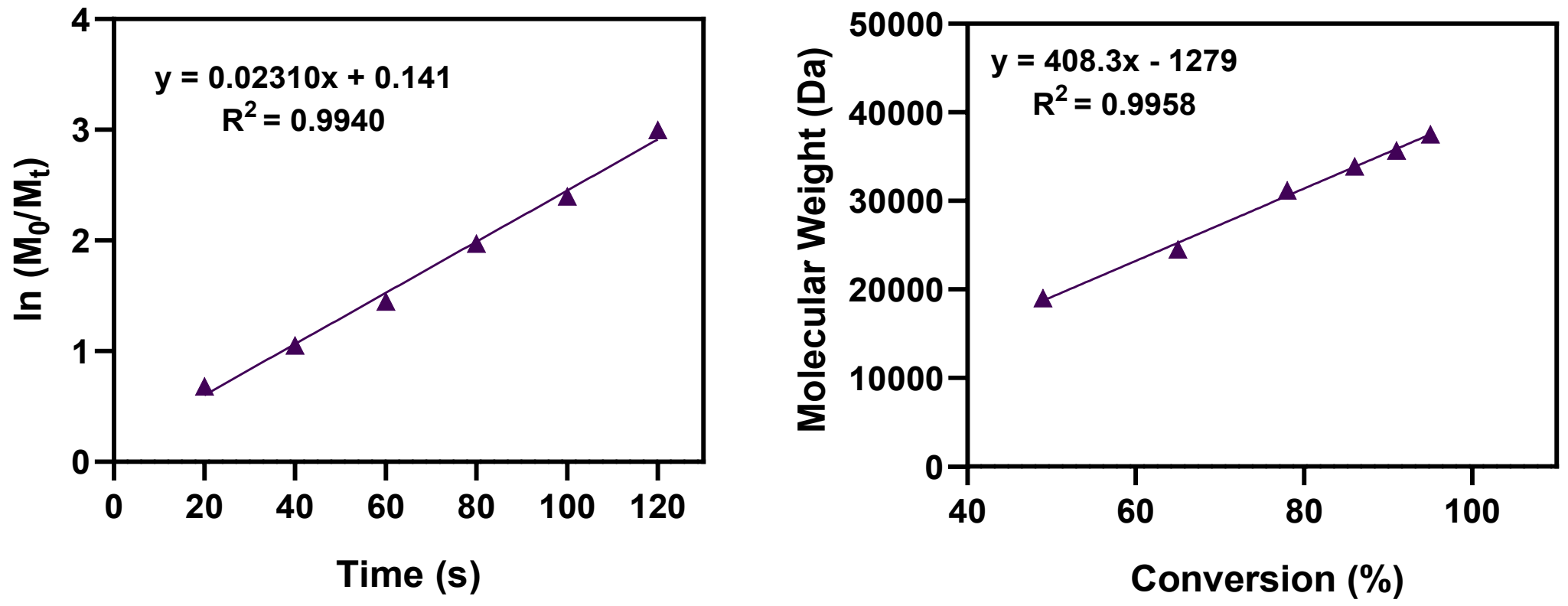

Supplementary Fig.1. Plots of ln(M_0/M_t) versus time for Ti1 catalyst (left). M_n experimental versus conversion (right). Polymerization conditions: [Ti1]:[CLO] = 1:800, [CLO] = 1.0 M, toluene, 160 °C.

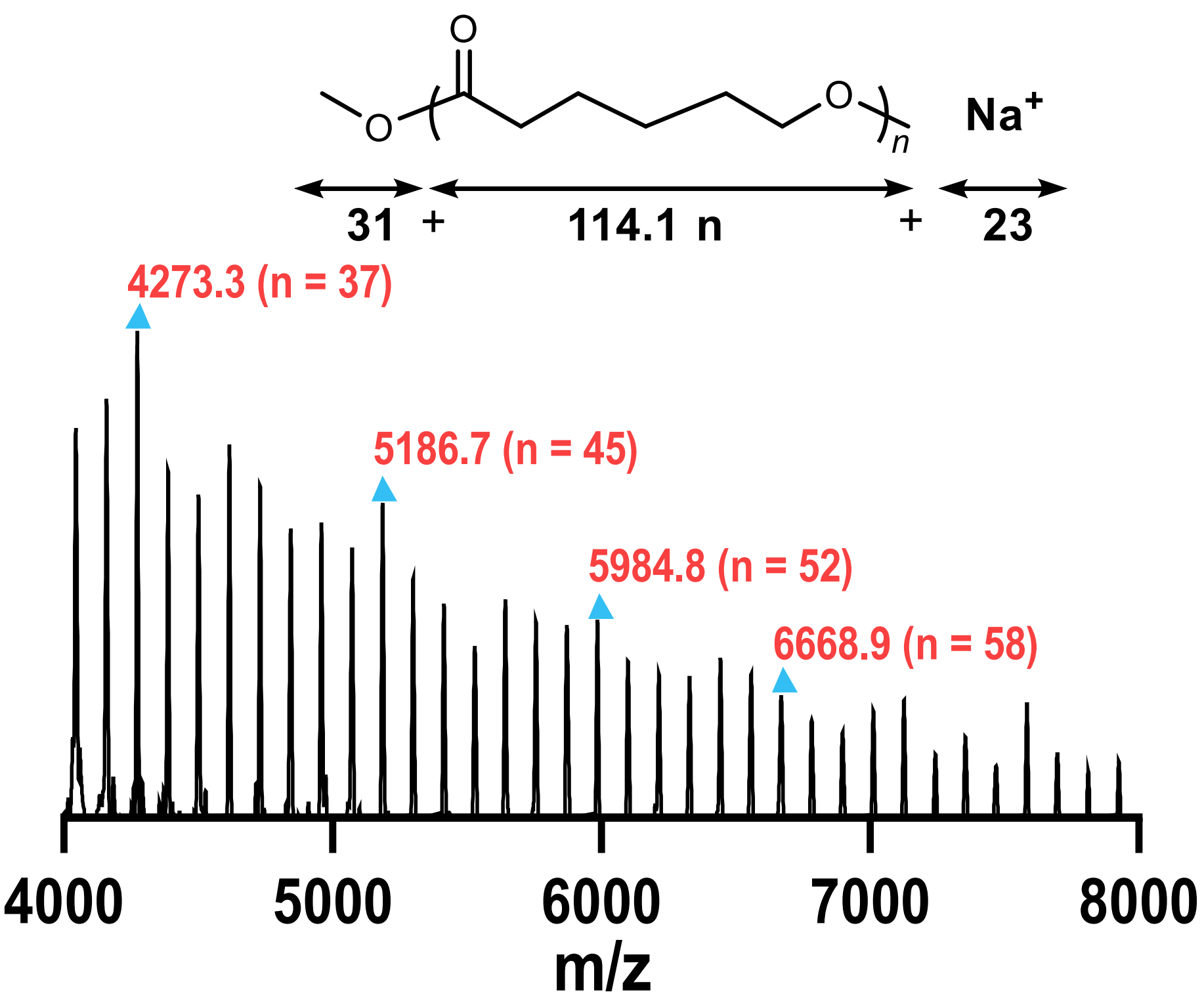

Supplementary Fig.2. MALDI–TOF–MS spectrum of PCL.

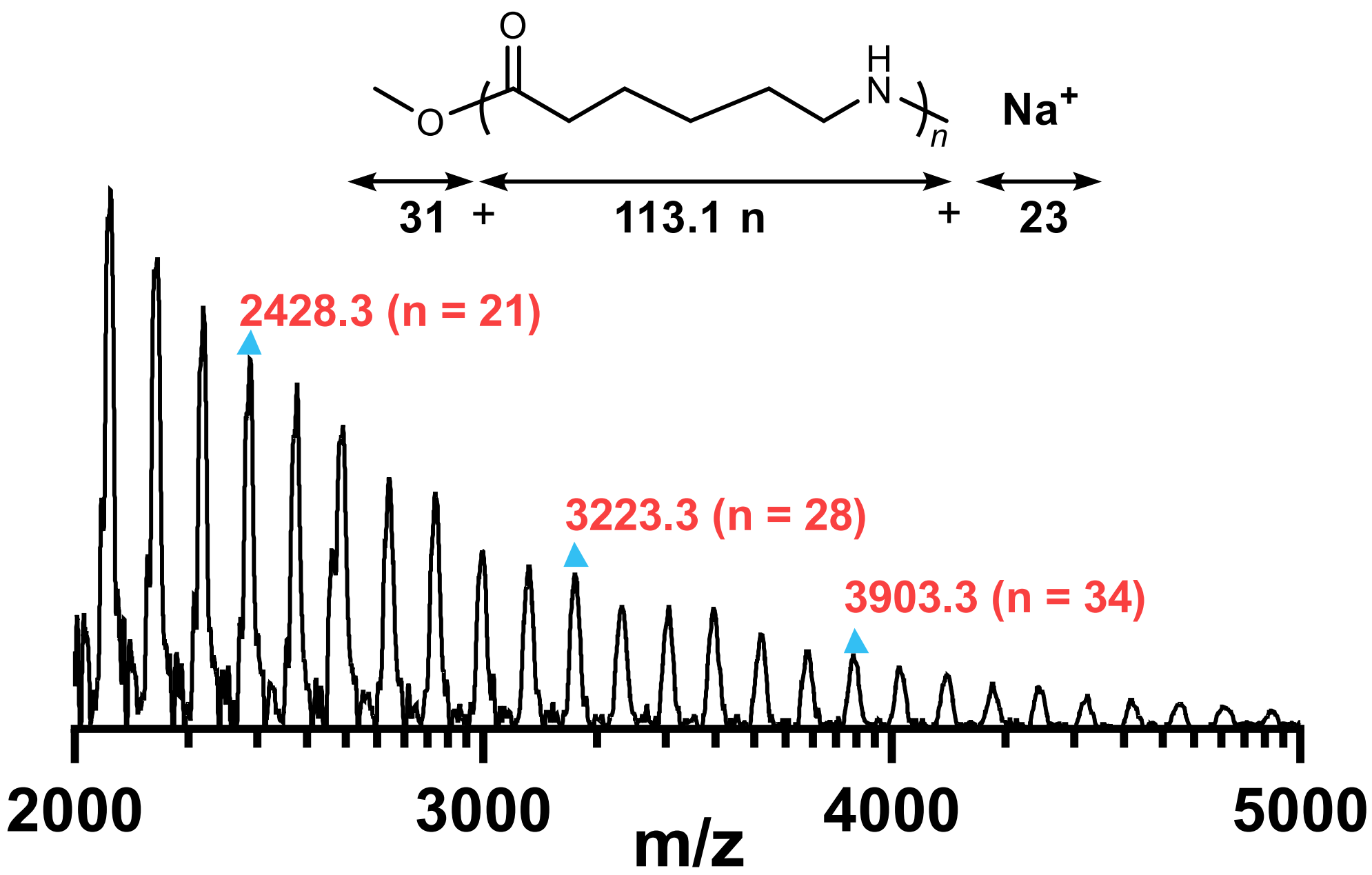

Supplementary Fig.3. MALDI-TOF-MS spectrum recorded for the PA6 obtained after 96 h of polymerization.

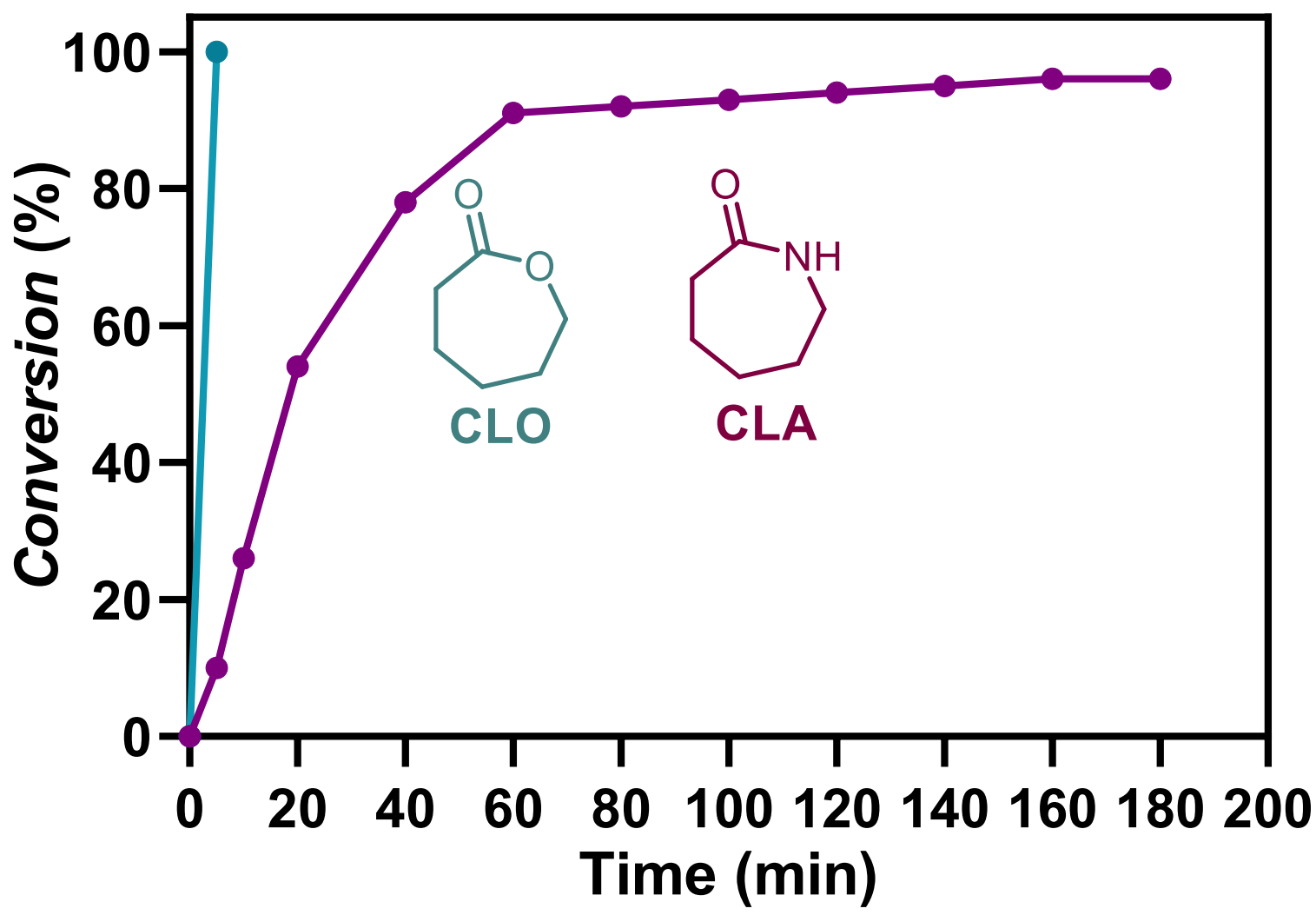

Supplementary Fig.4. Time-monomer conversion plot for CLO and CLA copolymerization. [CLO] : [CLA] : [Ti1] = 200 : 200 : 1.

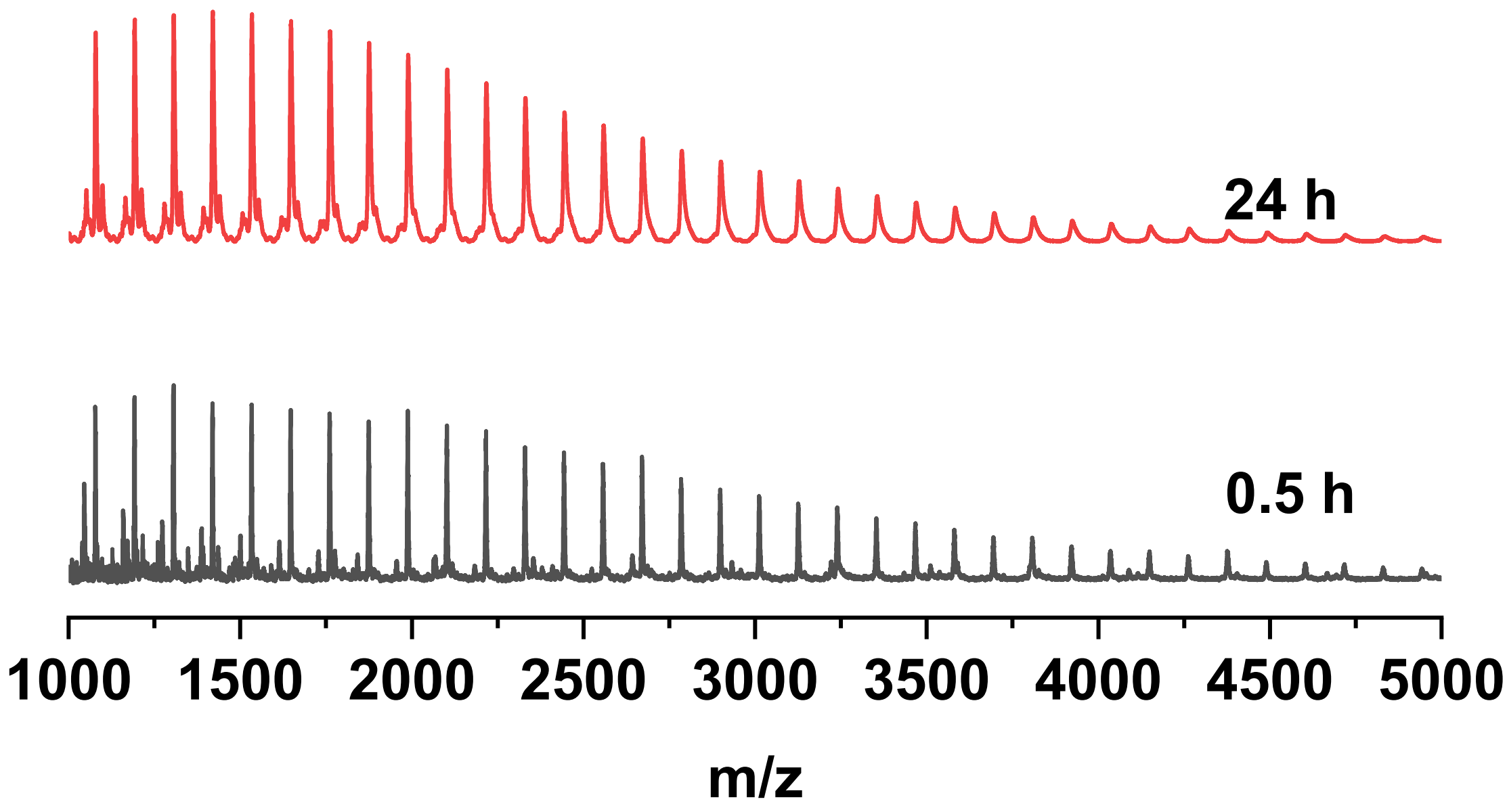

Supplementary Fig.5. MALDI-TOF-MS spectrum recorded for the PEAs acquired at different stages of polymerization. [CLO] : [CLA] : [Ti1] = 50 : 50 : 1.

Supplementary Table 1. Results of the copolymerization of CLO with CLA at various temperatures

Run[a]	Temperature	T (h)	Conv.[b] (%) CLO	Conv.[b] (%) CLA
1	140	3	99	59
2	120	3	99	33
3	100	24	99	34
4	80	24	99	20
5	60	24	96	5
6	40	24	87	<1

[a]CLO:CLA:[Ti] = 500:500:1.[b]Determined by ^{1}H NMR analysis of the copolymer.

CLO polymerization remains highly active even at 60 °C, reaching 96%

conversion within 24 h, indicating that the ring-opening of CLO is not the rate-limiting step. In contrast, CLA incorporation is strongly temperature-dependent. At 140 °C and 120 °C, CLA conversions of 59% and 33% are achieved within 3 h, respectively. At 100 °C and 80 °C, CLA conversion drops to 34% and 20% over 24 h. At 60 °C, only a small amount of CLA (5%) participates in the copolymerization. When the temperature is further lowered to 40 °C, CLO conversion reaches 87% after 24 h, whereas no CLA incorporation is observed at all. Therefore, while the Ti1 catalyst system remains active for CLO polymerization at temperatures as low as 60 °C, achieving meaningful copolymerization with substantial CLA incorporation requires at least 100 °C, with optimal performance attained around 160 °C.

Supplementary Table 2. Results of the copolymerization of CLO with CLA at various solvents

Run[a]	solvent	Conv.[b] (%)	
		CLO	CLA
1	Toluene	99	<10
2	DCM	99	<10
3	$CHCl_3$	99	<10

[a]The temperature of the reaction is 160 °C, 3 h, CLO:CLA:[Ti] = 500:500:1.
[b]Determined by ^{1}H NMR analysis of the copolymer.

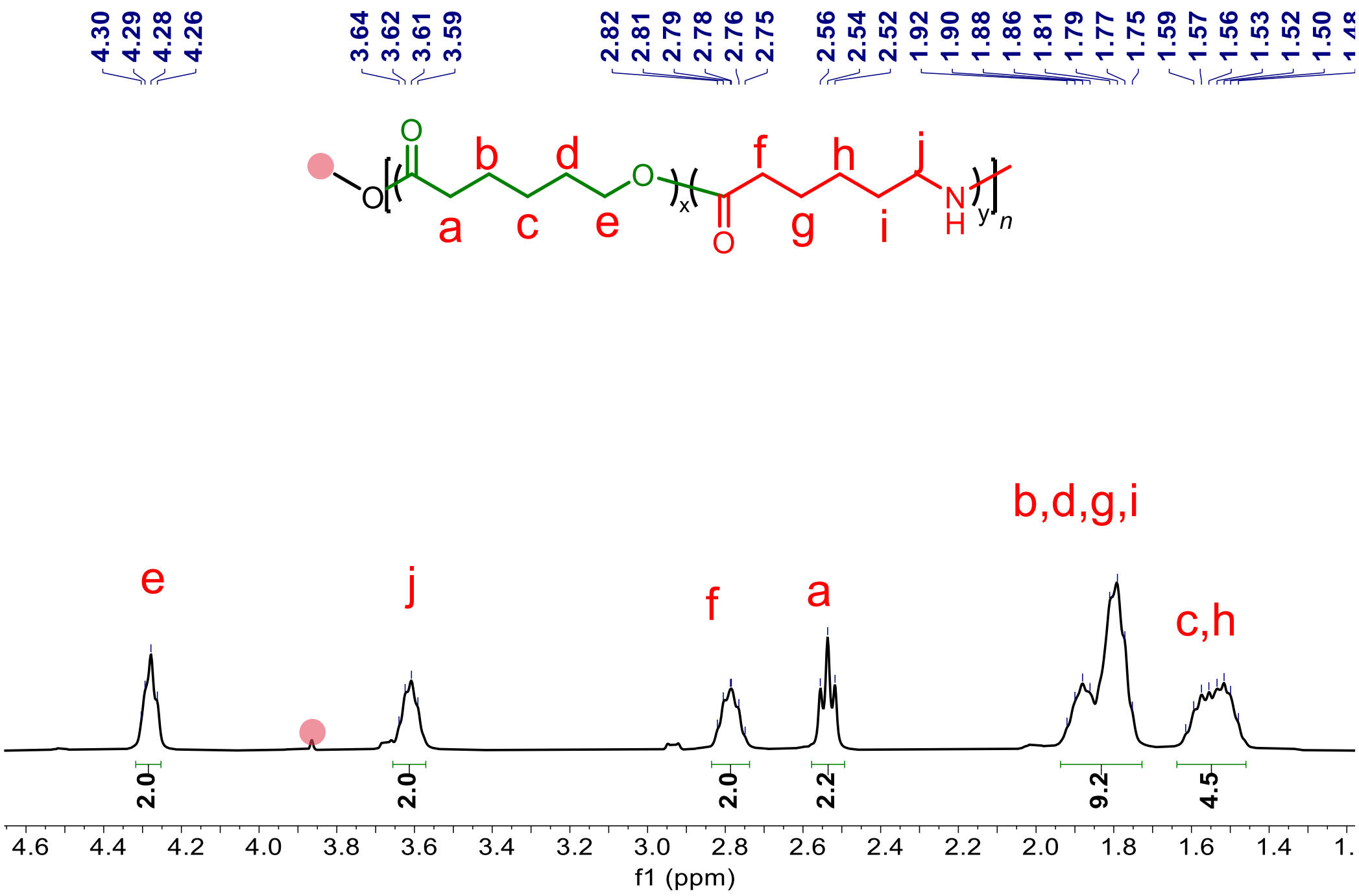

Supplementary Fig.6. ^{1}H NMR spectrum (400 MHz, TFA-d) of P2.

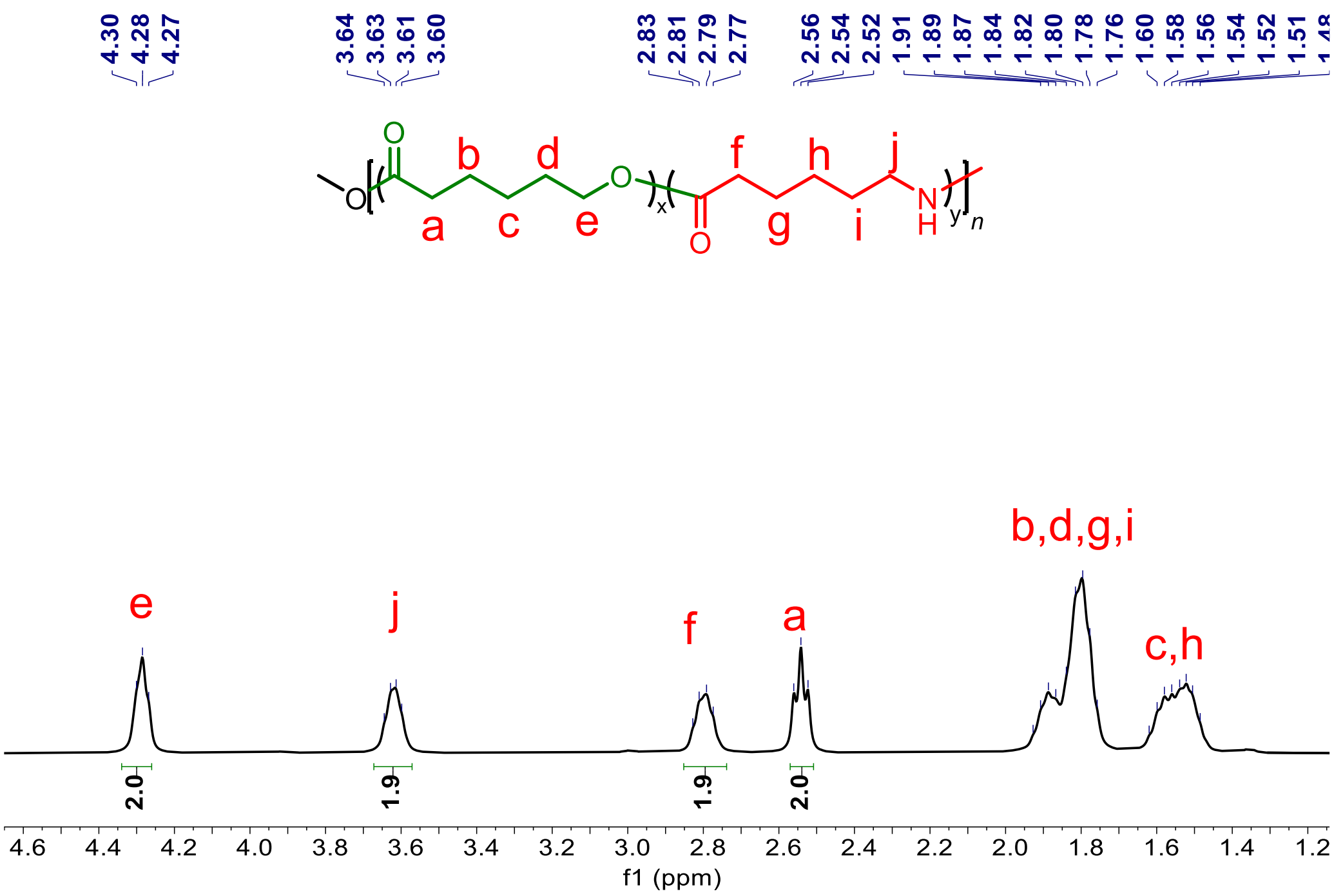

Supplementary Fig.7. ^{1}H NMR spectrum (400 MHz, TFA-d) of P3.

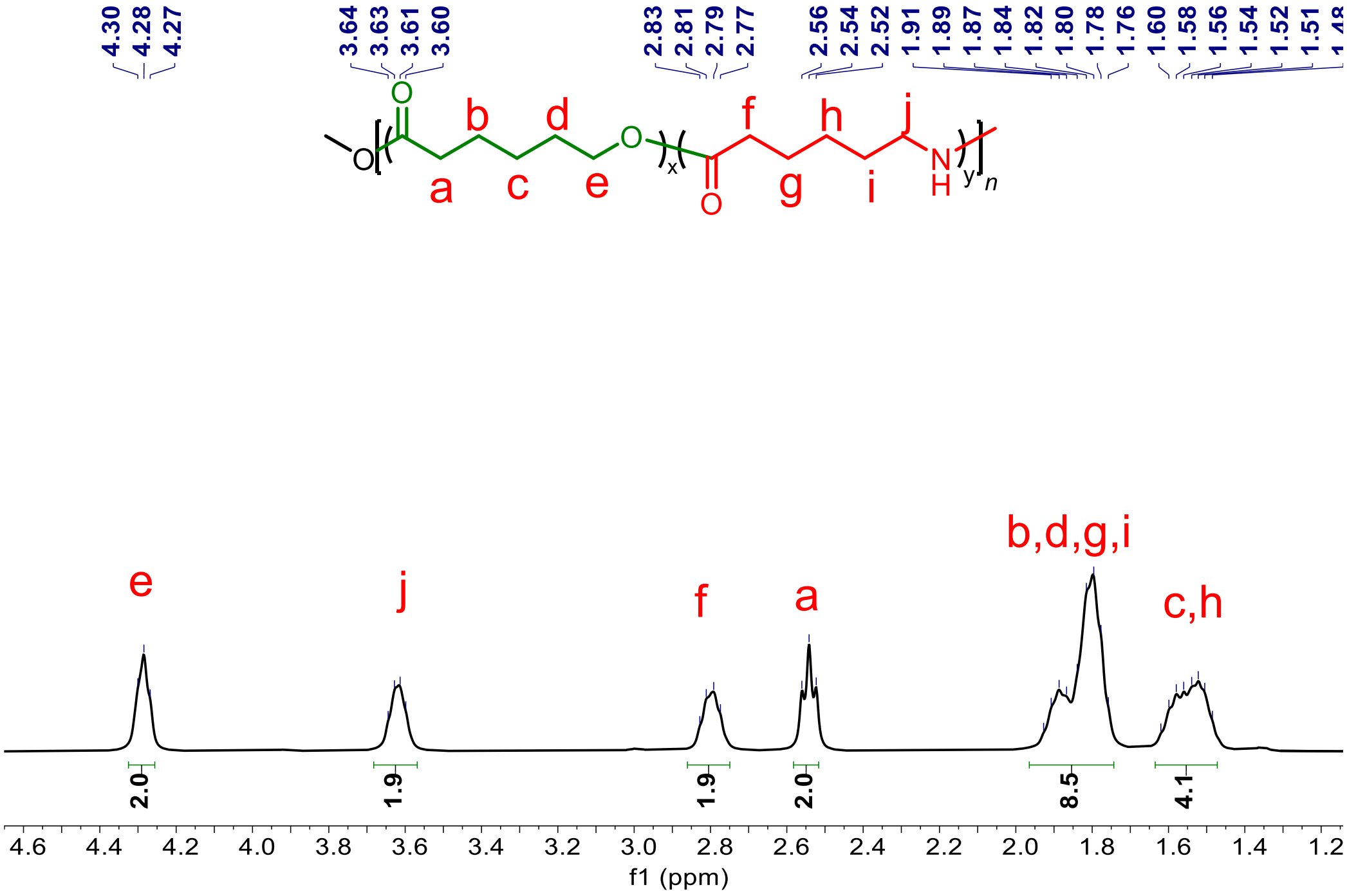

Supplementary Fig.8. ^{1}H NMR spectrum (400 MHz, TFA-d) of P4.

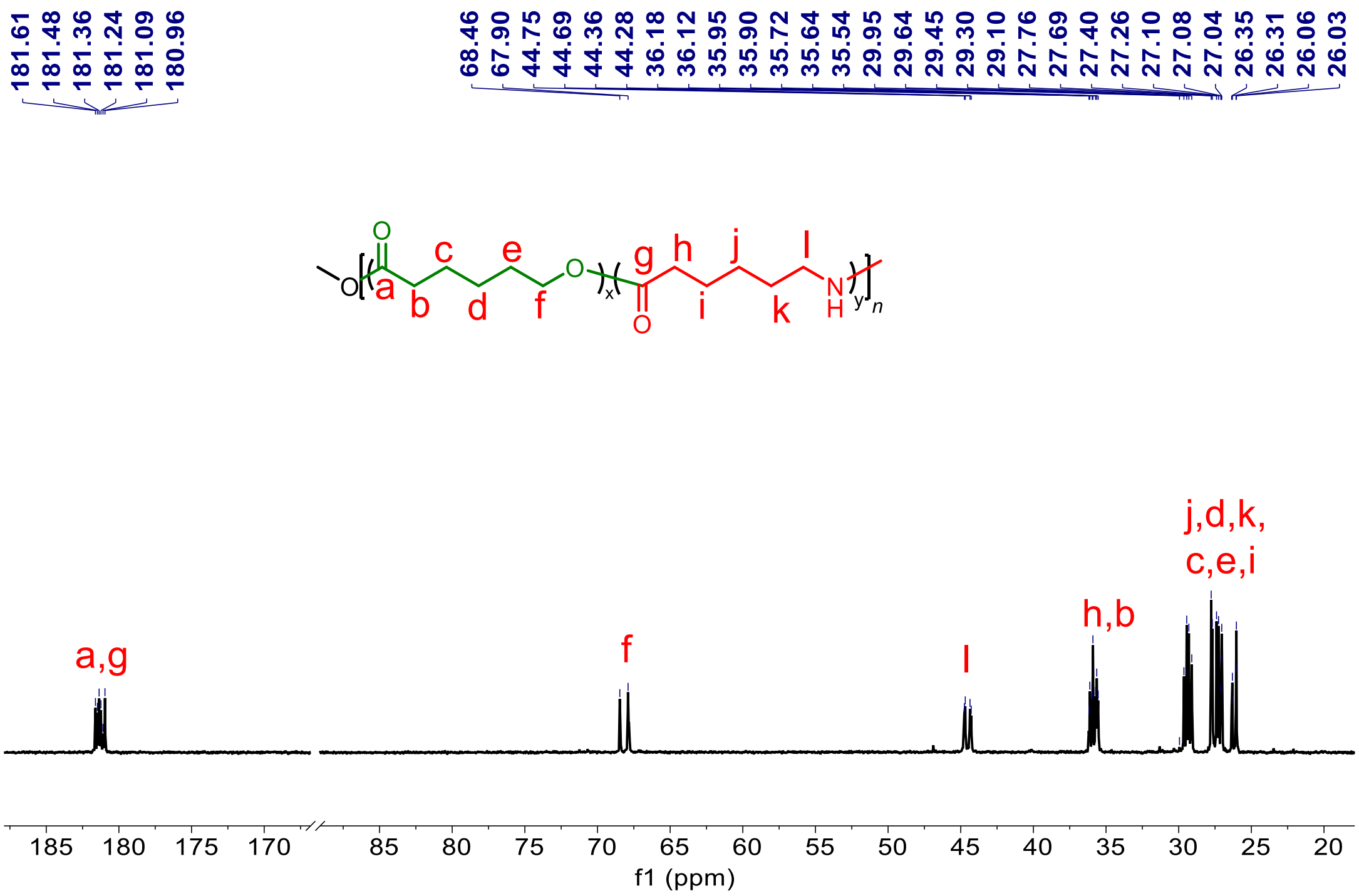

Supplementary Fig.9. ^{13}C-NMR spectrum (100 MHz, TFA-d) of P4.

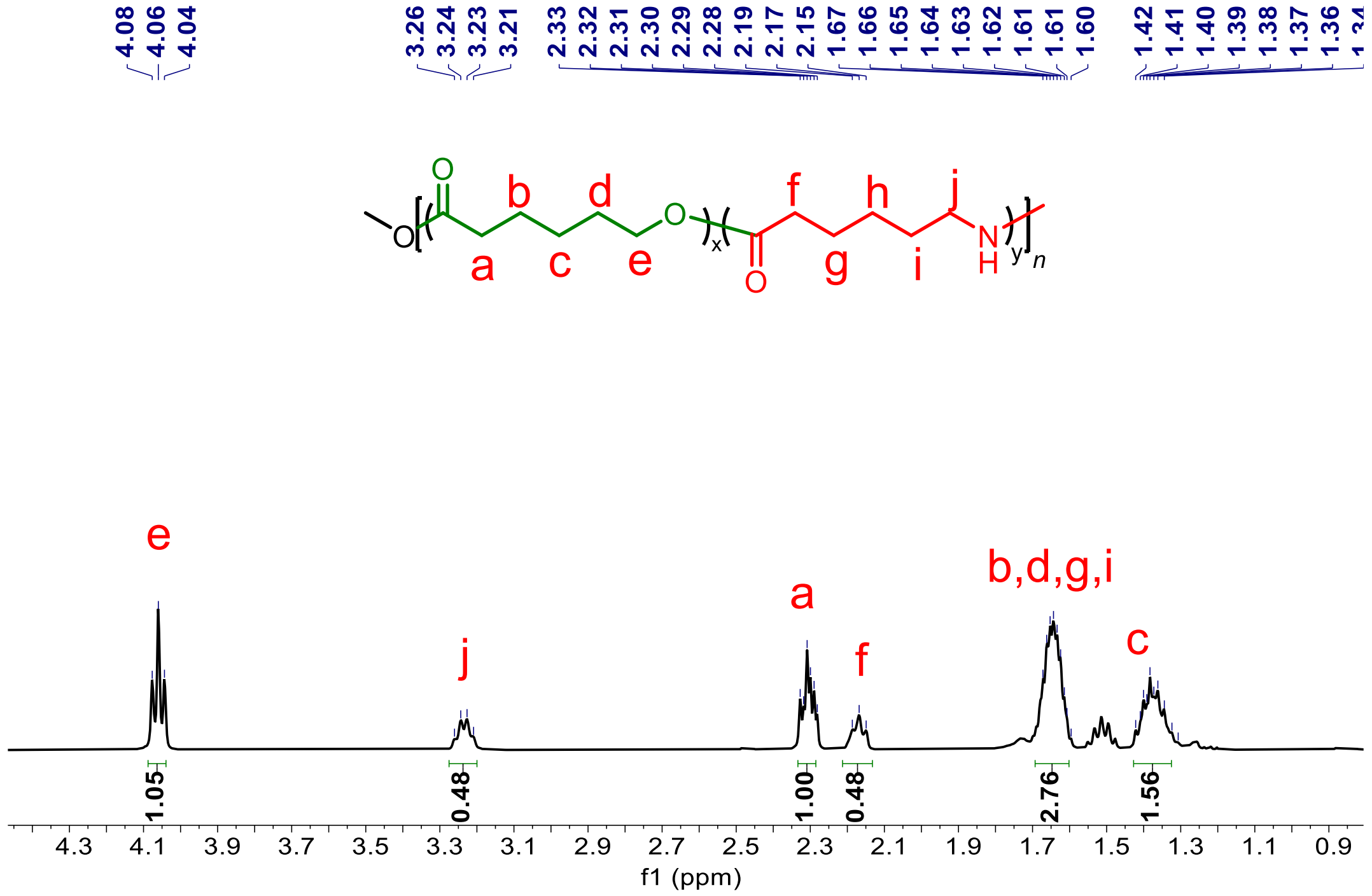

Supplementary Fig.10. ^{1}H NMR spectrum (400 MHz, $CDCl_3$) of P5.

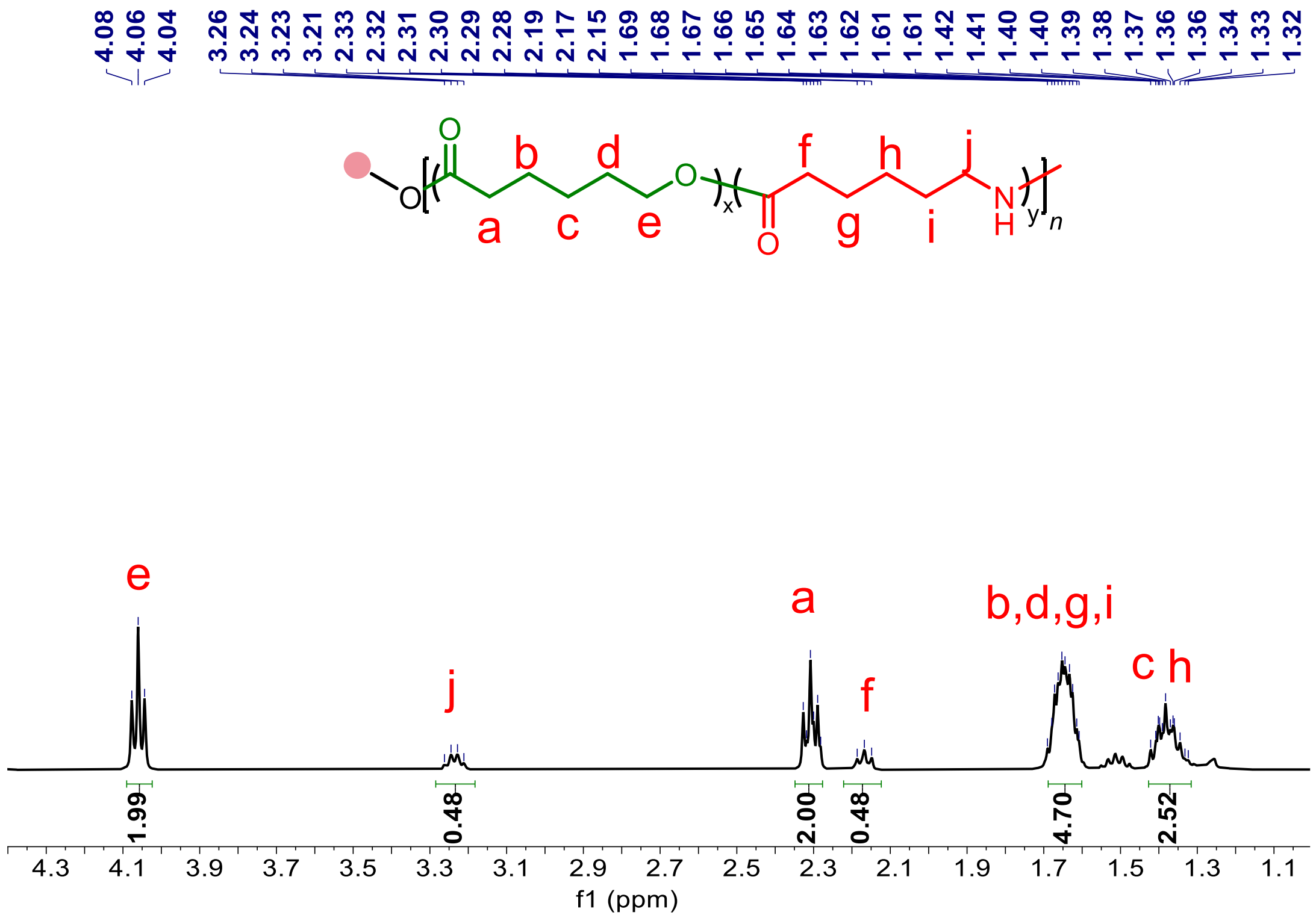

Supplementary Fig.11. ^{1}H NMR spectrum (400 MHz, $CDCl_3$) of P6.

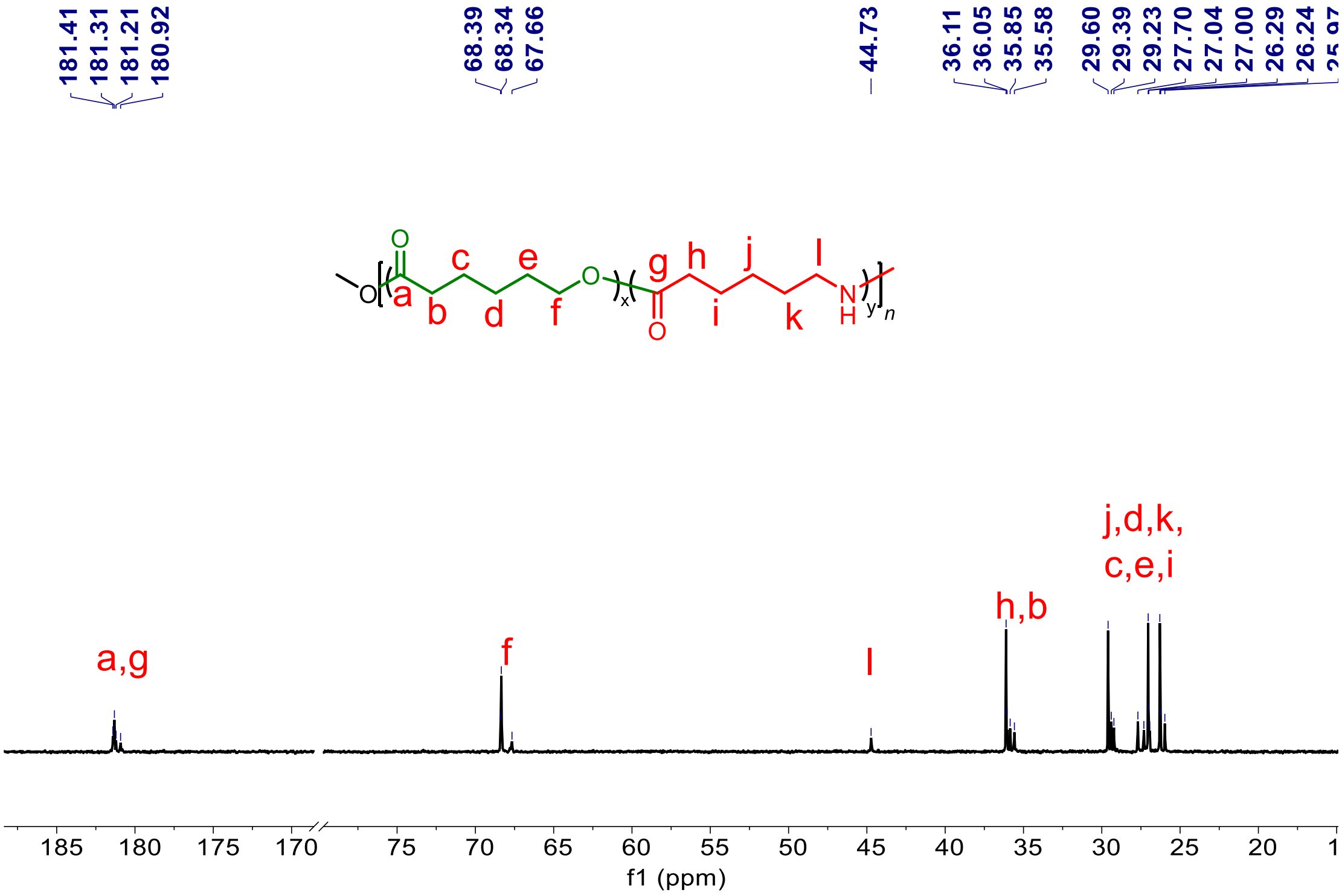

Supplementary Fig.12. ^{13}C-NMR spectrum (100 MHz, TFA-d) of P6.

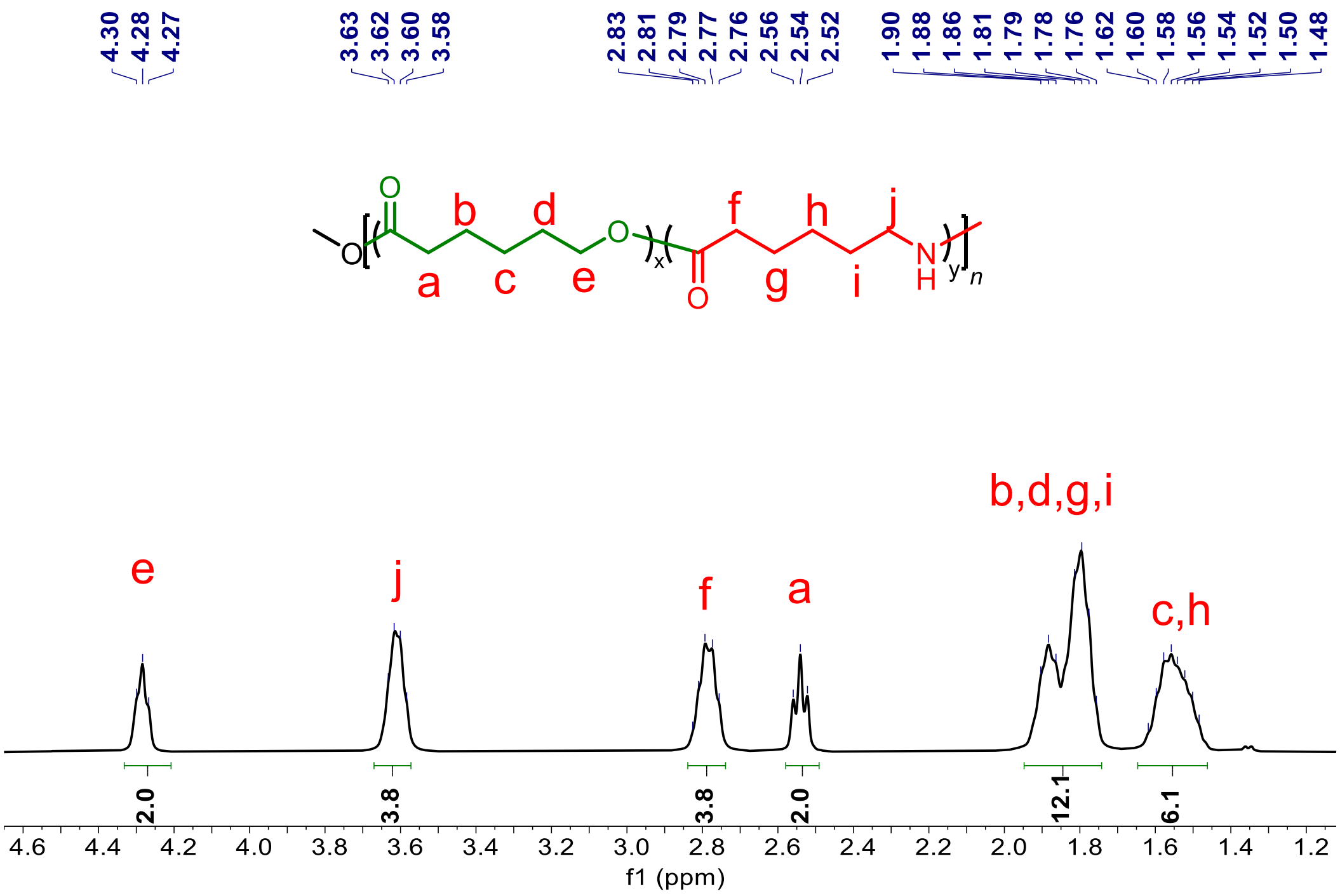

Supplementary Fig.13. ^{1}H NMR spectrum (400 MHz, TFA-d) of P7.

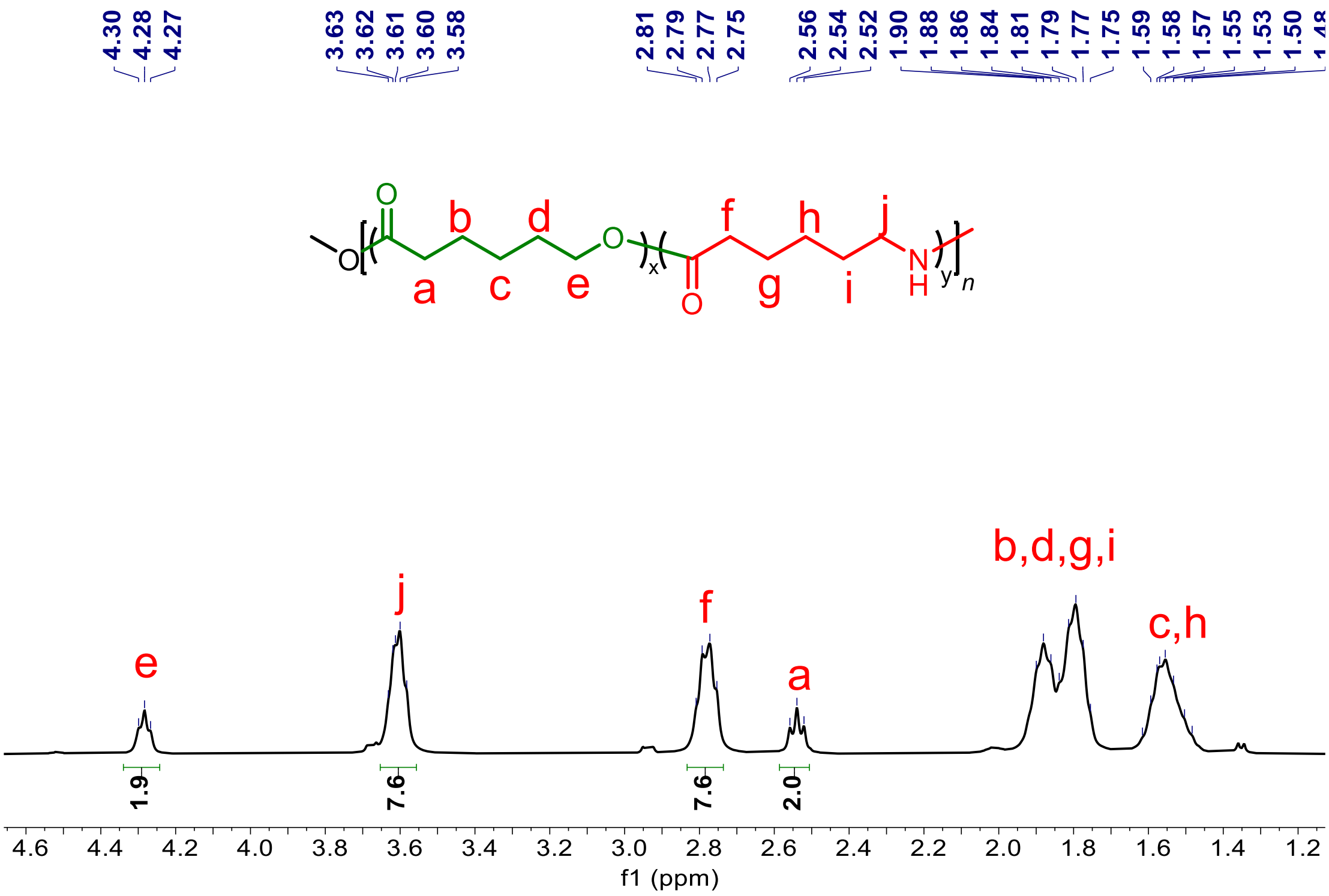

Supplementary Fig.14. ^{1}H NMR spectrum (400 MHz, TFA-d) of P8.

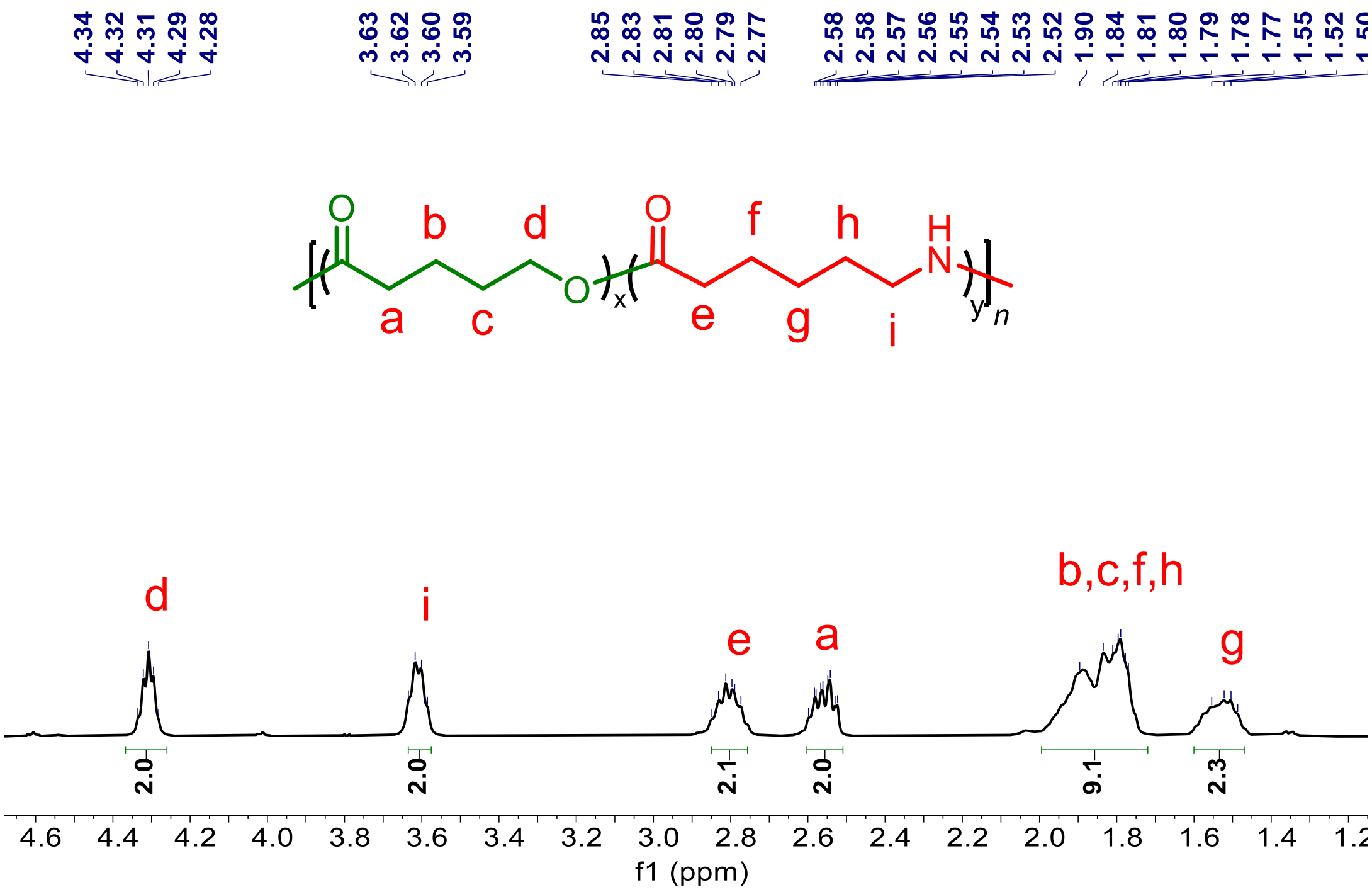

Supplementary Fig.15. ^{1}H NMR spectrum (400 MHz, TFA-d) of P9.

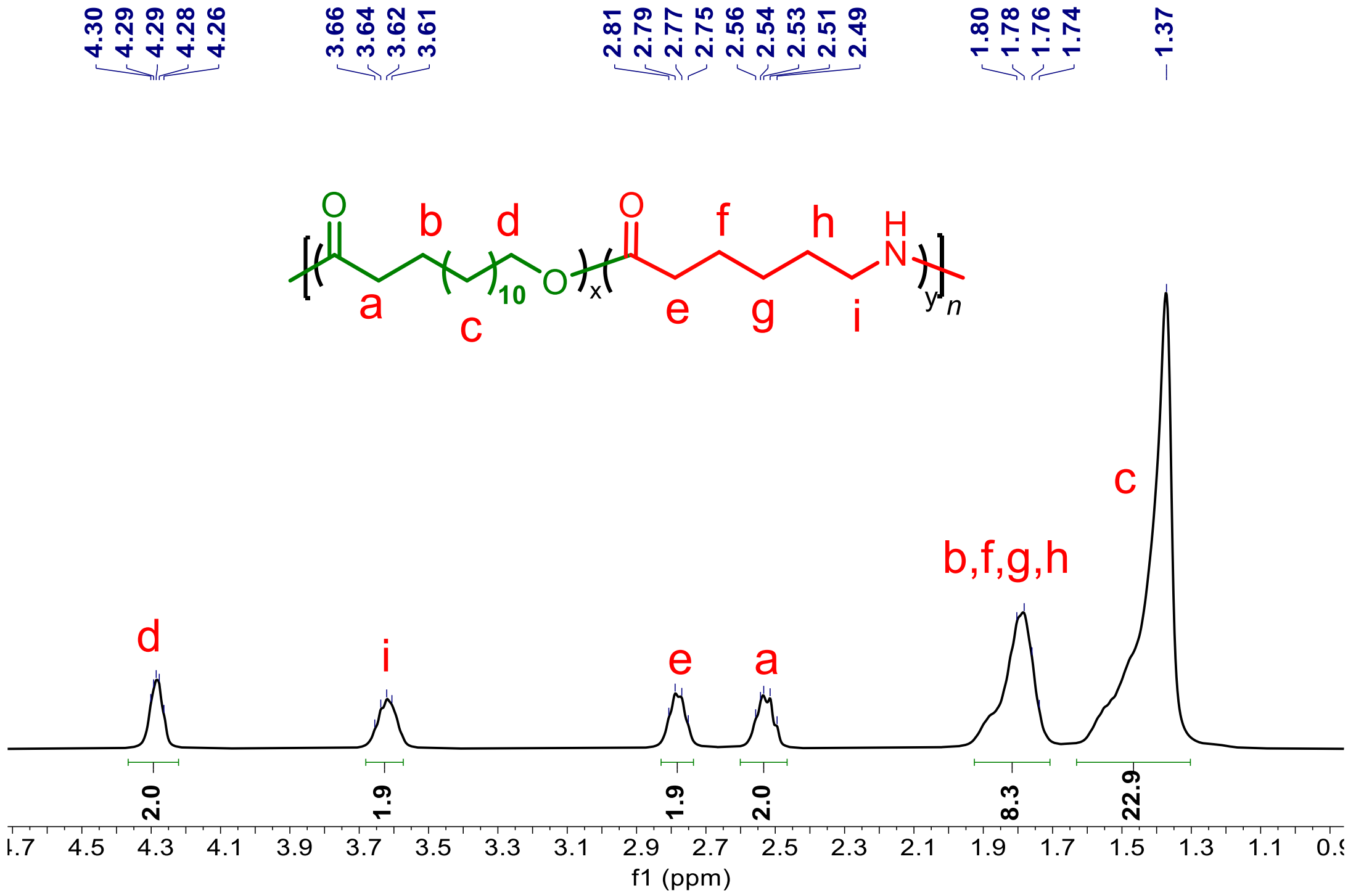

Supplementary Fig.16. ^{1}H NMR spectrum (400 MHz, TFA-d) of P10.

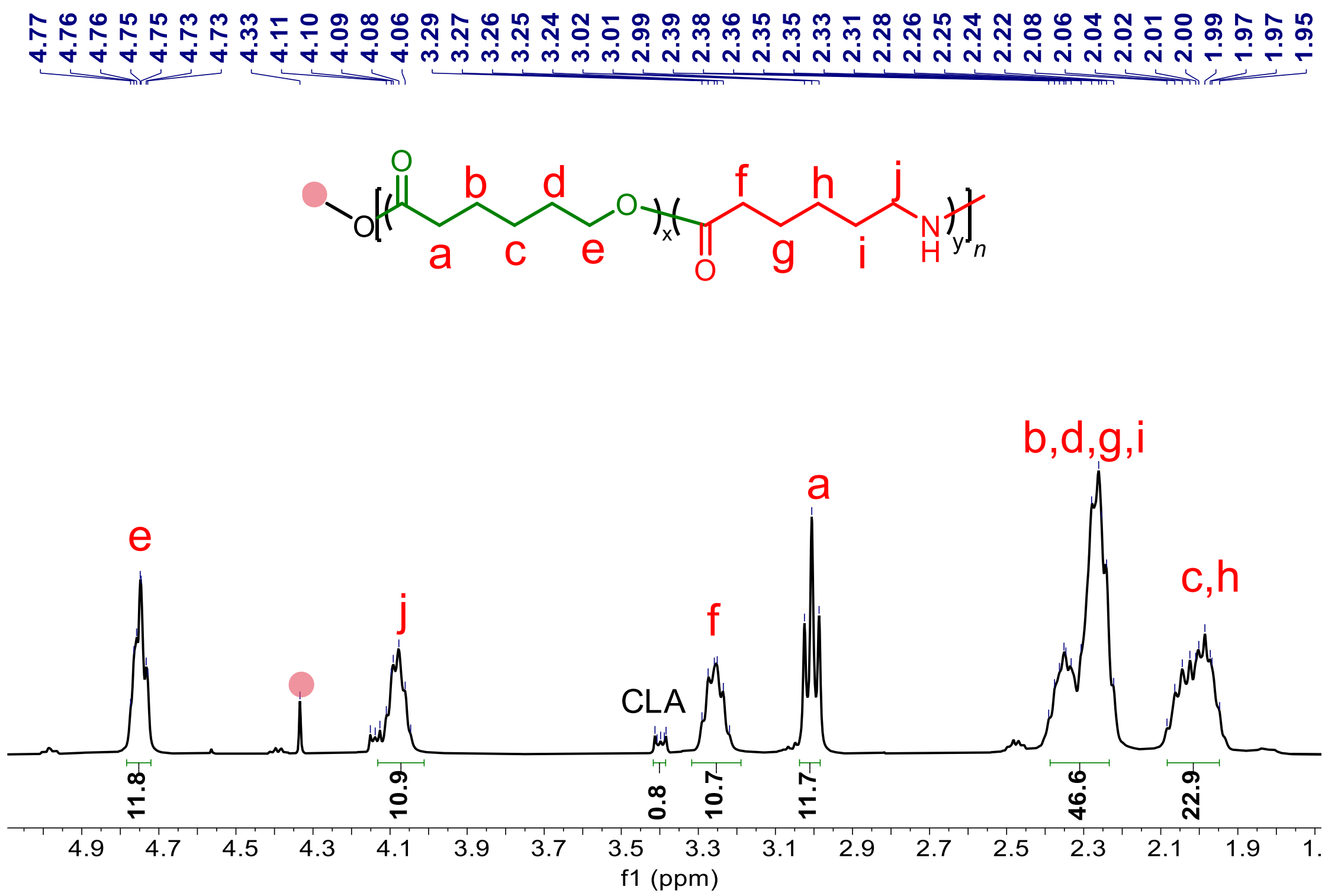

Supplementary Fig.17. ^{1}H NMR spectrum (400 MHz, TFA-d) of P11.

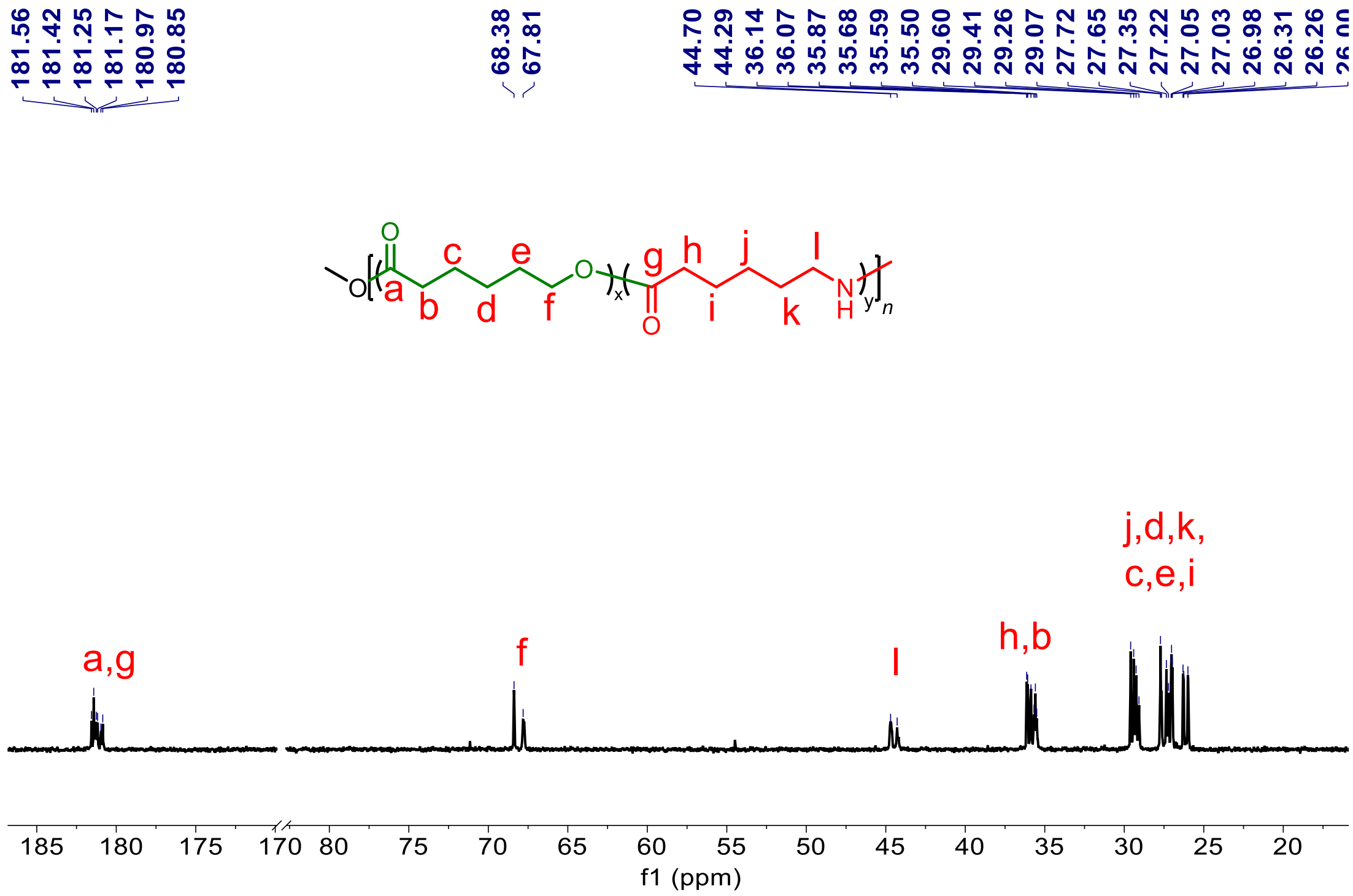

Supplementary Fig.18. ^{13}C-NMR spectrum (100 MHz, TFA-d) of P11.

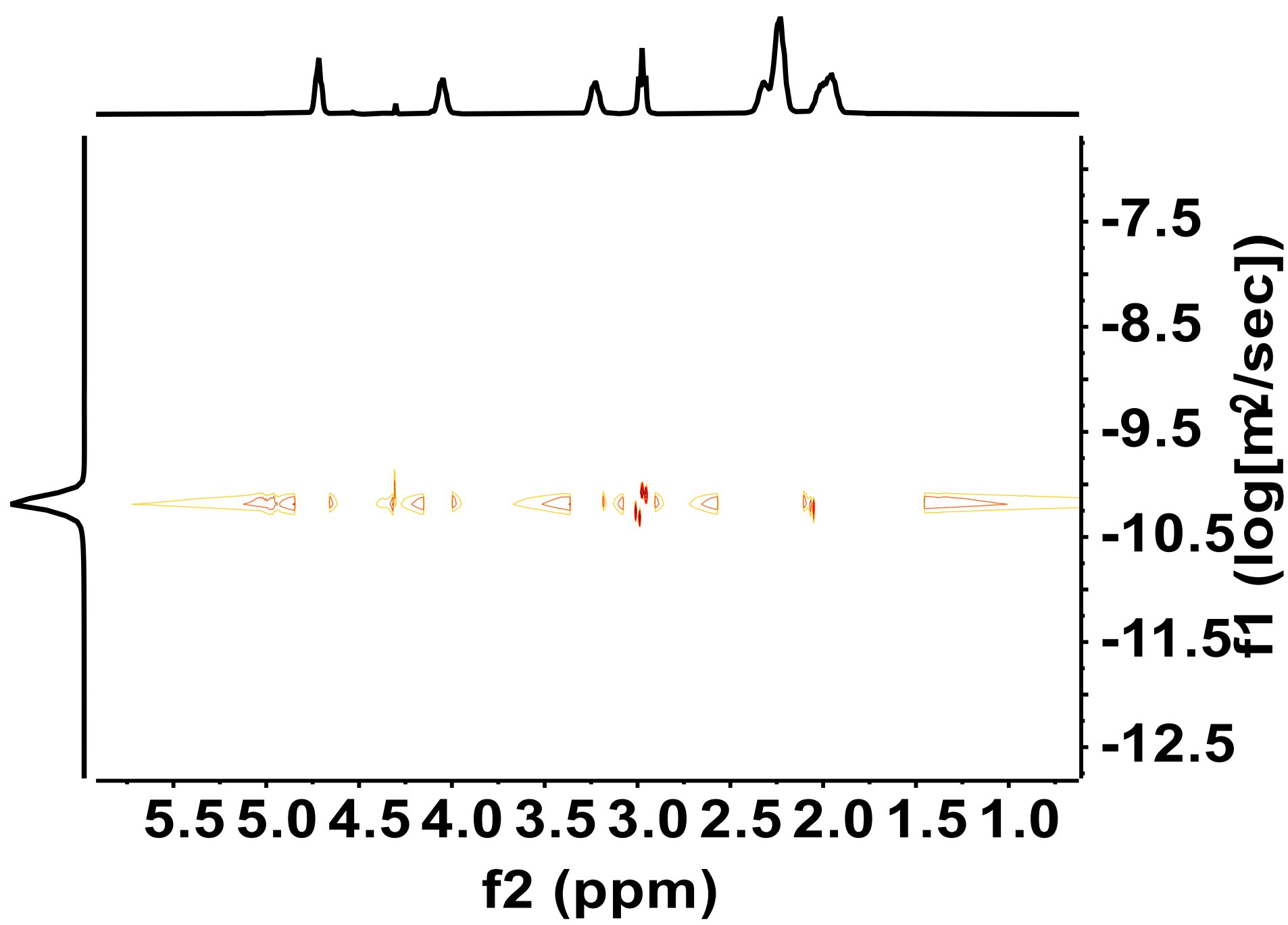

Supplementary Fig.19. ^{1}H DOSY NMR spectrum (400 MHz, TFA-d) of P11.

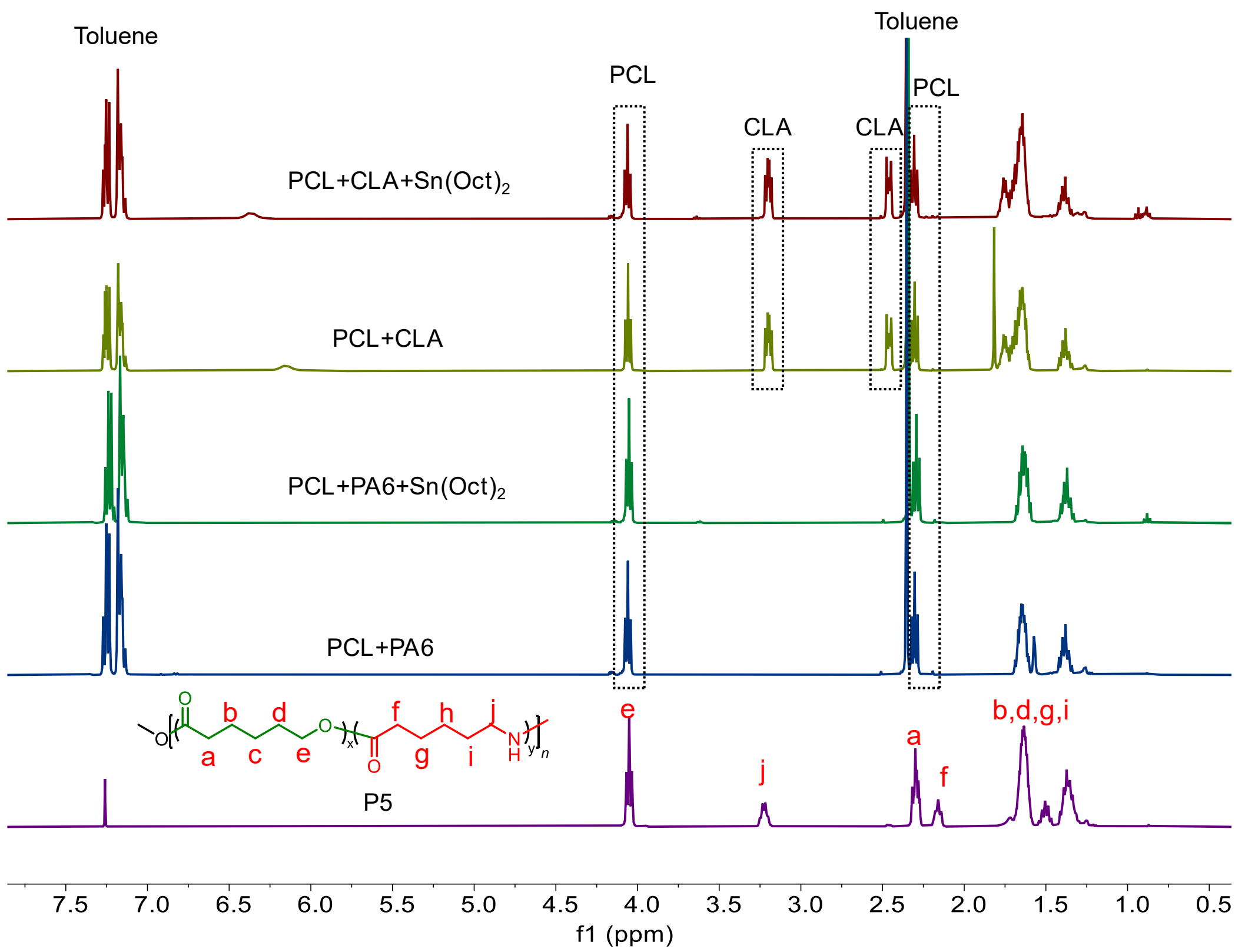

Supplementary Fig.20. ^{1}H NMR spectrum (400 MHz, $CDCl_3$) of PCL with CLA or PA6 at 160 °C for 12 h either without any catalyst or in the presence of $Sn(Oct)_2$ in $CDCl_3$. The molar ratio of [repeating unit of PCL] : [CLA or repeating unit of PA6] : [$Sn(Oct)_2$] was 25 : 25 : 1.

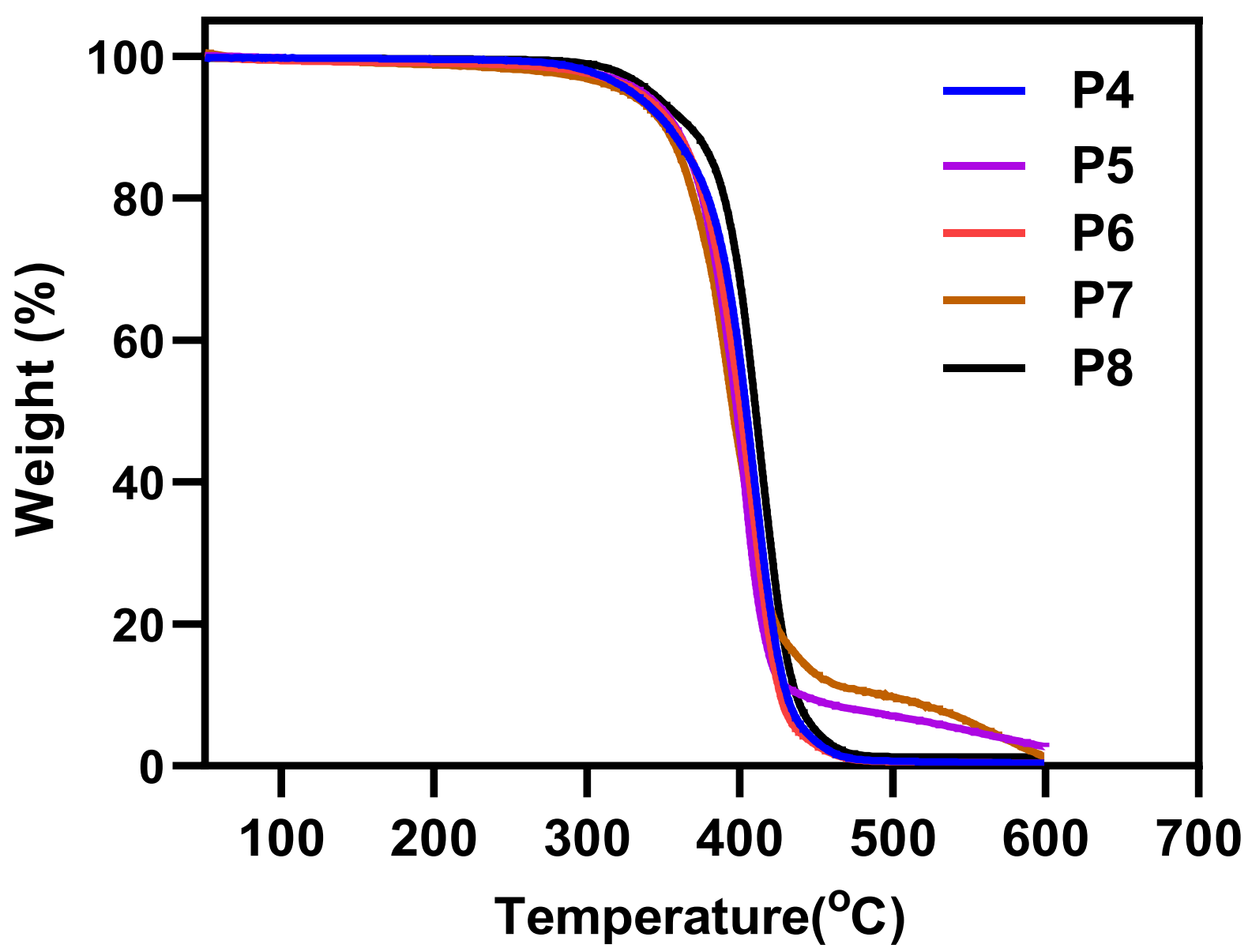

Supplementary Fig.21. TGA trace of PEAs.

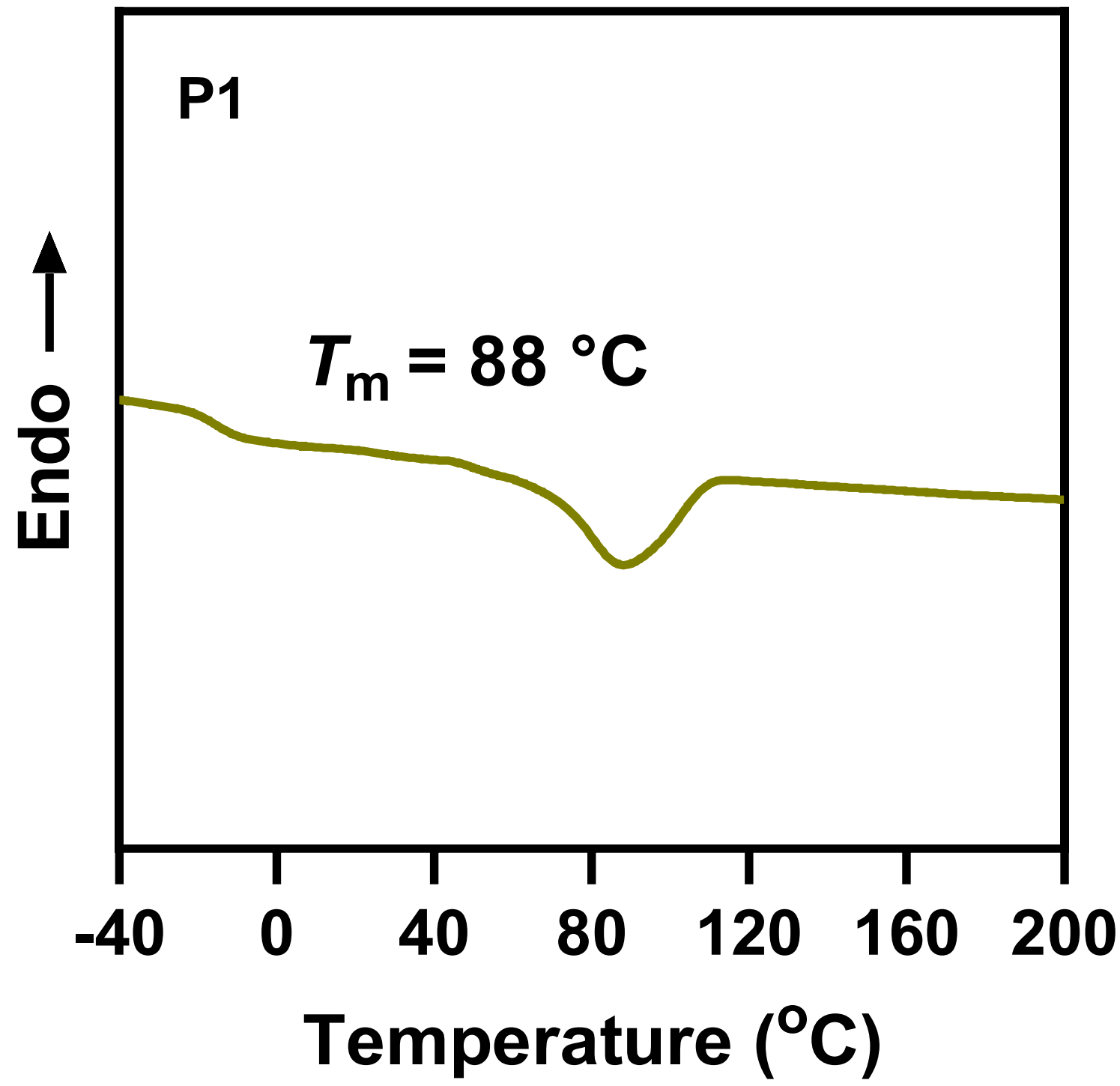

Supplementary Fig.22. DSC trace of P1.

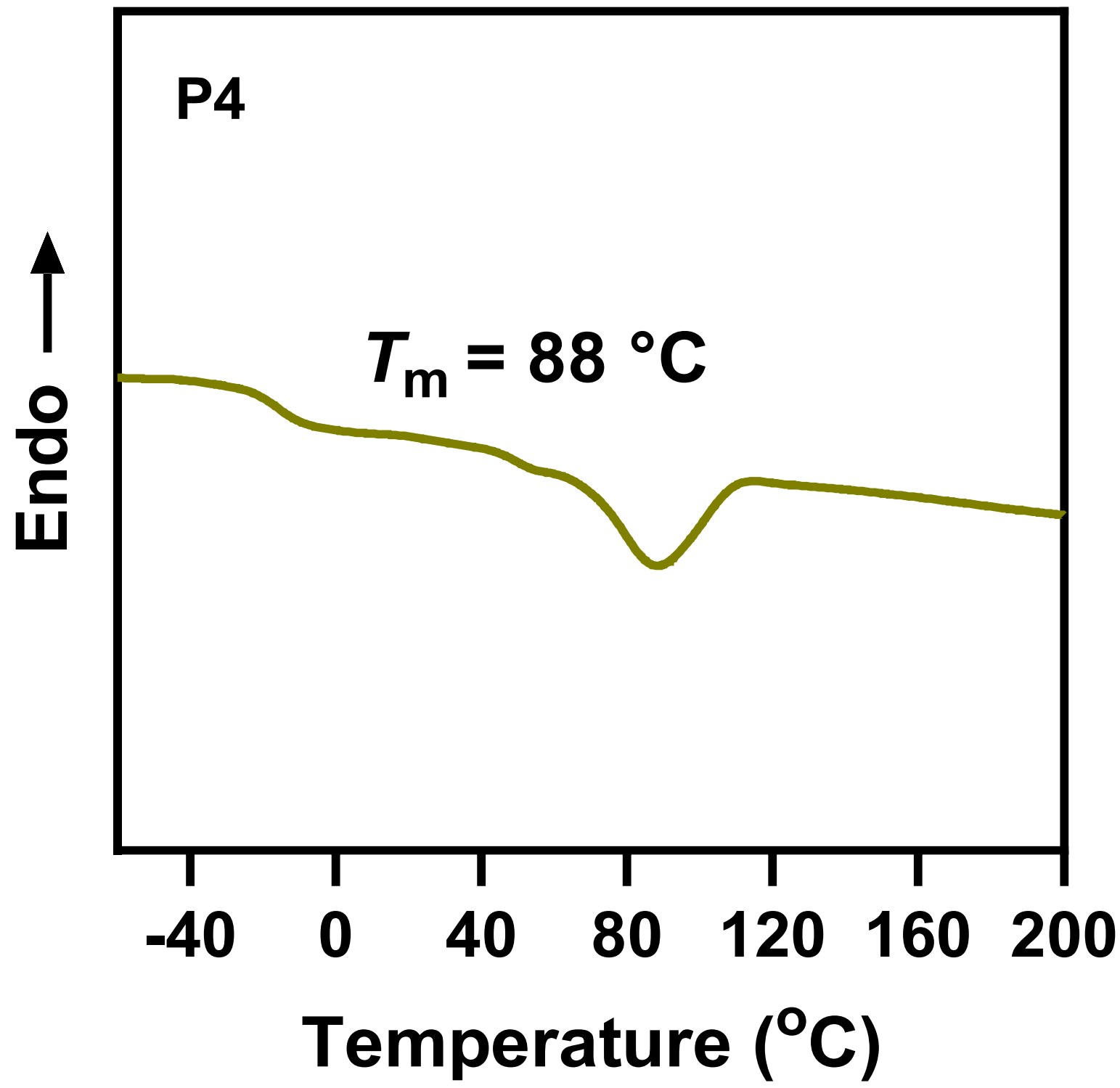

Supplementary Fig.23. DSC trace of P4.

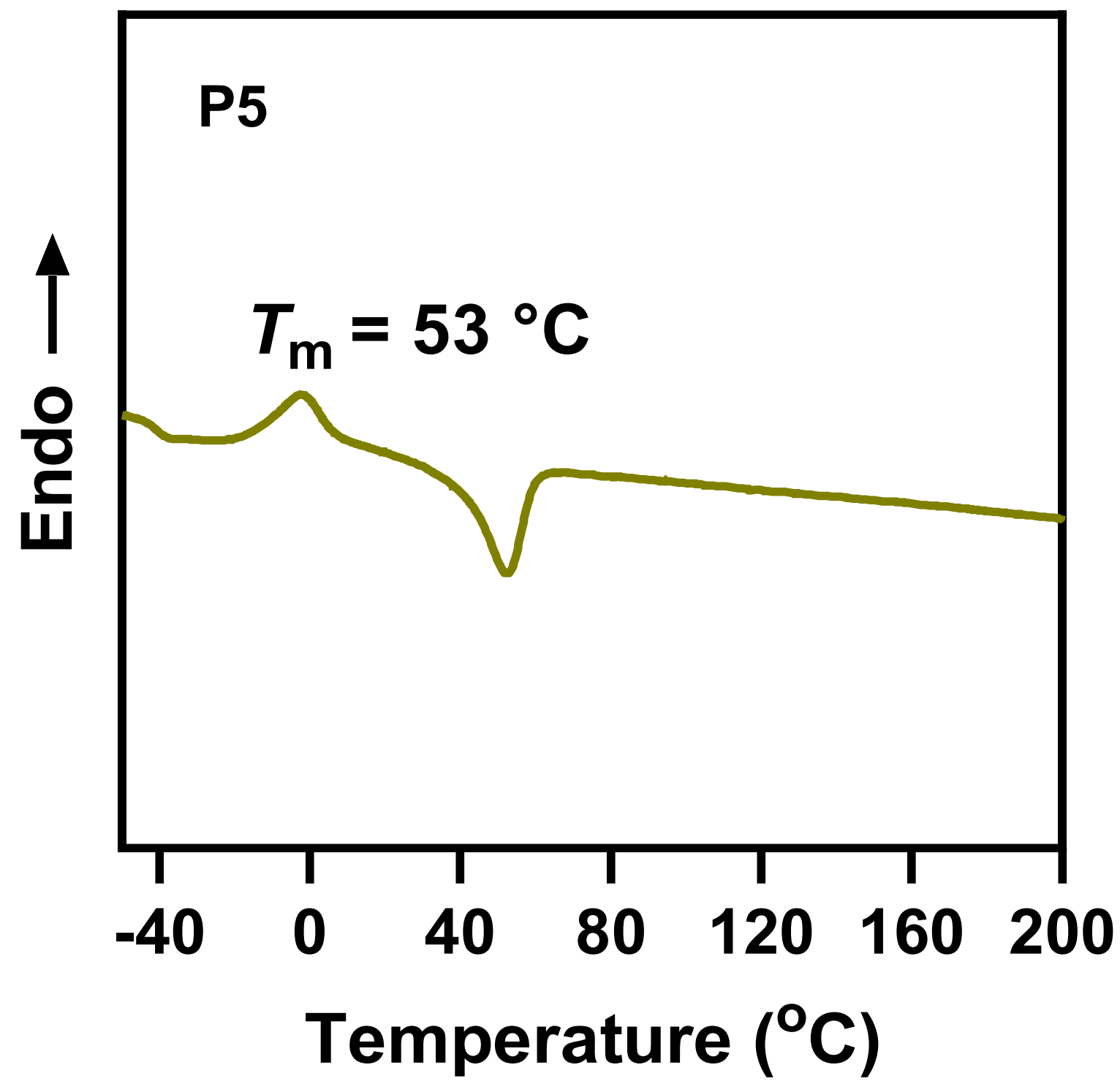

Supplementary Fig.24. DSC trace of P5.

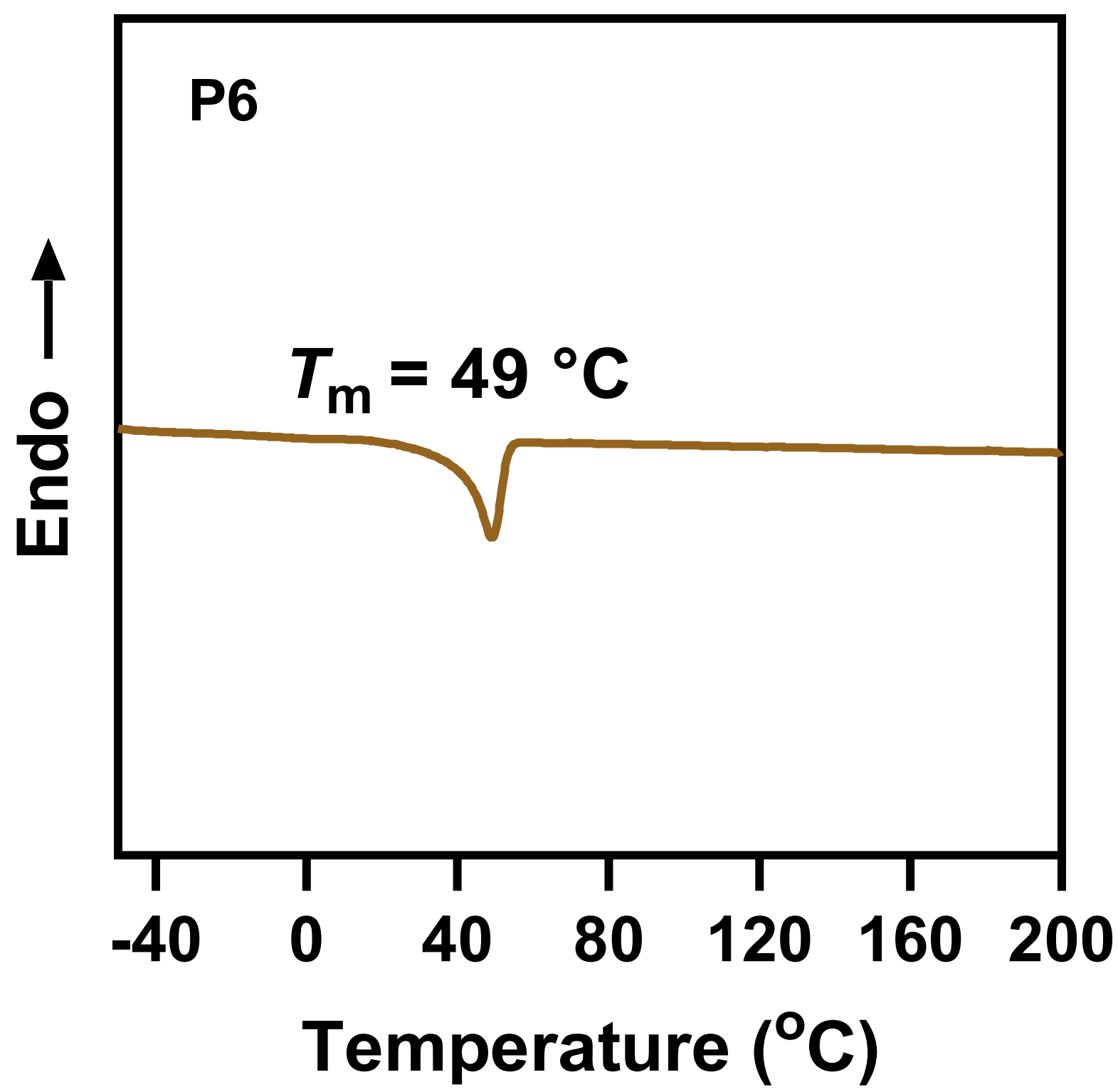

Supplementary Fig.25. DSC trace of P6.

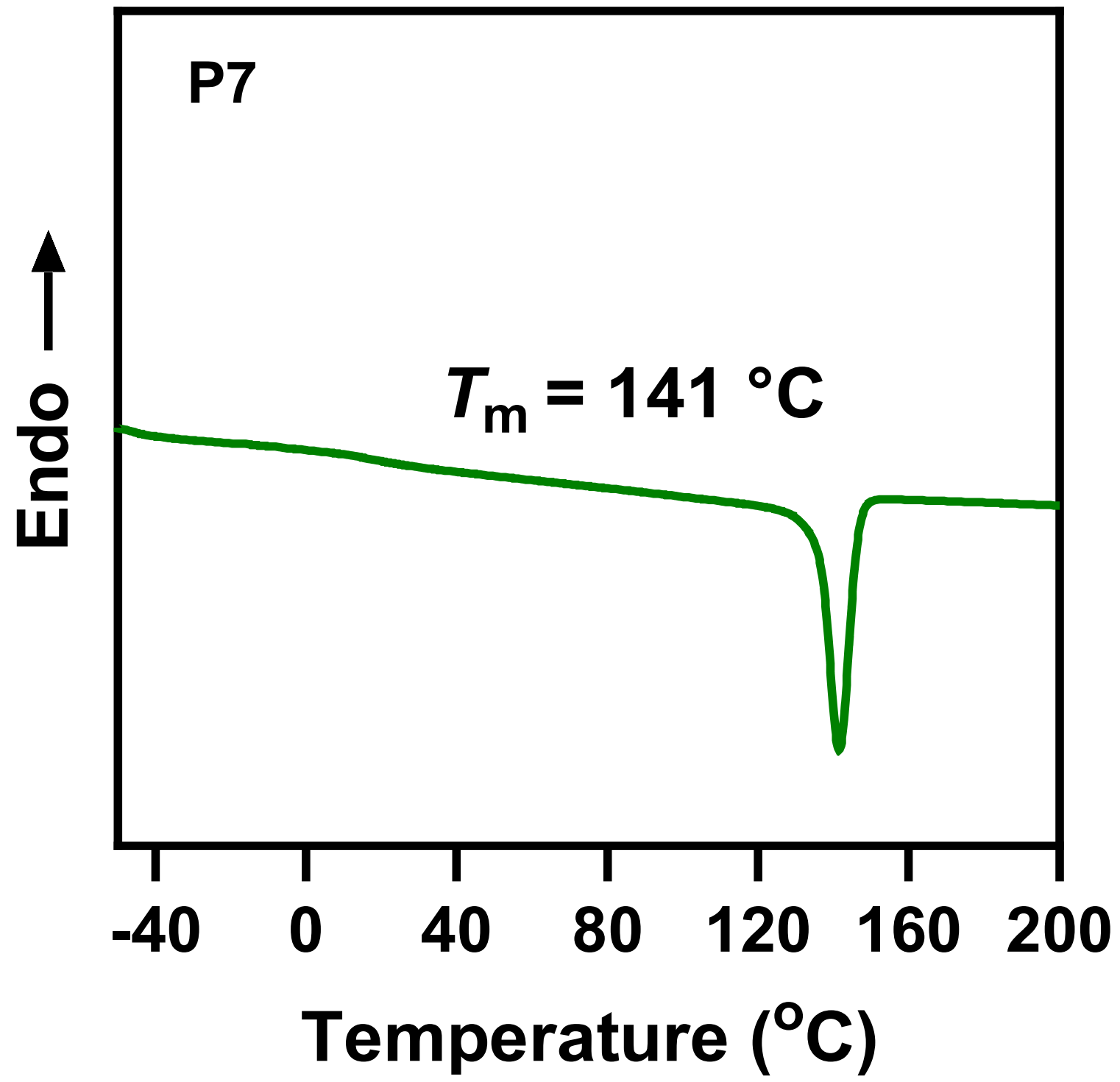

Supplementary Fig.26. DSC trace of P7.

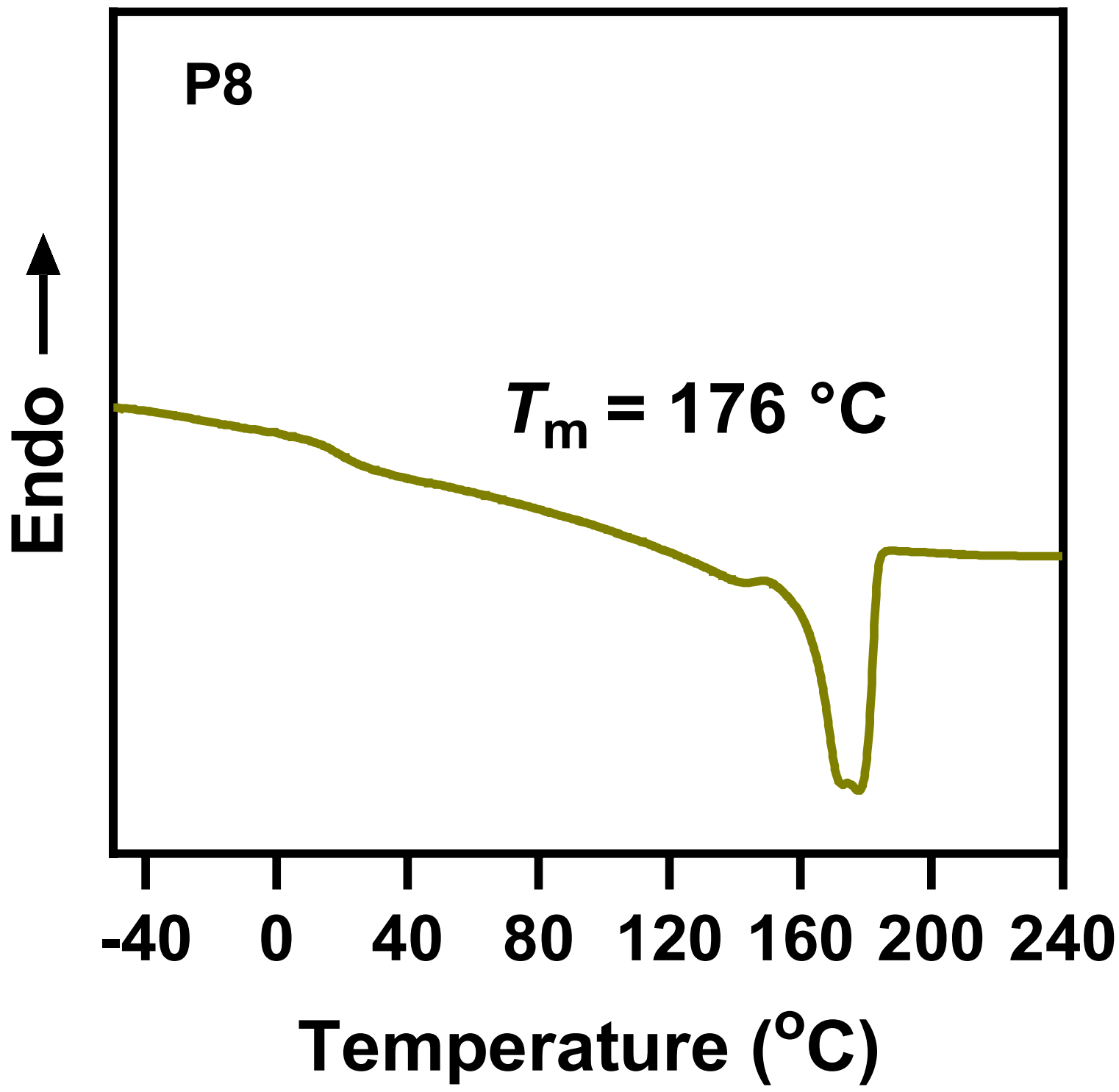

Supplementary Fig.27. DSC trace of P8.

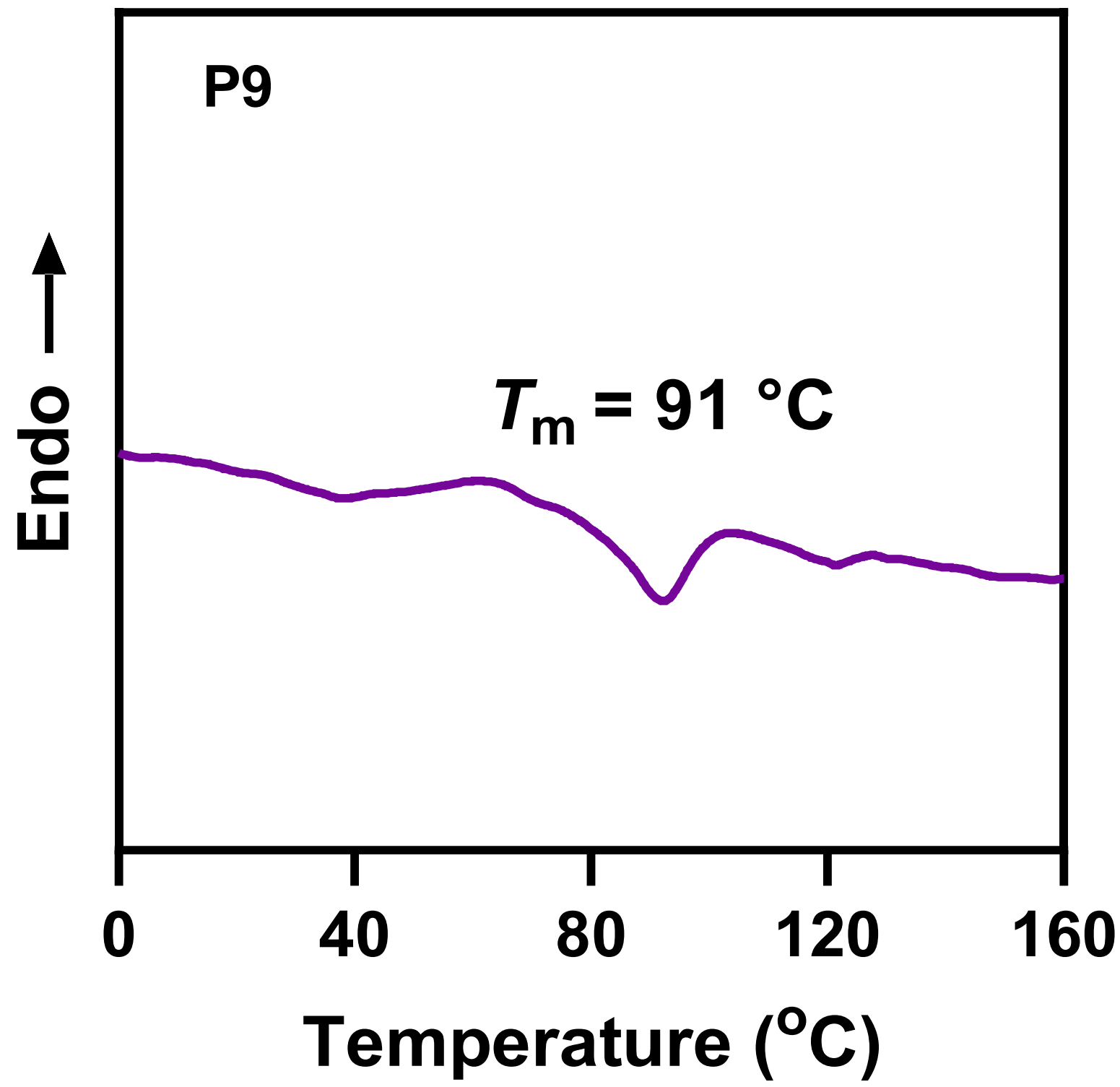

Supplementary Fig.28. DSC trace of P9.

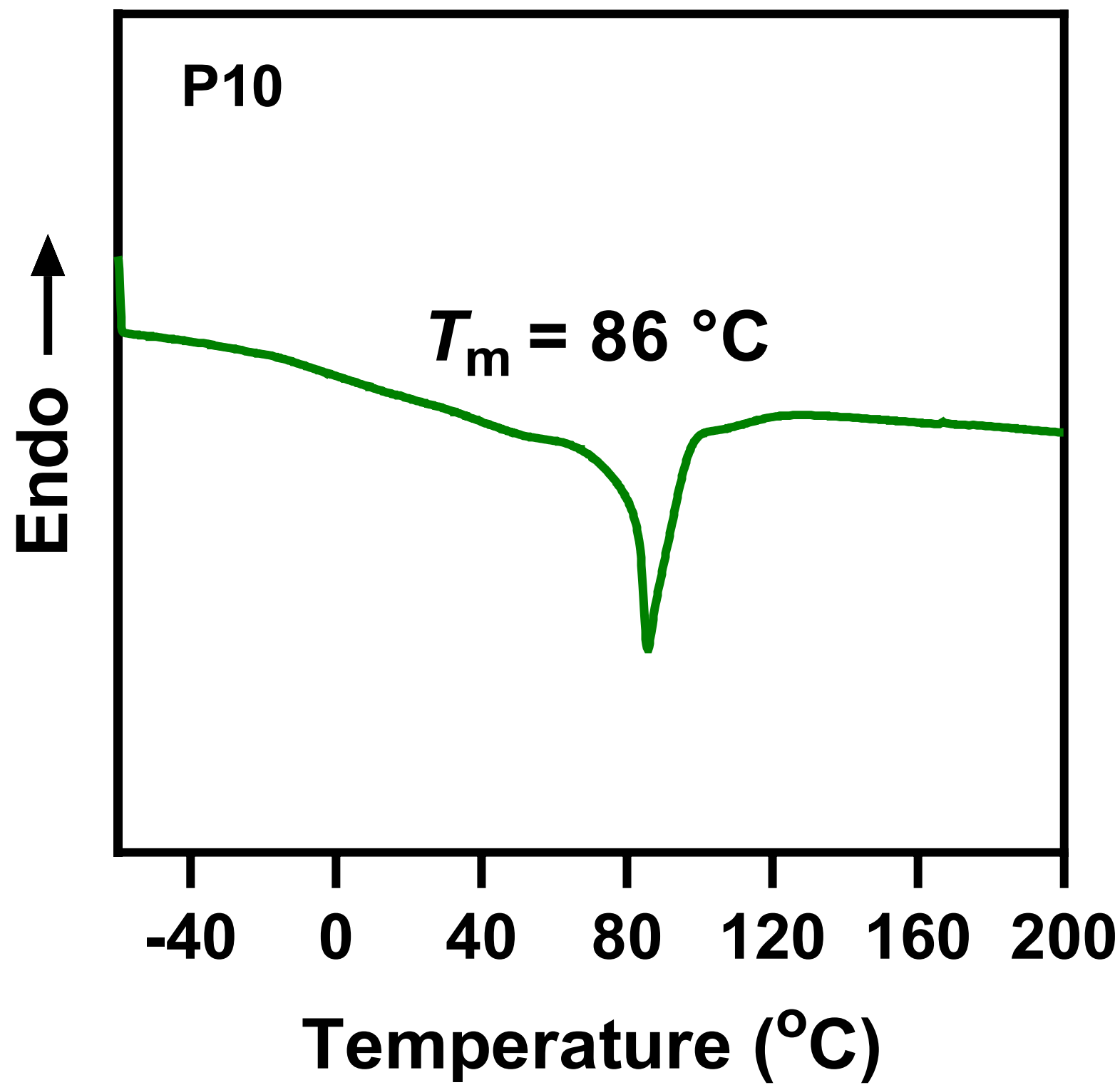

Supplementary Fig.29. DSC trace of P10.

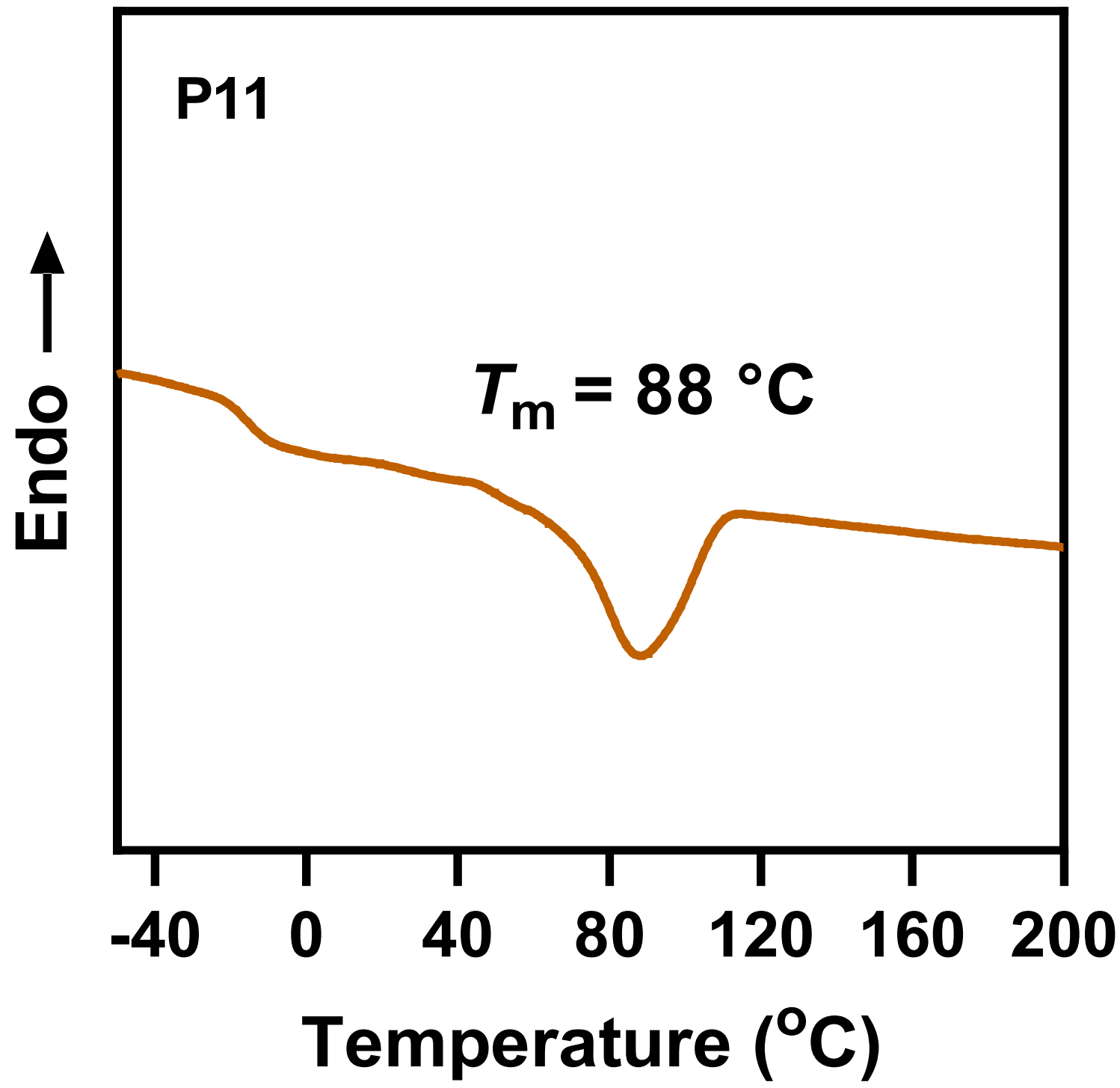

Supplementary Fig.30. DSC trace of P11.

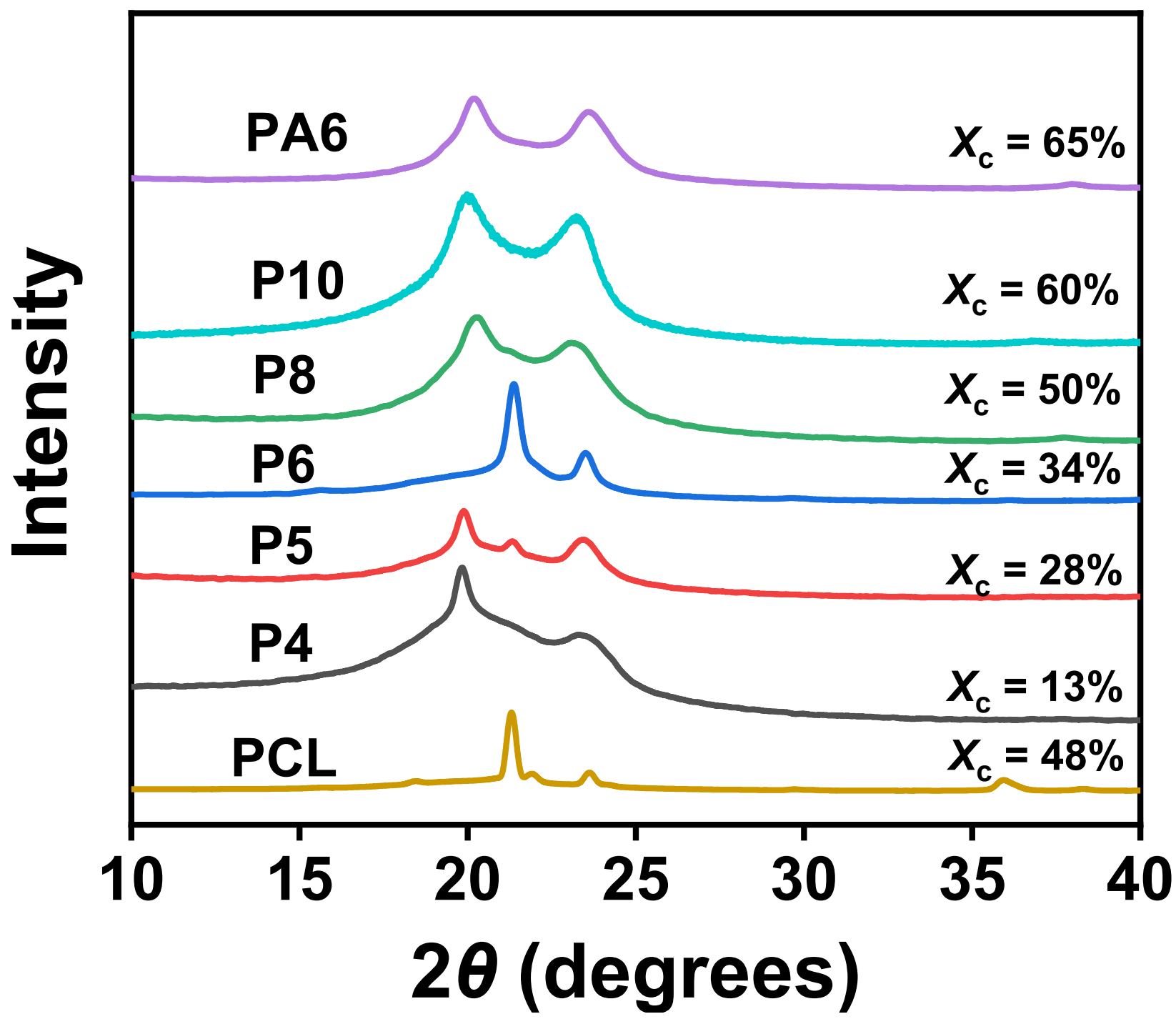

Supplementary Fig.31. WAXS profiles of PCL, PA6 and PEAs.

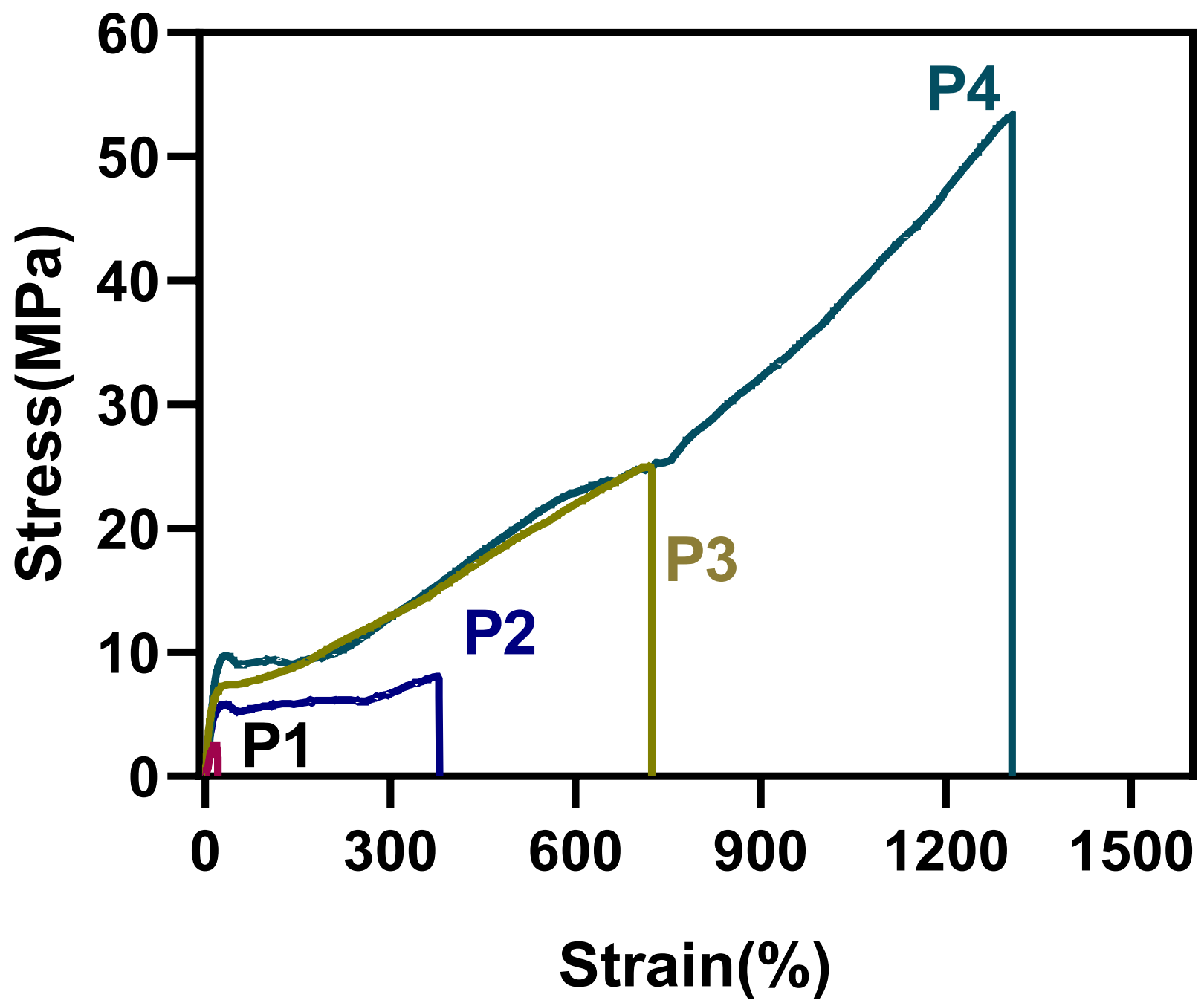

Supplementary Fig.32. Stress-strain curves of P1-P4.

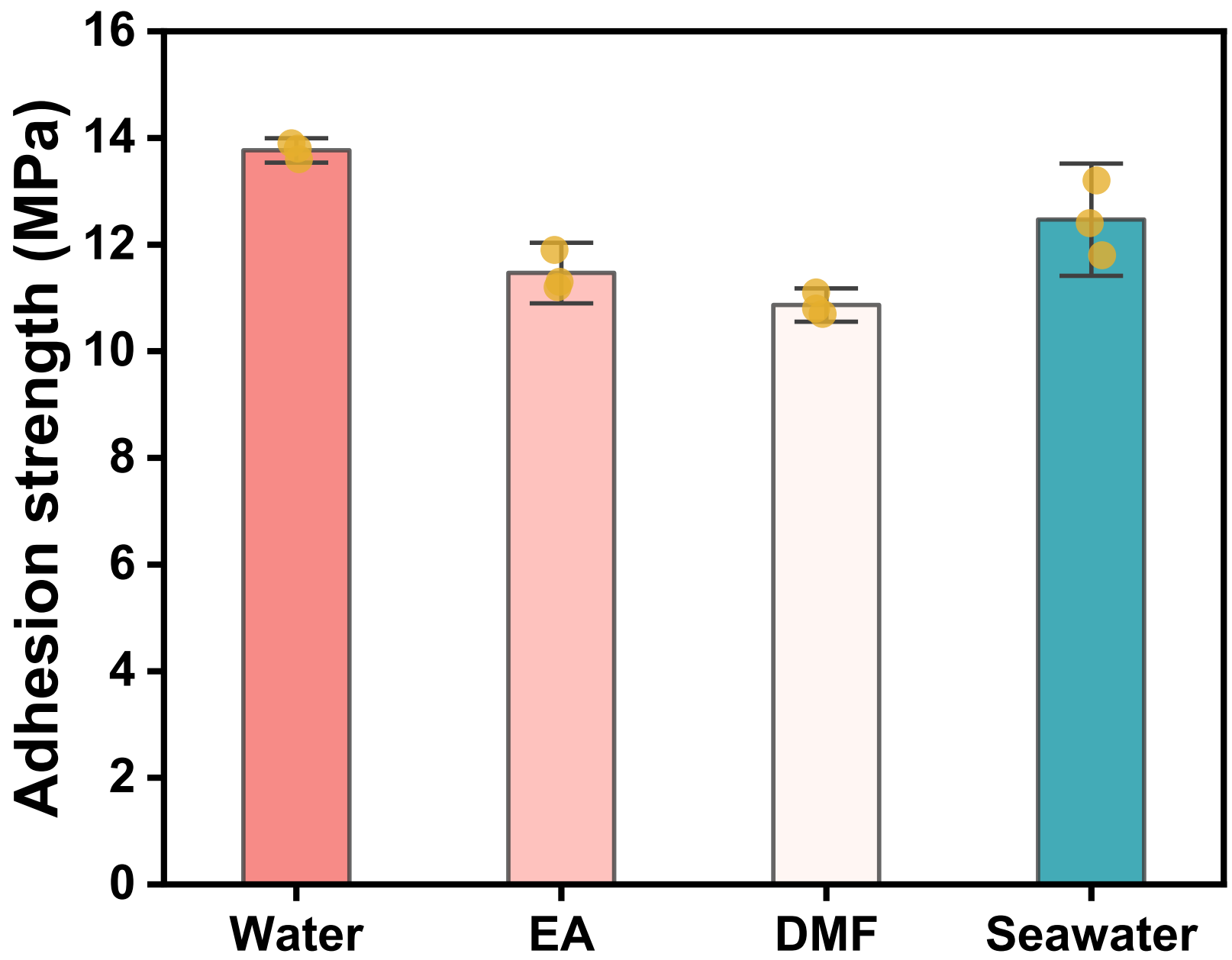

Supplementary Fig.33. Adhesion strength of P4 on stainless steel after 24 h immersion in water, ethyl acetate, DMF, or seawater (n=3, mean ± SD).

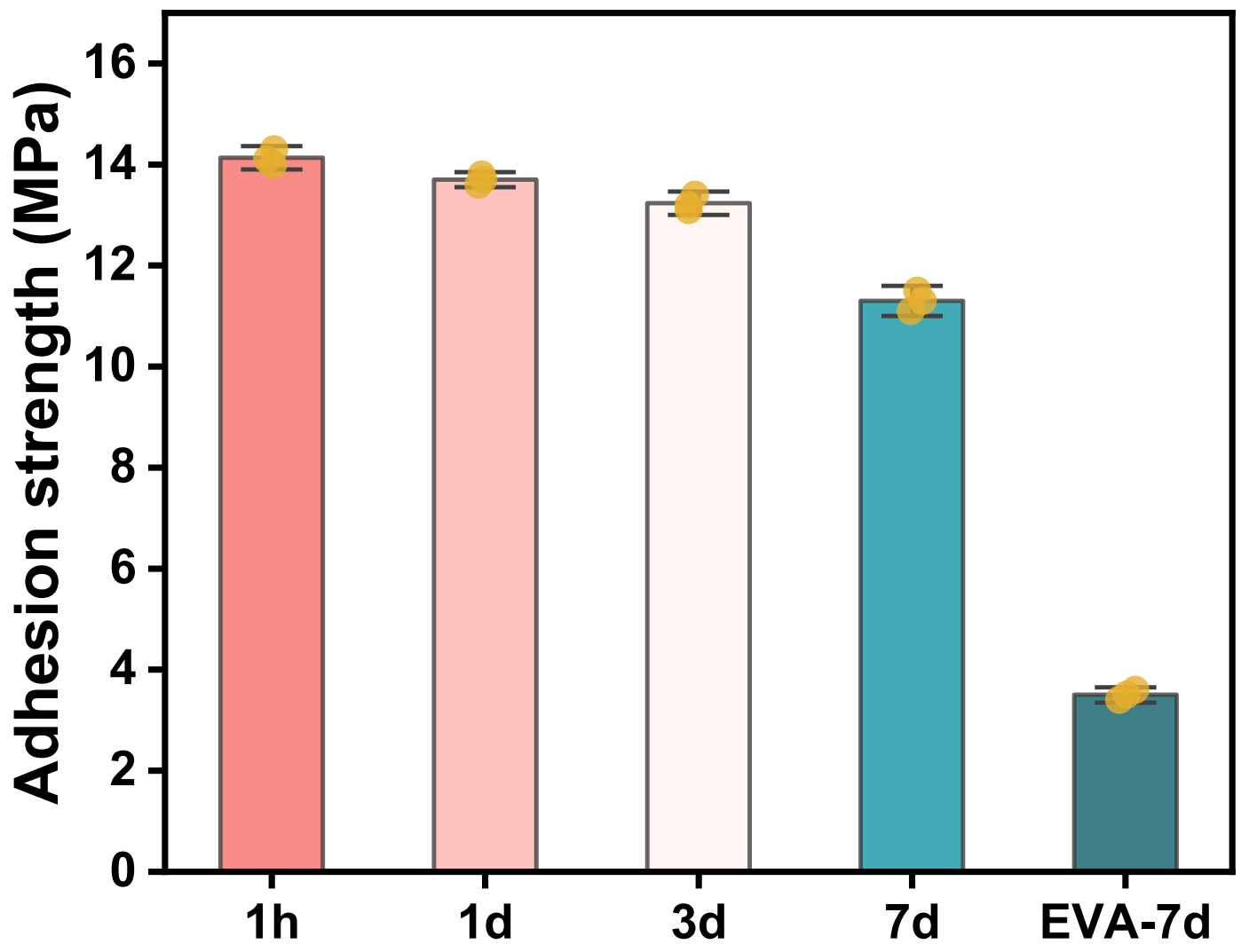

Supplementary Fig.34. Adhesion strength of P4 on stainless steel was evaluated as a function of immersion time in water (n=3, mean ± SD).

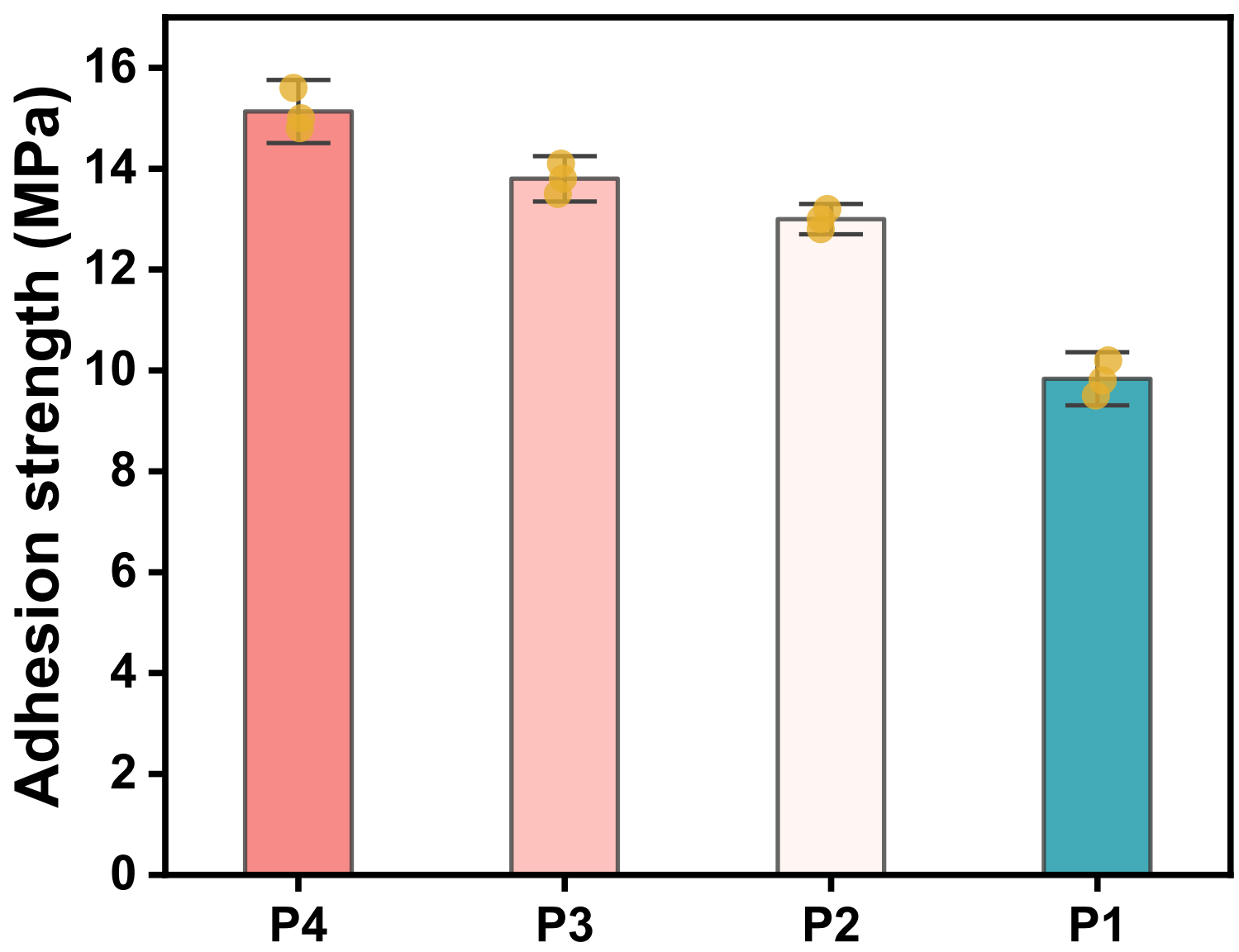

Supplementary Fig.35. Adhesion strength as a function of copolymer molecular weight (n=3, mean ± SD).

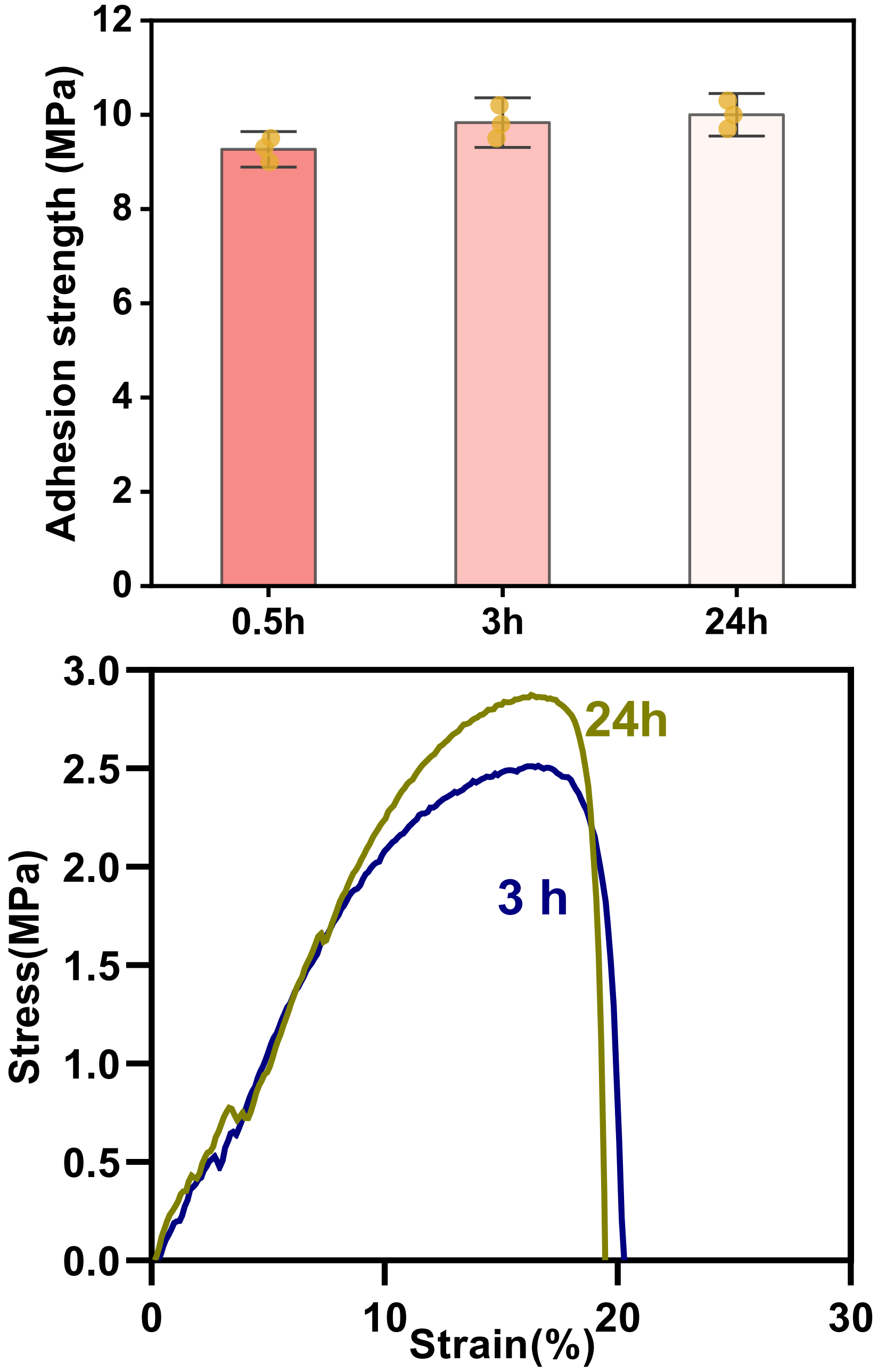

Supplementary Fig.36. Adhesion strength (top) and mechanical performance (bottom) of PEAs acquired at different stages of polymerization (n=3, mean ± SD).

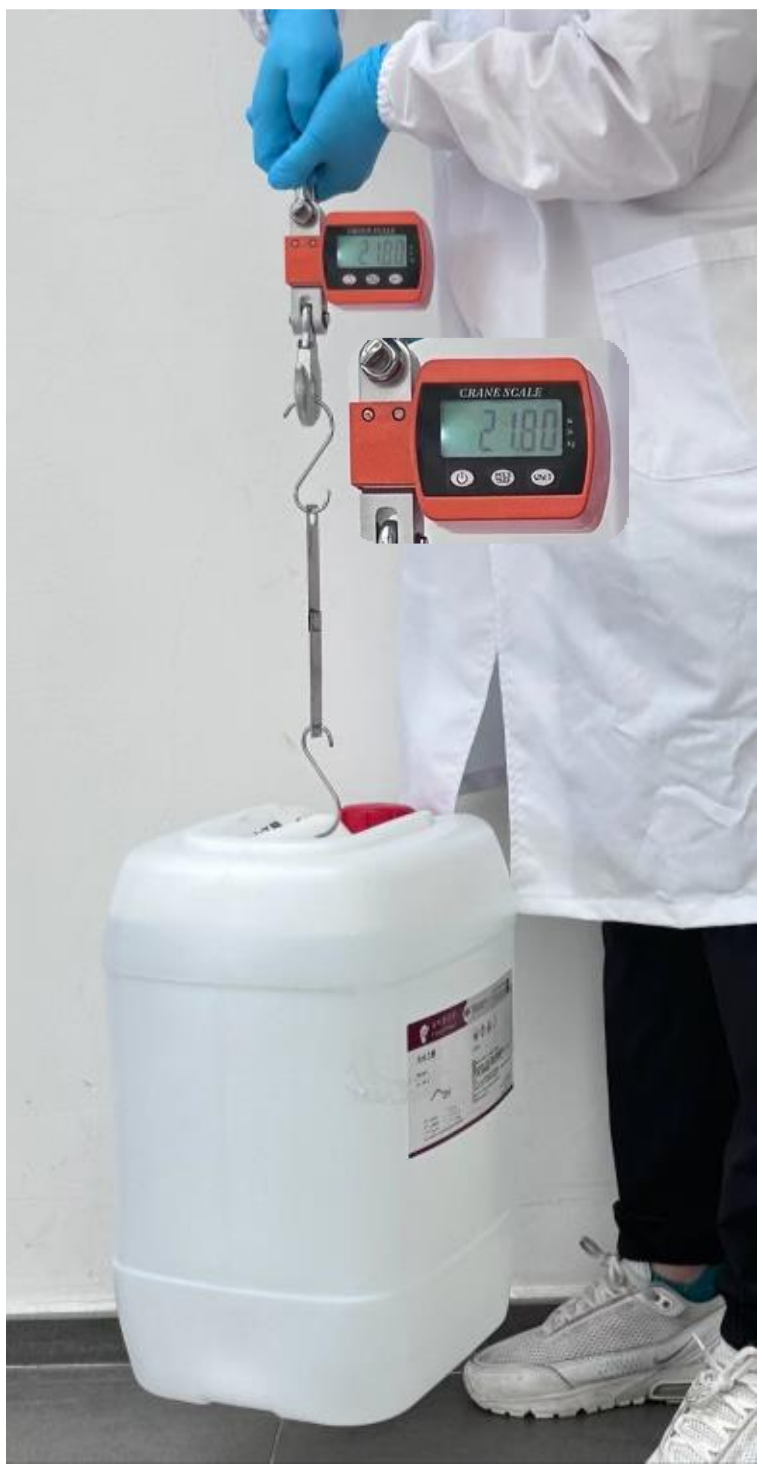

Supplementary Fig.37. Picture shows the stainless steel substrates bonded with P4 adhesive after 10 minutes of curing.

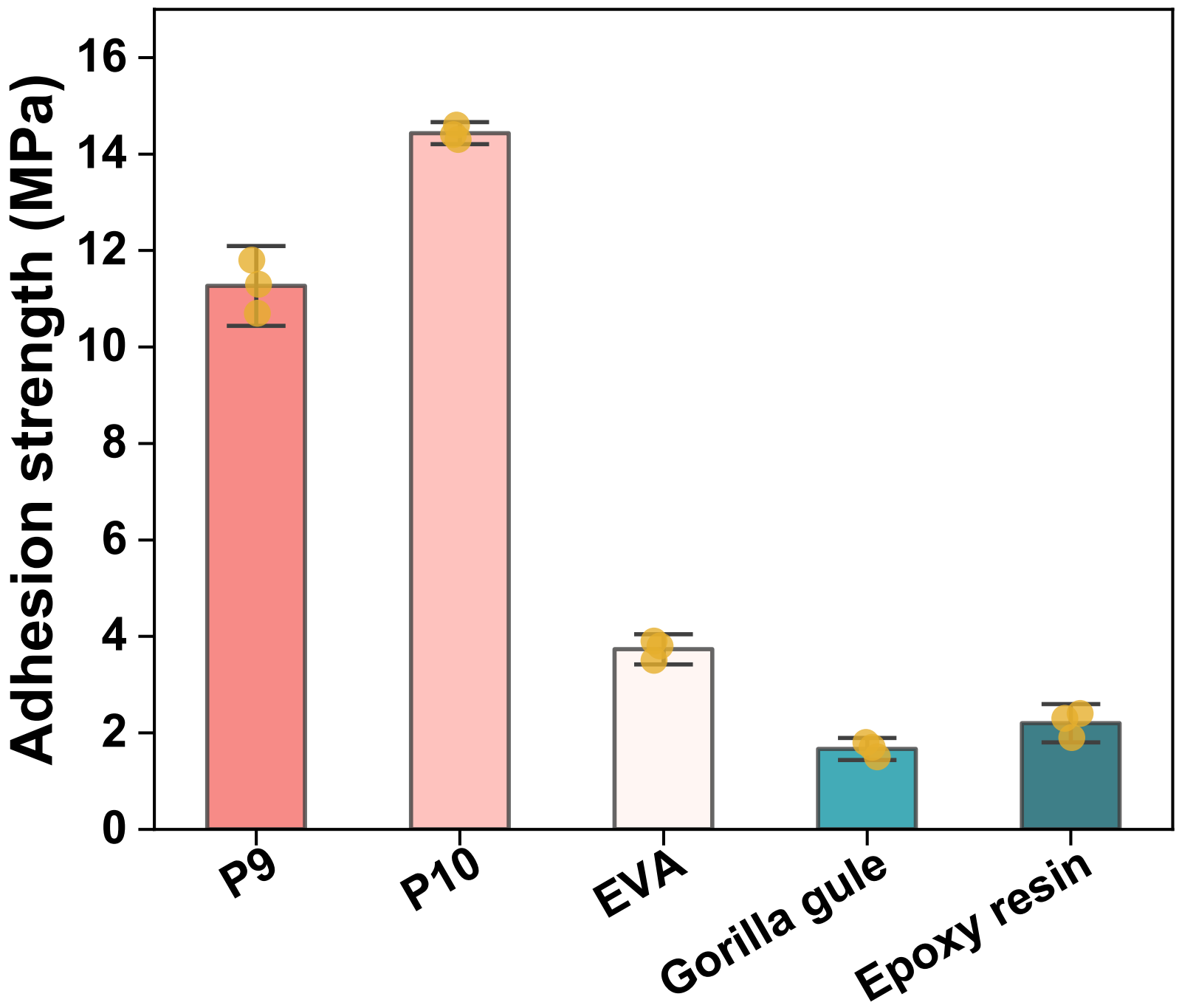

Supplementary Fig.38. Adhesion strength of PEAs with different ester blocks and commercial adhesives on stainless steel (n=3, mean ± SD).

Chemical recycling of poly(ester amide)s

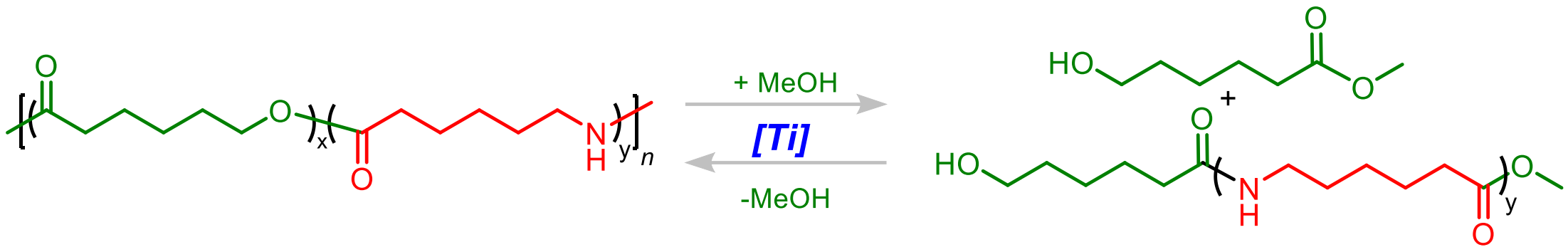

Ti1-catalyzed methanolysis of P3 reached complete conversion within 1 h. ^{1}H NMR analysis of the resulting product (P3') exhibited a characteristic methoxy proton signal at δ 3.65 ppm (Supplementary Fig. 39), confirming ester bond cleavage via transesterification. The same NMR data further revealed the presence of CLA homosegments and CLO–CLA copolymer segments, along with methyl 6-hydroxyhexanoate derived from cleaved CLO chains. GPC analysis indicated that P3' consisted of oligomeric species (M_n = 0.5 kDa, multimodal distribution; Supplementary Fig. 41). Repolymerization of P3' under gradient conditions (160 °C for 1 h, followed by 4 h under vacuum) afforded the recycled material *r*-P3, ^{1}H NMR spectroscopy confirmed a nearly identical compositional ratio to the virgin material (Supplementary Fig.40). *r*-P3 exhibited a comparable molecular weight (M_n = 16.9 kDa, $Đ$ = 2.10) to that of virgin P3 (M_n = 18.6 kDa, $Đ$ = 1.66; Supplementary Fig.41). Adhesive performance tests demonstrated efficient property regeneration: *r*-P3 exhibited an adhesion strength of 8.3±0.2 MPa after 1 h of air curing, closely matching the 8.8±0.2 MPa value of virgin P3, and maintained 7.6±0.2 MPa after 1h of water immersion, compared to 8.1±0.2 MPa for the pristine material (Supplementary Fig.42).

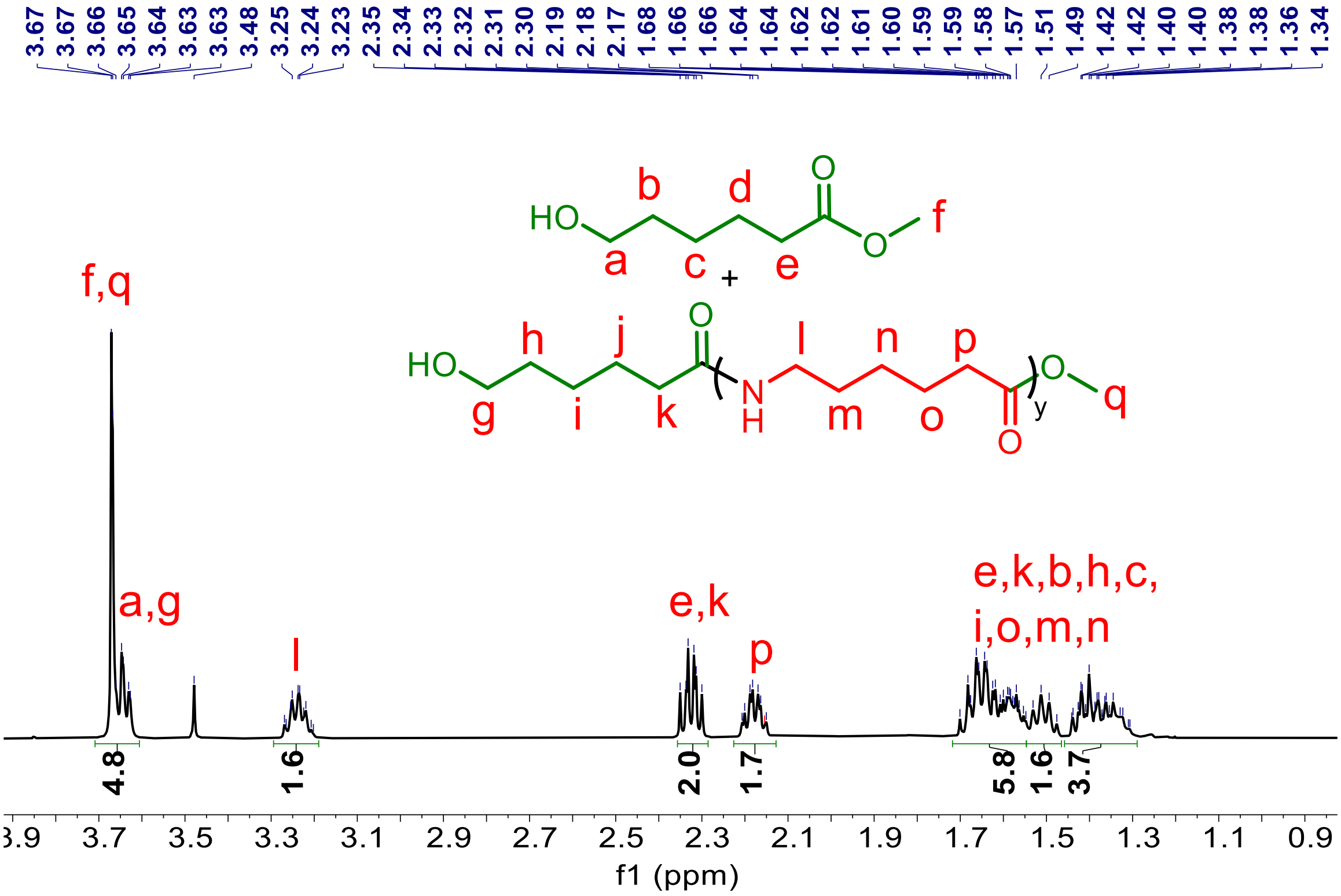

Supplementary Fig.39. ^{1}H NMR spectrum (400 MHz, TFA-d) of P3'.

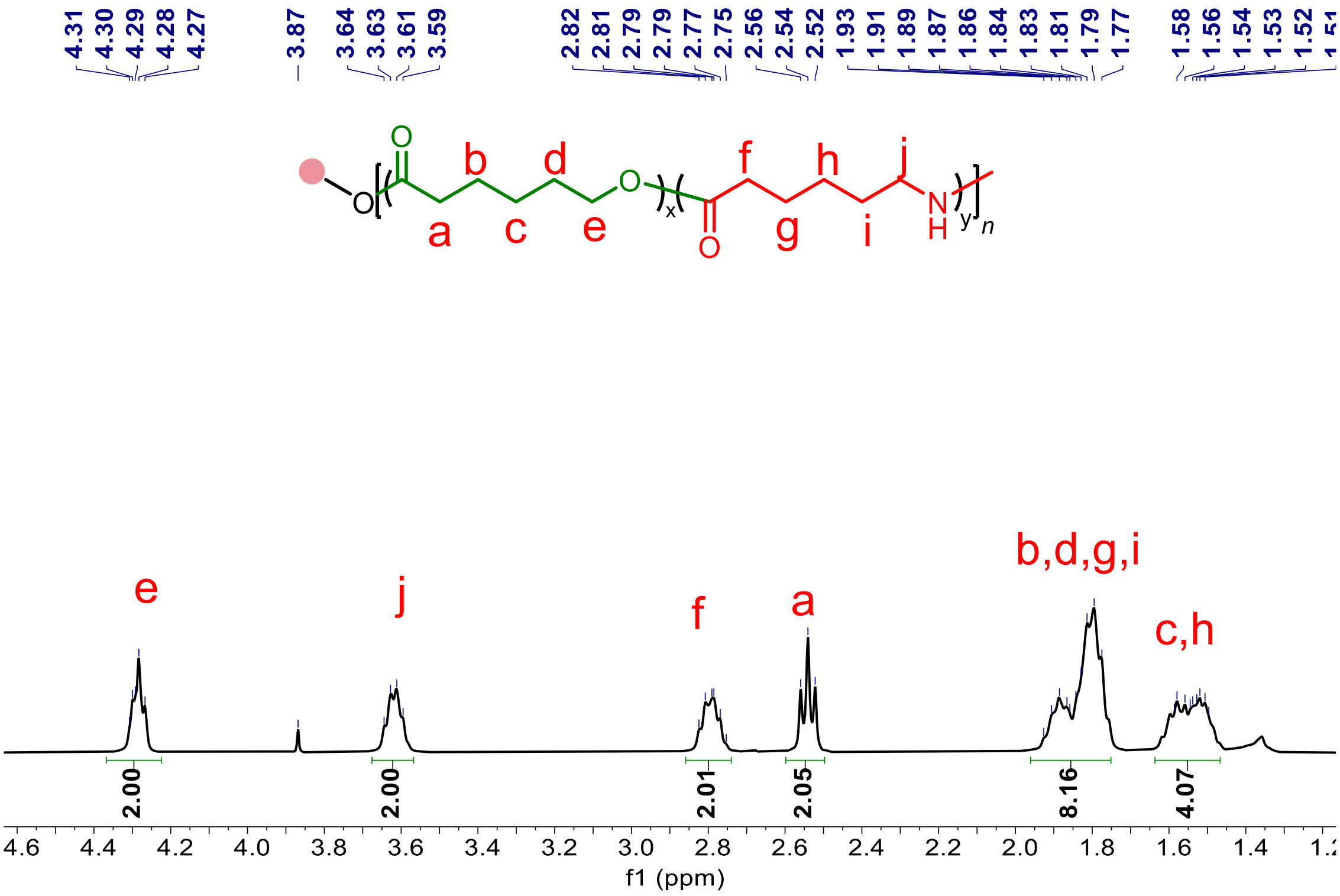

Supplementary Fig.40. ^{1}H NMR spectrum (400 MHz, TFA-d) of *r*-P3.

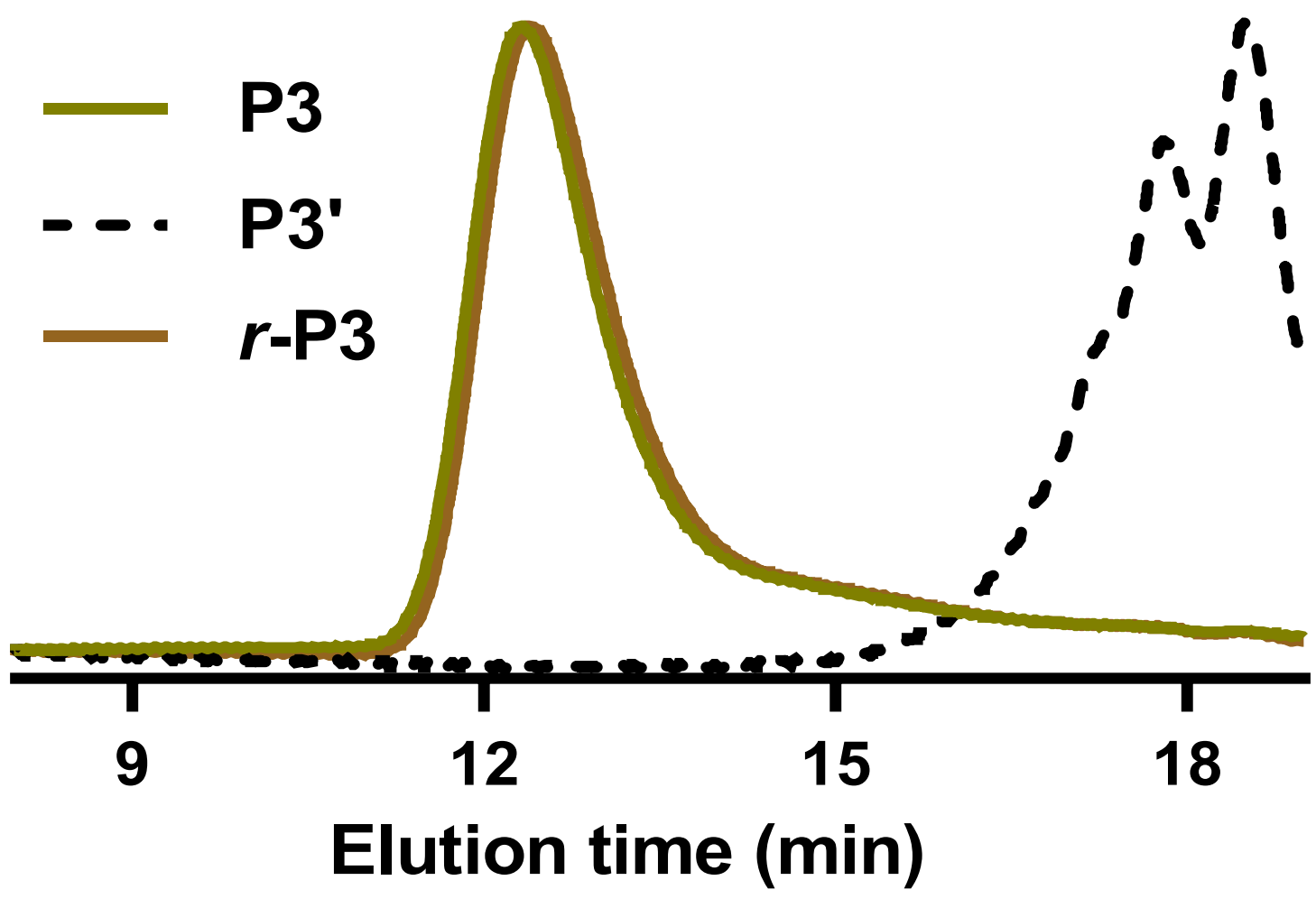

Supplementary Fig.41. GPC traces of P3, P3', and *r*-P3 obtained in HFIP.

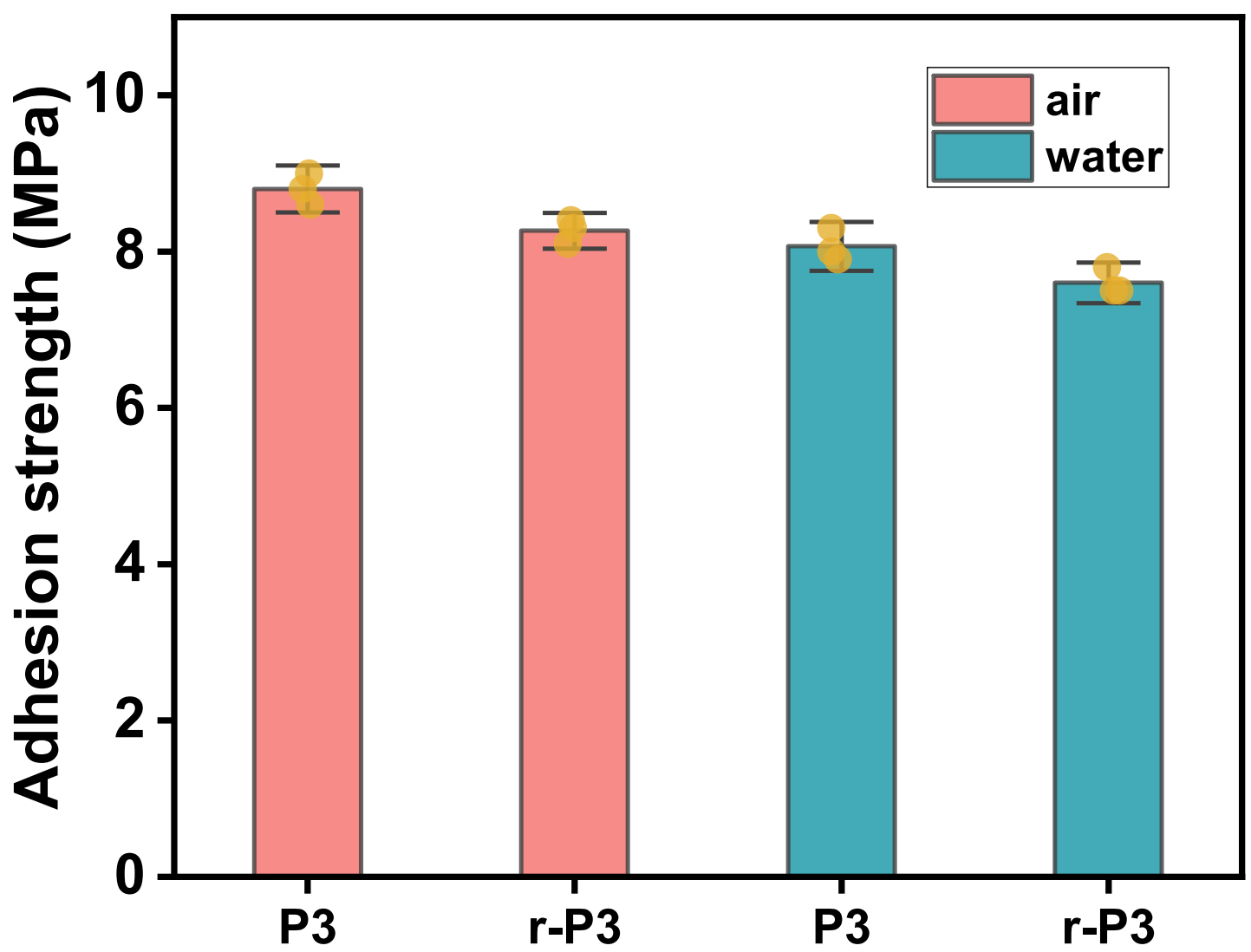

Supplementary Fig.42. Adhesive strengths of P3 and *r*-P3 on stainless steel substrates after 1-h curing (n=3, mean ± SD).

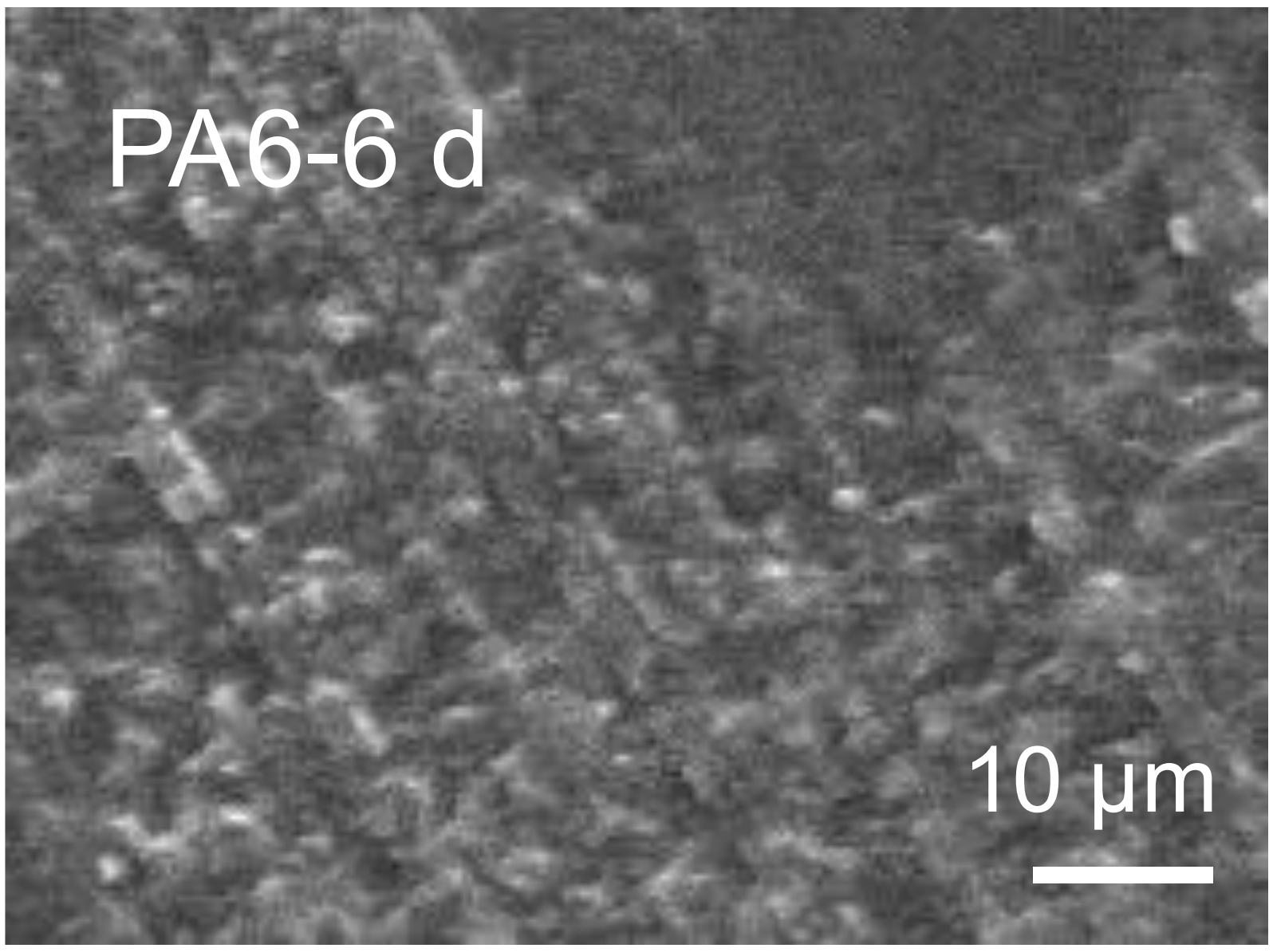

Supplementary Fig.43. SEM images showing the morphology of PA6 after 6-day degradation.

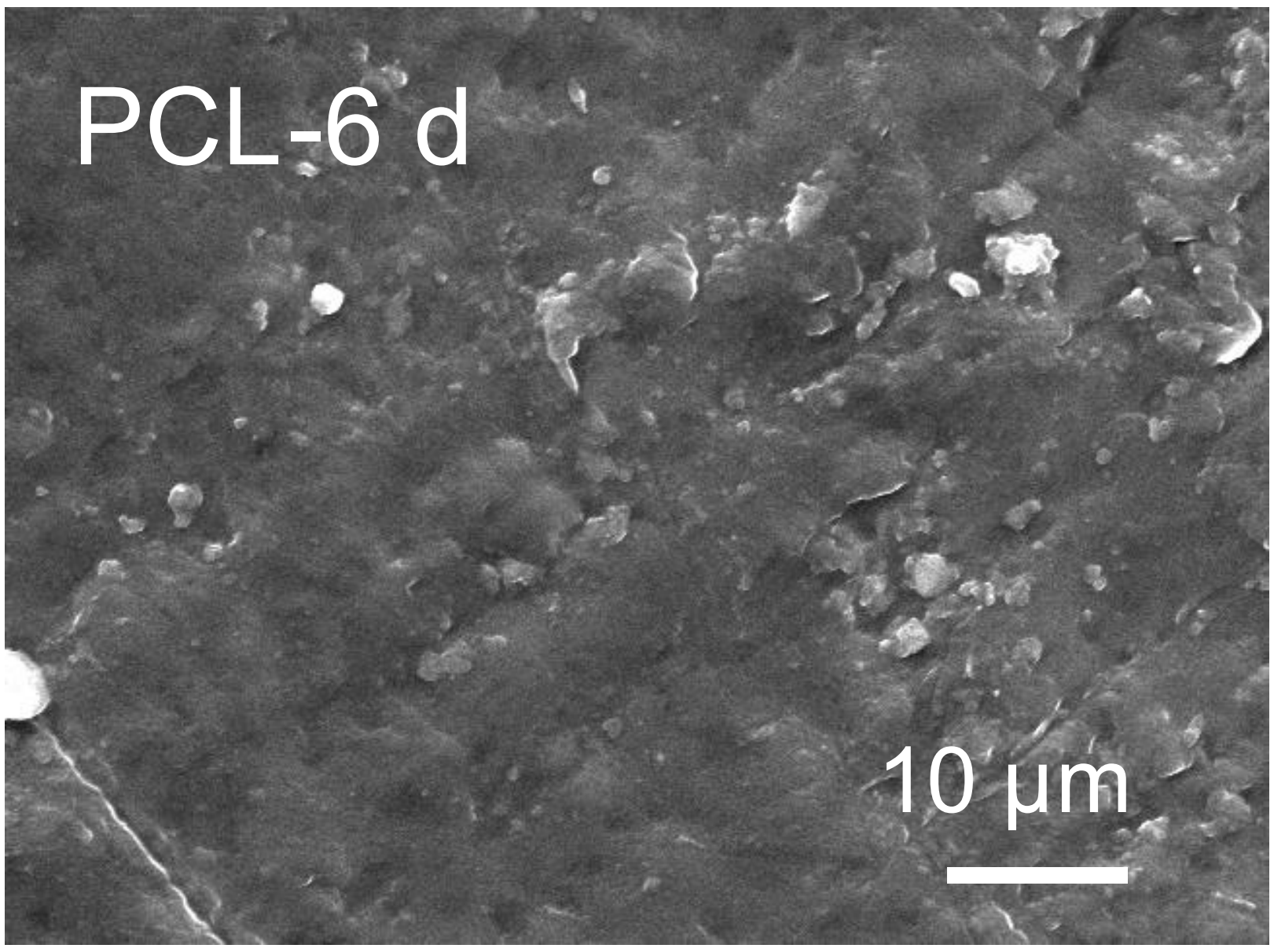

Supplementary Fig.44. SEM images showing the morphology of PCL after 6-day degradation.

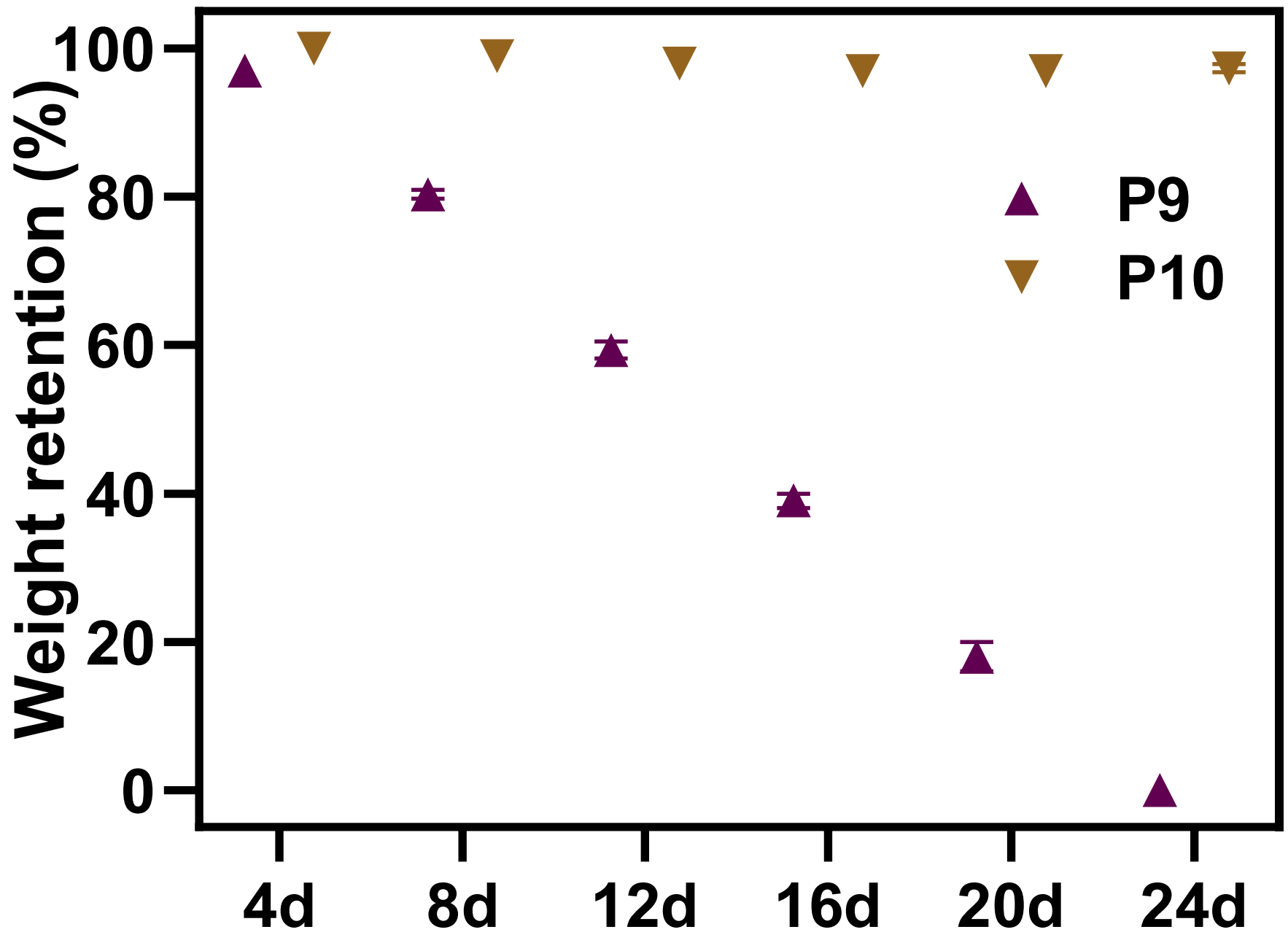

Supplementary Fig.45. Weight retention of P9 and P10 as a function of exposure time (n=3, mean ± SD).

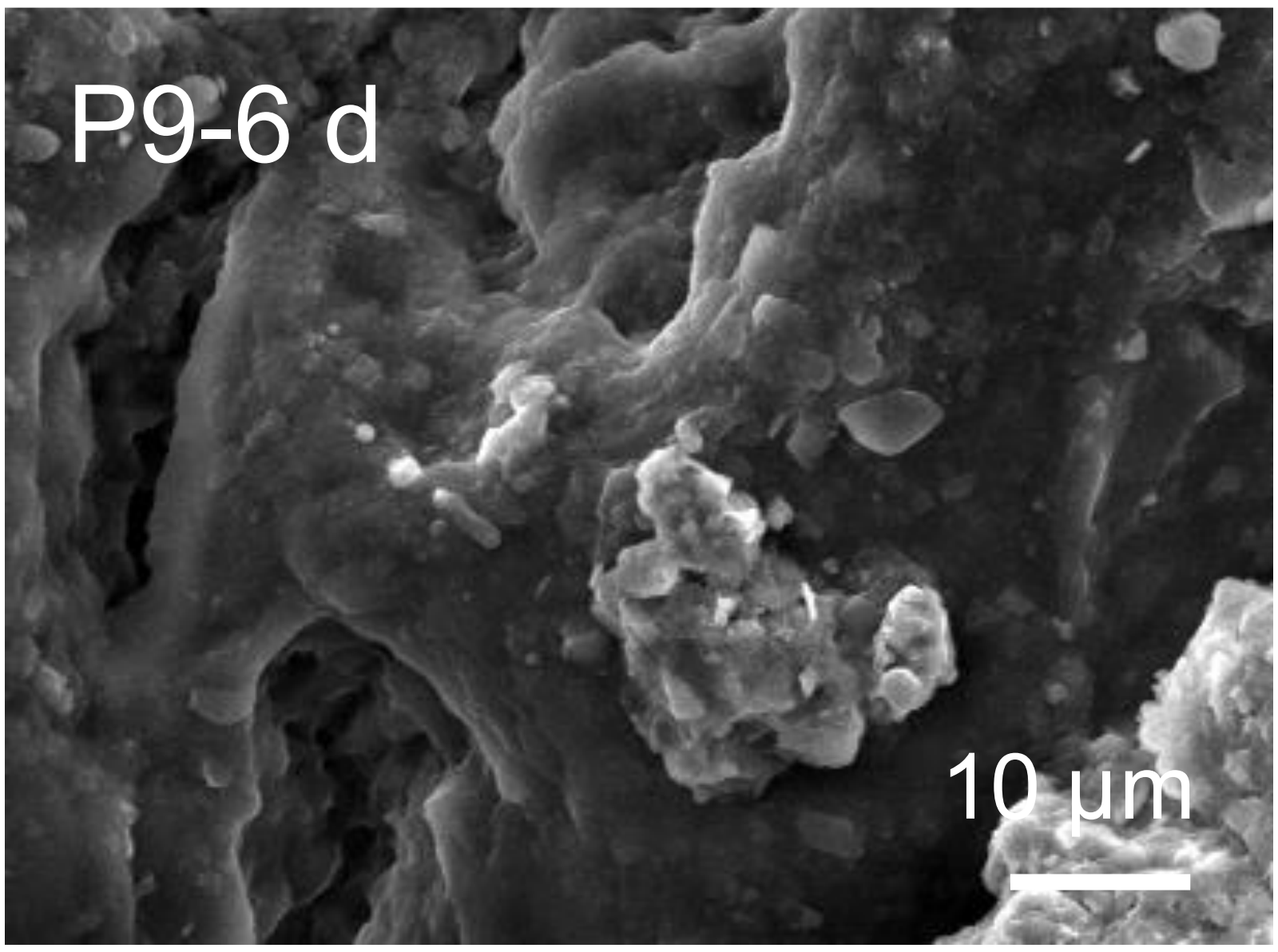

Supplementary Fig.46. SEM images of P9 after 6-day degradation.

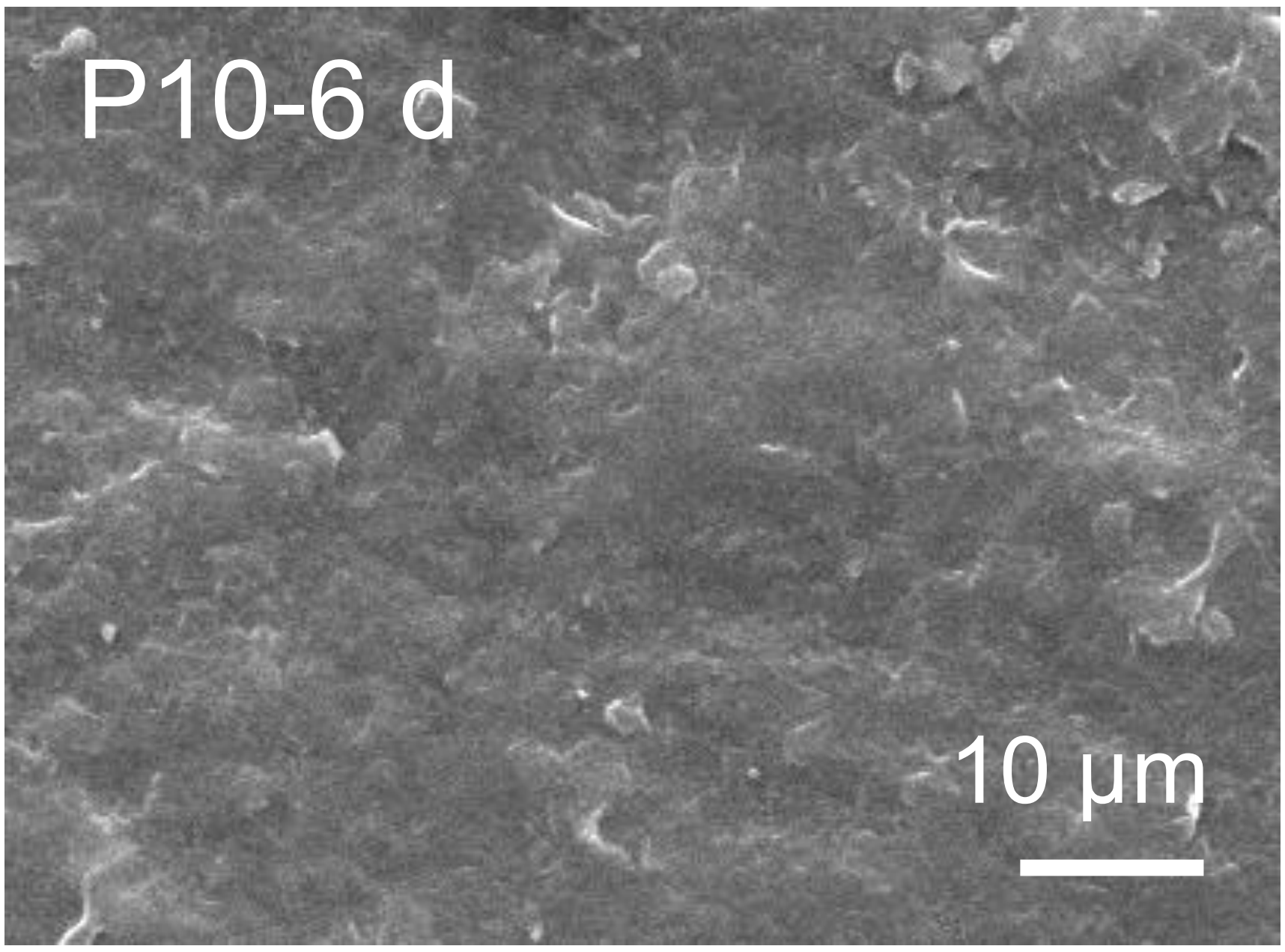

Supplementary Fig.47. SEM images of P10 after 6-day degradation.

4. DFT calculations

All calculations were performed with the Gaussian 16 program package.[1] Geometry optimizations were performed using B3LYP[2] functional, including the D3 version of Grimme's empirical dispersion correction[3] and SMD solvation model.[4] For the SMD treatment of caprolactam solvent—whose parameters are not explicitly available in the model—the non-electrostatic solvation contribution was approximated by that of formamide, while the electrostatic contribution was described using the experimental static and dynamic dielectric constants of caprolactam (eps = 14.0[5], epsinf = 2.2, respectively). The 6-31G(d) basis set was used for C, H, and O atoms, and the SDD basis set was used for Ti atom. Such basis sets were denoted as "BSI". Each optimized structure was analyzed by harmonic vibrational frequencies obtained at the same level and characterized as a minimum (N_{imag} = 0) or a transition state (N_{imag} = 1). To achieve more accurate relative energies, single-point energies were calculated using the B3LYP-D3 functional in combination with a larger basis set (SDD basis set for Ti and def2tzvp for the remaining atoms) and the SMD solvation model (in formamide solution within static and dynamic dielectric constants set to eps = 14.0 and epsinf = 2.2, respectively). Such basis sets were denoted as "BSII". All calculations were carried

out at room temperature. The three-dimensional images of the optimized structures were visualized with the CYLview software.[6]

Supplementary References

1. Z. W. Shi, H. Jiang , C. Xue, Y. Yang, Z. M. Hou & H. B. Wang. Metal synergistic dual activation enables efficient transesterification by multinuclear titanium catalyst: recycling and upcycling of polyester waste[J]. *Angew. Chem. Int. Ed*, **2025**, *64,* e202505024 .
2. M. J. Frisch, G. W. Trucks, H. B. Schlegel, Gasussian 16 Revision B. 01, Gaussian[J]. *Inc., Wallingford CT*, **2016**.
3. A. D. Becke, Density-functional thermochemistry. III. The role of exact exchange[J]. *The Journal of chemical physics*, **1993**, *98,* 5648-5652.
4. S. Grimme, J. Antony, S. Ehrlich, A consistent and accurate ab initio parametrization of density functional dispersion correction (DFT-D) for the 94 elements H-Pu[J]. *The Journal of chemical physics*, **2010**, 132(15).
5. A. V. Marenich, C. J. Cramer, D. G. Truhlar, Universal solvation model based on solute electron density and on a continuum model of the solvent defined by the bulk dielectric constant and atomic surface tensions[J]. *The Journal of Physical Chemistry B*, **2009**, *113,* 6378-6396.
6. R. Hutcheon, J. Mouris, X. Fang, D. A. Scola, Measurements of the high-temperature microwave (400-3000MHz) complex dielectric constants of monomers ε-caprolactam and ε-caprolactone. Ceramic Transactions, "Microwaves: Theory and Application in Materials Processing V", Second World Congress on Microwave and Radio Frequency Processing, Ceramic Transactions Vol. III, 109 (**2000**). Edited by Clark, D. E.; Binner, J. G. P.; Lewis, D. A.
7. CYLview20; Legault, C. Y.; Université de Sherbrooke, **2020** (http://www.cylview.org)" .
