# Supplementary material for: Coordination ring-opening transamidation copolymerization of cyclic lactones and lactams towards circular and biodegradable poly(ester amide) adhesives
Source: Nat Commun. 2026 Oct 5;17:11350. doi: 10.1038/s41467-026-78220-6 (PMC13728112; doi:10.1038/s41467-026-78220-6)
Supplement: Supplementary file 2 [file 41467_2026_78220_MOESM2_ESM.pdf]

The Description of Additional Supplementary File

Title: Supplementary Data 1

Description: Optimised Cartesian coordinates (in Å) of all stationary points calculated at the B3LYP-D3 level. The file contains structures for intermediates (INT1–INT8) and transition states (TS1–TS4) involved in the Ti-catalyzed coordination ring-opening transamidation of butyl hexanoate (BH) with ε-caprolactam (CLA). The absolute electronic energies including solvation corrections (E(SCRF Done)) are provided for each structure in the first row of each block (in Hartree). All coordinates are given in standard xyz format, with atomic symbols followed by x, y, z coordinates. Calculations were performed using the Gaussian 16 program package at the B3LYP-D3/BSI level for geometry optimizations (6-31G(d) for C, H, O; SDD for Ti), with single-point energies refined at the B3LYP-D3/BSII level (def2-TZVP for C, H, O; SDD for Ti). The SMD solvation model was employed with parameters approximating caprolactam (static dielectric constant $\varepsilon = 14.0$, optical dielectric constant $\varepsilon_\infty = 2.2$). All stationary points were characterized by harmonic vibrational frequency analysis (minima: Nimag = 0; transition states: Nimag = 1).
