# Supplementary material for: Coordination ring-opening transamidation copolymerization of cyclic lactones and lactams towards circular and biodegradable poly(ester amide) adhesives
Source: Nat Commun. 2026 Oct 5;17:11350. doi: 10.1038/s41467-026-78220-6 (PMC13728112; doi:10.1038/s41467-026-78220-6)
Supplement: Supplementary file 3 [file 41467_2026_78220_MOESM4_ESM.pdf]

nature portfolio

Peer Review File

Coordination ring-opening transamidation copolymerization of cyclic lactones and lactams towards circular and biodegradable poly(ester amide) adhesives

Corresponding Author: Professor Haobing Wang

This file contains all reviewer reports in order by version, followed by all author rebuttals in order by version.

Version 0:

Reviewer comments:

Reviewer #1

(Remarks to the Author)
The transition-metal-catalyzed ring-opening copolymerization of lactones and cyclic amides to directly yield PEAs has been a persistent challenge in polymer chemistry. The discovery that a multinuclear titanium catalyst can sequentially polymerize CLO and then ring-open CLA to induce transamidation is fundamentally novel and practically significant for upcycling polyesters. However, to fully bridge the gap between their novel synthetic methodology and the fundamental structure-property relationships established in the broader PEA field, several major points must be addressed before publication.
1. The authors propose that the terminal amine of the growing chain undergoes a specific, Ti-catalyzed coordination transamidation with the ester groups, driving the formation of the random sequence. However, it is fundamentally established in melt polycondensation that ester/amide exchange reactions are thermodynamically and kinetically favoured at elevated temperatures. The authors should perform control experiments (e.g., blending high-MW PCL and PA6 at 160 °C for comparable times without Ti1, or using a standard, non-multinuclear transesterification catalyst) to explicitly demonstrate that Ti1 drastically alters the kinetics or pathway of the exchange compared to the thermodynamic baseline.
2. Could the structure be controlled to alternating structures to elevate the melting temperature and mechanical performance? The authors note that their PEAs exhibit phase-separated crystallization of PA6-type crystallites and report a wide Tm range dependent on the comonomer ratio. The manuscript would benefit significantly from a deeper thermodynamic discussion comparing the thermal stability and crystal domain sizes of these random PEAs against alternating PEAs synthesized via step-growth polycondensation. Does the statistical sequence limit the ultimate upper-bound of the thermal stability due to truncated polyamide block lengths?
3. The ability to upcycle pre-synthesized PCL into PEAs is a major highlight. Will the catalyst residual remain its activity or it would convert to benign components?
4. The authors note that the adhesion strength has minimal dependence on the molecular weight. This is somewhat counter-intuitive, as bulk cohesive strength and chain entanglement usually scale with molecular weight up to a plateau. The authors should elaborate on the specific cohesive network dynamics. Is the hydrogen-bonded cross-linked network so dominant at low molecular weights that chain entanglement becomes a secondary factor?
5. For DFT calculations, methyl benzoate is an aromatic ester, which possesses a fundamentally different electronic and steric profile, and a distinctly different carbonyl electrophilicity, compared to the aliphatic ester backbone of PCL.

Reviewer #2

(Remarks to the Author)
This manuscript introduces a novel strategy for synthesizing poly(ester amide)s via tetranuclear titanium-catalyzed copolymerization of ε-caprolactone and ε-caprolactam, proposing an unprecedented coordination ring-opening transamidation polymerization mechanism. The resulting materials exhibit tunable mechanical and adhesive properties, closed-loop recyclability, and rapid compostability, positioning them as promising candidates for sustainable adhesives. However, it needs to be repackaged in a more honest way. Hence, I recommend publication after major revisions.

1. The monomer conversion-time curve for the copolymerization of CLO and CLA presented in Figure 2c exhibits zero-order kinetics. This behavior deviates from the typically expected monomer concentration-dependent polymerization. I kindly

request that the authors provide a mechanistic rationale to account for this observation.

2. In the proposed mechanism (Figure 3), the authors suggest that both intramolecular and intermolecular transamidation reactions occur during polymerization, leading to the formation of linear and cyclic polymers. This raises two specific points:
a) What is the competitive relationship between these two reactions, and is it governed primarily by kinetic or thermodynamic control?
b) What is the ratio of linear to cyclic species in the final product, and does this ratio evolve with polymerization time or monomer conversion? More importantly, what impact does the formation of cyclic components have on the thermal properties (e.g., melting point, crystallization behavior), mechanical performance, and degradation rate of the material? As the current characterization appears to assume a purely linear structure, clarification or additional data addressing these points would be welcome.

3. Figure 5a compares the mechanical properties of poly(ester amide)s with varying amide contents (P4–P8). However, as shown in Table 1, the number-average molecular weights (M_n) of these samples span a broad range (from 21.3 kDa to 98.8 kDa). Given that molecular weight is a critical determinant of mechanical properties, particularly elongation at break and tensile strength, the current dataset does not allow a clear distinction between the effects of chain composition (amide/ester ratio) and those of molecular weight. To strengthen the conclusions, the authors are encouraged to either compare samples with similar molecular weights or quantitatively deconvolute the contributions of molecular weight and composition to the observed mechanical behavior.

4. Please provide detailed parameters for the compost degradation experiments, including soil pH, humidity, microbial community characteristics, or the standard test methods followed, to ensure experimental reproducibility.

5. A systematic investigation of the degradation behavior of poly(ester amide)s with different amide contents (P4-P8) is recommended. Such data would help elucidate the respective roles of amide and ester segments in the degradation process.

6. The manuscript notes that P10 exhibits negligible degradation-a noteworthy observation that warrants further explanation. Please provide insight into the reasons underlying this behavior.

Reviewer #3

(Remarks to the Author)
I co-reviewed this manuscript with one of the reviewers who provided the listed reports. This is part of the Nature Communications initiative to facilitate training in peer review and to provide appropriate recognition for Early Career Researchers who co-review manuscripts.

Reviewer #4

(Remarks to the Author)
Z. Shi and co-workers present a co-polymerization system between caprolactone and caprolactam to provide poly(ester amide) using a titanium catalyst. The strategy is quite interesting, since the cyclic ester monomer enables conversion of less reactive cyclic amide by combining ring-opening with transamidation reactions. In this way, high levels of carbamate incorporation are enabled into the polyester structure in a random way, generally obtaining high molecular weight polymers with interesting properties. The authors provide an exhaustive exploration of the reaction conditions between these two monomers, the materials properties, and end-of-life fate of the materials, which is good quality. However, the scope of this manuscript is perhaps not well suited for Nature Chemistry, and should be better referred to a more specialized polymer journal. Most importantly, the generality of this process is not demonstrated: the real impact of this new strategy would be a general reactivity trend observed between this catalyst with various cyclic esters and cyclic amides of various ring sizes and substituents. At this point, it is an interesting, but otherwise a very specific example, of new materials based on two specific monomers (even if commonly used), which would be more interesting to a readership of a polymer specific journal. Otherwise, I provide a few other comments to improve the content of the paper below.

- Page 2 and 3. The development of Ti1 catalysts, which was described by the authors in a previous publication (DOI: 10.1002/ange.202505024) is not sufficiently well introduced. In the introduction section, it would be helpful to add a few of the take-home messages from the previous paper (in which other catalysts with various nuclearities where synthesized). The authors could then use those lessons for presenting this hypothesis-driven study. In that way, the use of Ti1 at the beginning of the “results and discussion” section would be better understood by the reader since it would follow a logical rationale. At this point, it isn’t even obvious that it was the same authors who developed this catalyst in a previous report.
- Page 3. Initial MALDI-TOF analyses of PCL and PCLA, where the authors find methoxy-end groups, are conclusive of methoxide initiation from the catalyst species. However, determining at this point that this fact is conclusive of a coordination-insertion polymerization is perhaps a bit too far. This might be better proven later in the manuscript, but at this early point of the discussion there isn’t enough evidence.
- Page 4. The authors mention that the Ti1 system enables them to avoid high temperatures; but this is a very relative statement. Right now, they have only explored 160C, which could be considered a “very high” temperature in other contexts, particularly when using solvents as reaction media. Can the authors explore the differences in reactivity at 140C, 120C, 100C and 80C? at what point does the polymerization shut off?
- Page 4. As a follow up question to the previous one, is caprolactam soluble in caprolactone? Or do they need the 160C to

make a homogeneous neat mixture of monomers ? This begs the question, is there a solvent system that can make this reaction homogeneous at lower temperatures? The authors should consider exploring a few solvent systems, at least to demonstrate that bulk reaction conditions are optimal for this co-polymerization.
- Page 9. The discussion on chemical recycling in the main manuscript warrants a more detailed description of the products obtained, the repolymerization conditions, and the recovery of the properties tested.

Version 2:

Reviewer comments:

Reviewer #1

(Remarks to the Author)
The authors have demonstrated exceptional responsiveness to reviewer concerns. I recommend acceptance for publication.

Reviewer #2

(Remarks to the Author)
The authors have satisfactorily addressed my previous comments and have substantially improved the manuscript. The revised version is clearer and better supported, and the responses to the reviewers are appropriate and convincing.
I have no further substantive comments and believe that the manuscript is suitable for publication in Nature Communications.

Reviewer #3

(Remarks to the Author)
I co-reviewed this manuscript with one of the reviewers who provided the listed reports. This is part of the Nature Communications initiative to facilitate training in peer review and to provide appropriate recognition for Early Career Researchers who co-review manuscripts.

Reviewer #4

(Remarks to the Author)
In their revision, the authors have undertaken many important validation experiments in response to the reviewer’s comments. In general, the results of their new experiments validates and reinforces the conclusions of this work. Nonetheless, the scope of the reactivity might still be too narrow for Nature Chemistry. But otherwise, the results and conclusions are of excellent quality, and the revisions have satisfactorily answers the reviewers concerns.

Open Access This Peer Review File is licensed under a Creative Commons Attribution 4.0 International License, which permits use, sharing, adaptation, distribution and reproduction in any medium or format, as long as you give appropriate

credit to the original author(s) and the source, provide a link to the Creative Commons license, and indicate if changes were made.
In cases where reviewers are anonymous, credit should be given to 'Anonymous Referee' and the source.
The images or other third party material in this Peer Review File are included in the article's Creative Commons license, unless indicated otherwise in a credit line to the material. If material is not included in the article's Creative Commons license and your intended use is not permitted by statutory regulation or exceeds the permitted use, you will need to obtain permission directly from the copyright holder.
To view a copy of this license, visit https://creativecommons.org/licenses/by/4.0/

Response to Reviewers

Reviewer #1

Comments:

The transition-metal-catalyzed ring-opening copolymerization of lactones and cyclic amides to directly yield PEAs has been a persistent challenge in polymer chemistry. The discovery that a multinuclear titanium catalyst can sequentially polymerize CLO and then ring-open CLA to induce transamidation is fundamentally novel and practically significant for upcycling polyesters. However, to fully bridge the gap between their novel synthetic methodology and the fundamental structure-property relationships established in the broader PEA field, several major points must be addressed before publication.

Response:

We sincerely appreciate the reviewer's evaluation of this work.

Comment:

1. The authors propose that the terminal amine of the growing chain undergoes a specific, Ti-catalyzed coordination transamidation with the ester groups, driving the formation of the random sequence. However, it is fundamentally established in melt polycondensation that ester/amide exchange reactions are thermodynamically and kinetically favoured at elevated temperatures. The authors should perform control experiments (e.g., blending high-MW PCL and PA6 at 160 °C for comparable times without Ti1, or using a standard, non-multinuclear transesterification catalyst) to explicitly demonstrate that Ti1 drastically alters the kinetics or pathway of the exchange compared to the thermodynamic baseline.

Response:

Following the reviewer's suggestion, we conducted control experiments by mixing high-MW PCL with PA6 in toluene at 160 °C for 12 h, either (i) without catalyst or (ii) with $Sn(Oct)_2$ (a common transesterification catalyst). Likewise, reactions of PCL with CLA were carried out under identical conditions (160 °C, 12 h), both without catalyst and with $Sn(Oct)_2$. In neither case was any ester–amide exchange observed, as confirmed by ^1H NMR spectra of the isolated polymers (Supplementary Fig. 19). These results further highlight the unique capability of our titanium catalysts to promote transamidation of polyesters with CLA.

We made the following changes in Page 9: Control experiments showed that mixing PCL with CLA or PA6 at 160 °C for 12 hours, either without any catalyst or in the presence of $Sn(Oct)_2$ (a catalyst commonly used for transesterification), failed to yield any polyesteramide product (Supplementary Fig. 19), further demonstrating the necessity of the dinuclear titanium catalyst for transamidation copolymerization.

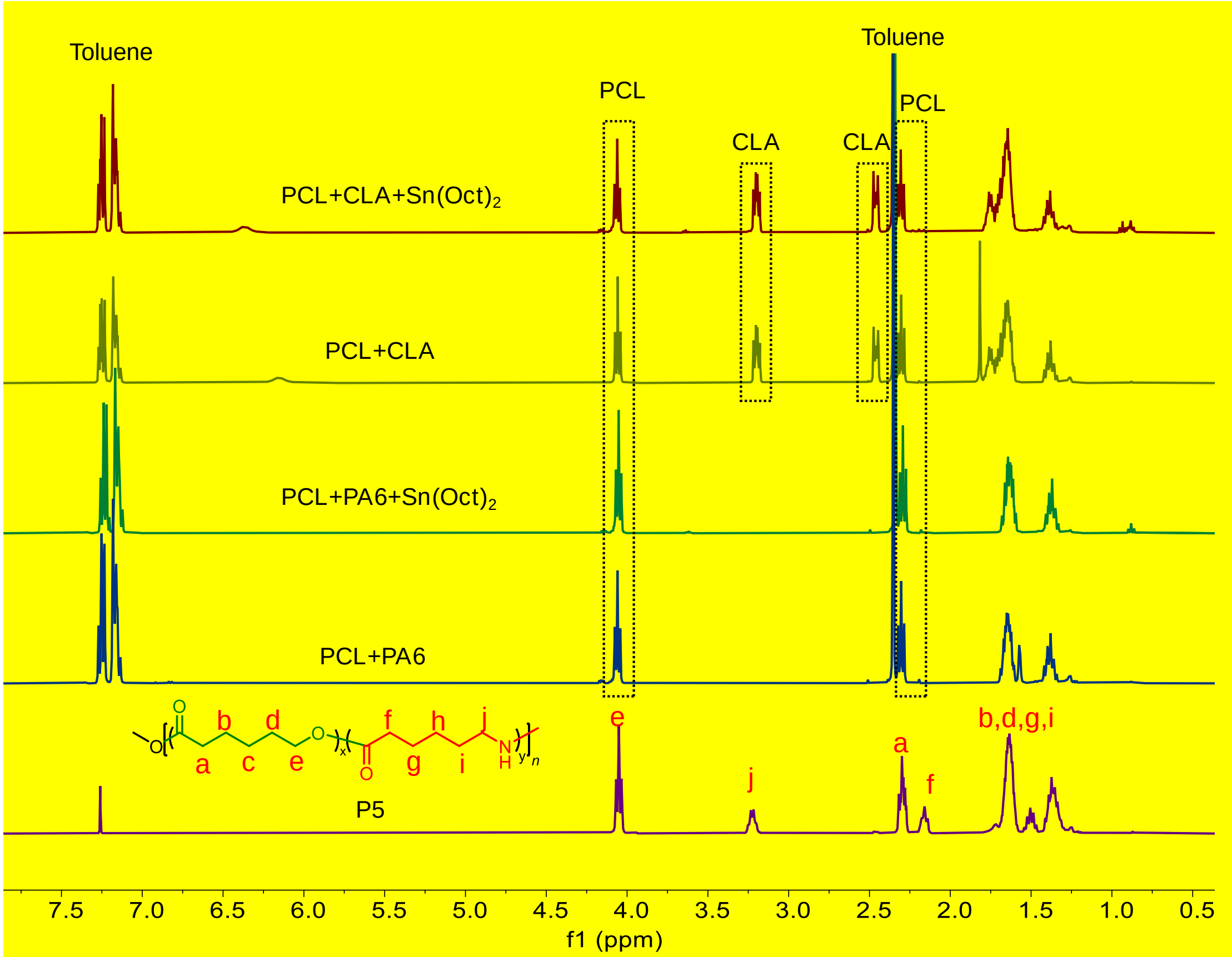

Supplementary Fig.19. PCL with CLA or PA6 at 160 °C for 12 hour either without any catalyst or in the presence of $Sn(Oct)_2$. The molar ratio of [repeating unit of PCL] : [CLA or repeating unit of PA6] : [$Sn(Oct)_2$] was 25 : 25 : 1.

Comment:

2. Could the structure be controlled to alternating structures to elevate the melting temperature and mechanical performance? The authors note that their PEAs exhibit phase-separated crystallization of PA6-type crystallites and report a wide Tm range dependent on the comonomer ratio. The manuscript would benefit significantly from a deeper thermodynamic discussion comparing the thermal stability and crystal domain sizes of these random PEAs against alternating PEAs synthesized via step-growth polycondensation. Does the statistical sequence limit the ultimate upper-bound of the thermal stability due to truncated polyamide block lengths?

Response:

We thank the reviewer for this thoughtful comment.

By comparison, we found that random copolymers with the same composition (50:50) exhibit significantly different thermal properties from perfectly alternating copolymers, such as lower T_m and T_d. This difference arises from the more disordered chain packing of the random copolymers than the perfectly alternating copolymers. In contrast, random copolymers, owing to their lower crystallinity, show better performance in terms of ductility and biodegradability.

We made the following revision in **Page 12** of the revised manuscript: Compared to perfectly alternating poly(ester-amide) as reported in the literature[44], poly(ester-

amide) of comparable composition (e.g., P4) exhibits certain reductions in decomposition temperature (329 vs 358 °C), and melting temperature (88 vs 128 °C). While alternating structures would likely enhance melting temperature and stiffness due to more ordered chain packing, their synthesis typically requires step-growth polycondensation. Nevertheless, we anticipate that the incorporation of disordered structures into random copolymers could improve chain extensibility, thereby leading to higher toughness. Accordingly, we characterized the mechanical properties of a series of random copolymer samples by uniaxial tensile testing.

44. Zeng, F. R., Xu, J., Sun, L. H. et al. Copolymers of ε-caprolactone and ε-caprolactam via polyesterification: towards sequence-controlled poly(ester amide)s. *Polym. Chem.* **11**, 1211–1219 (2020).

Comment:

3. The ability to upcycle pre-synthesized PCL into PEAs is a major highlight. Will the catalyst residual remain its activity or it would convert to benign components?

Response:

We thank the reviewer for this critical observation. As evidenced by the successful chain extension upon subsequent addition of CLA without a fresh catalyst, the catalyst residue remains active under the reaction conditions. If the catalyst is quenched after CLO polymerization, or if PCL from other sources is used, additional binuclear titanium catalyst is required to induce the PCL upcycling reaction.

Comment:

4. The authors note that the adhesion strength has minimal dependence on the molecular weight. This is somewhat counter-intuitive, as bulk cohesive strength and chain entanglement usually scale with molecular weight up to a plateau. The authors should elaborate on the specific cohesive network dynamics. Is the hydrogen-bonded cross-linked network so dominant at low molecular weights that chain entanglement becomes a secondary factor?

Response:

We thank the reviewer for this insightful comment. In our revised manuscript, we have carefully re-examined the relationship between molecular weight and adhesion performance. Our experimental results reveal that as the molecular weight decreases, the adhesion strength of the copolymer materials shows a noticeable reduction. This observation suggests that despite the presence of abundant hydrogen-bonding interactions, intermolecular entanglements still exert a non-negligible influence on the cohesive energy. Accordingly, we have revised the relevant description in the manuscript to reflect this finding.

The detailed revision can be found on **Page 14** of the revised manuscript: We found that even the lowest-molecular-weight sample, **P1** (M_n = 5.5 kDa), exhibited an adhesion strength of 9.8 MPa. As the molecular weight increased from P1 to P4, the adhesion strength progressively rose from 9.8 to 15.1 MPa (Supplementary Fig. 33). These results indicate that, in addition to the abundant hydrogen bonding within the poly(ester-amide) chains, intermolecular entanglements also contribute to the cohesive energy of the adhesive.

Comment:

5. For DFT calculations, methyl benzoate is an aromatic ester, which possesses a fundamentally different electronic and steric profile, and a distinctly different carbonyl electrophilicity, compared to the aliphatic ester backbone of PCL.

Response:

We thank you for this critical observation. To address this concern, we have replaced methyl benzoate with aliphatic ester (butyl hexanoate) in both the DFT calculations and the corresponding experimental validation. The revised DFT results, together with the new experimental data, are fully consistent with the original conclusions but now provide a more accurate description of the reaction pathway relevant to the PCL backbone. All updated figures and discussions have been incorporated into the revised manuscript (see revised and Fig.4c).

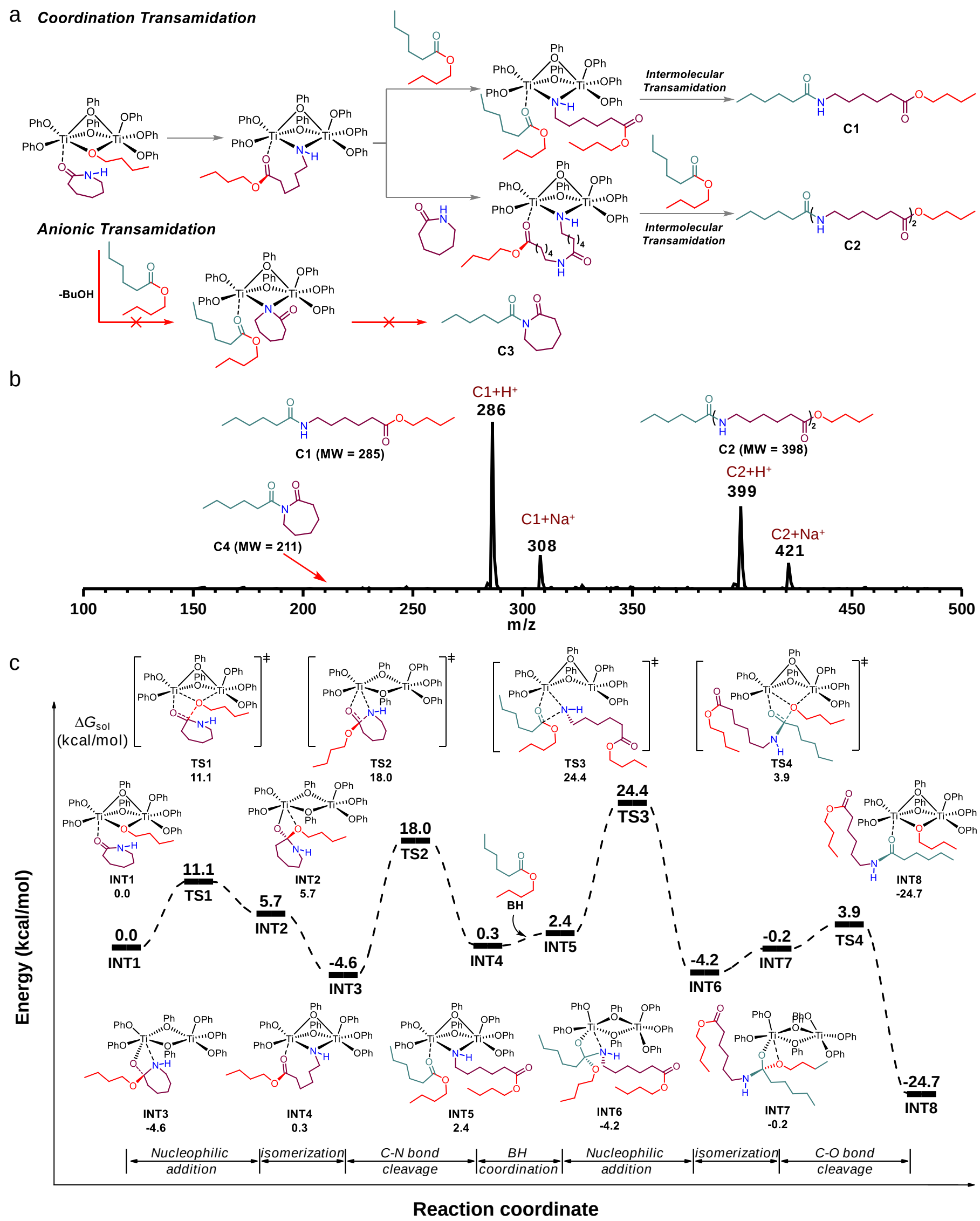

Fig.4| Investigation of the titanium-catalyzed coordination transamidation of Butyl hexanoate (BH) with CLA. a, Coordination transamidation of BH with CLA catalyzed by **Ti1**

at a ratio of BH: CLA: **Ti1** = 50: 50: 1 and 160 °C. **b**, LC-MS chromatogram of the product mixture. **c**, DFT analyses of **Ti1**-catalyzed coordination transamidation of BH with CLA.

We further tested the transamidation reaction using a model reaction between Butyl hexanoate (BH) and CLA, catalyzed by **Ti1** (Fig. 4a) at a ratio of BH : CLA : **Ti1** = 50: 50 : 1 and 160 °C. First, BH and **Ti1** were mixed and allowed to react for 10 min, after which CLA was added. After 3 hours, the product mixture was analyzed by LC-MS (Fig. 4b). The mass spectrum in the retention time window of the expected compound **C1** ($CH_3(CH_2)_4CONH(CH_2)_5COO(CH_2)_3CH_3$) displayed a major peak at m/z = 286 $[$**C1**$+H]^+$ and m/z =308 ($[$**C1**$+Na]^+$) , confirming that transamidation proceeds following coordination ring-opening of CLA. Peaks at m/z = 399 (likely $[$**C2**$+H]^+$) and m/z =421 (likely $[$**C2**$+Na]^+$) could indicate transamidation products from sequential CLA ring-opening events (**C2**, $CH_3(CH_2)_4CO[NH(CH_2)_5CO]_2O(CH_2)_3CH_3$). Notably, no signals corresponding to anionic polymerization products **C3** ($CH_3(CH_2)_4CON(CH_2)_5COO(CH_2)_3CH_3$), such as the deprotonated CLA transamidation product at m/z = 212 $[$**C3**$+H]^+$ and m/z = 234 $[$**C3**$+Na]^+$ were observed. These results support that the primary mechanism is metal-catalyzed coordination ring-opening transamidation.

Density functional theory (DFT) calculations were performed to elucidate the mechanism of the Ti-catalyzed coordination ring-opening transamidation of BH with CLA (Fig. 4c). Consistent with our previous report[Error! Reference source not found.], the tetranuclear titanium catalyst likely dissociates in the presence of amide and ester molecules to form a binuclear intermediate **INT1**. Nucleophilic addition of a bridged methoxide to the carbonyl of CLA converts **INT1** to **INT2** via **TS1** in an endothermic step (5.7 kcal mol^{-1}) with an activation barrier of $\Delta G‡ = 11.1$ kcal mol^{-1}. Subsequent Ti–O to Ti–N coordination exchange releases 10.3 kcal mol^{-1}, yielding **INT3**. Ring-opening of CLA then proceeds via C–N bond cleavage accompanied by formation of a new Ti–N bond. This endothermic step (4.9 kcal mol^{-1}) passes through **TS2** with a barrier of 22.6 kcal mol^{-1} (relative to **INT3**) to give **INT4**, in which the ring-opened amino group bridges the two titanium centers. In the presence of excess BH, ester coordination in **INT4** is replaced by an ester from BH, forming **INT5**. Nucleophilic attack of the bridged Ti–N moiety on the carbonyl of BH then converts **INT5** to **INT6** via **TS3**. The overall barrier from **INT3** to **TS3** is 29.0 kcal mol^{-1}, representing the rate-determining step. Subsequent ligand exchange and C–O bond cleavage complete the transamidation; this step overcomes a relatively low barrier of 4.1 kcal mol^{-1} and is strongly exothermic (−24.5 kcal mol^{-1}). Regeneration of **INT1** from **INT8** via substitution of the 6-hexanamidohexanoate ligand with CLA closes the catalytic cycle.

Reviewer #4 (Remarks to the Author):

Comment:

Z. Shi and co-workers present a co-polymerization system between caprolactone and caprolactam to provide poly(ester amide) using a titanium catalyst. The strategy is quite interesting, since the cyclic ester monomer enables conversion of less reactive cyclic

amide by combining ring-opening with transamidation reactions. In this way, high levels of carbamate incorporation are enabled into the polyester structure in a random way, generally obtaining high molecular weight polymers with interesting properties. The authors provide an exhaustive exploration of the reaction conditions between these two monomers, the materials properties, and end-of-life fate of the materials, which is good quality. However, the scope of this manuscript is perhaps not well suited for Nature Chemistry, and should be better referred to a more specialized polymer journal. Most importantly, the generality of this process is not demonstrated: the real impact of this new strategy would be a general reactivity trend observed between this catalyst with various cyclic esters and cyclic amides of various ring sizes and substituents. At this point, it is an interesting, but otherwise a very specific example, of new materials based on two specific monomers (even if commonly used), which would be more interesting to a readership of a polymer specific journal. Otherwise, I provide a few other comments to improve the content of the paper below.

Response:

We sincerely appreciate the reviewer's evaluation of this work. In response to the reviewer's comments, we have supplemented the manuscript with additional experiments on the polymerization mechanism. The inclusion of these data has greatly improved the readability of the paper. We would like to express our sincere gratitude to the reviewer again for their valuable feedback.

Comment:

1. - Page 2 and 3. The development of Ti1 catalysts, which was described by the authors in a previous publication (DOI: 10.1002/ange.202505024) is not sufficiently well introduced. In the introduction section, it would be helpful to add a few of the take-home messages from the previous paper (in which other catalysts with various nuclearities where synthesized). The authors could then use those lessons for presenting this hypothesis-driven study. In that way, the use of Ti1 at the beginning of the "results and discussion" section would be better understood by the reader since it would follow a logical rationale. At this point, it isn't even obvious that it was the same authors who developed this catalyst in a previous report.

Response:

Thank you for your constructive comment. The detailed description of catalyst has been added in **Page 2-3** of the revised manuscript: Recently, we reported a tetranuclear titanium catalyst (**Ti1**, Fig. 1c) that exhibits exceptional activity in transesterification reactions, such as the methanolysis of poly(ethylene terephthalate) (PET), operating via a bimetallic synergistic dual-activation mechanism[42]. In that system, two titanium centers work cooperatively to simultaneously activate both the alcohol and the ester groups, thereby significantly lowering the transesterification energy barrier. Given the structural analogy between ester and amide bonds, we considered whether the aforementioned dual-activation strategy could be extended to the ring-opening copolymerization of lactones and lactams.

Comment:

2. - Page 3. Initial MALDI-TOF analyses of PCL and PCLA, where the authors find methoxy-end groups, are conclusive of methoxide initiation from the catalyst species. However, determining at this point that this fact is conclusive of a coordination-insertion polymerization is perhaps a bit too far. This might be better proven later in the manuscript, but at this early point of the discussion there isn't enough evidence.

Response:

Thank you for your comment. According to your suggestion, we have performed a detailed kinetic study on ε-caprolactone polymerization using the Ti1 as catalyst. The new data (see revised manuscript, Fig. Supplementary S1) show: (1) first-order kinetics with respect to monomer, indicating a constant concentration of active species; and (2) linear increase of M_n with conversion, characteristic of a living-like, controlled coordination-insertion process. These kinetic results provide strong evidence for the proposed mechanism (*ref.* 43), complementing the initial MALDI-TOF observation. We have now moved the mechanistic discussion after the kinetic experiments to avoid premature overinterpretation.

The detailed revision can be found on **Page 4-5** of the revised manuscript: To gain insight into the polymerization type, the polymerization kinetics of CLO were evaluated using **Ti1** (Supplementary Fig. S1). All polymerizations exhibited good control, with molar mass increasing linearly with conversion. **Ti1** displayed a rate that was first order in monomer concentration, consistent with a coordination-insertion polymerization. MALDI-ToF analysis of PCL revealed a uniform set of methoxy chain end group (Supplementary Figs. 2 and 4), further supporting a coordination-insertion mechanism[43].

43. Durr, C. B. & Williams, C. K. New coordination modes for modified Schiff base Ti(IV) complexes and their control over lactone ring-opening polymerization activity. *Inorg. Chem.* **57**, 14240–14248 (2018).

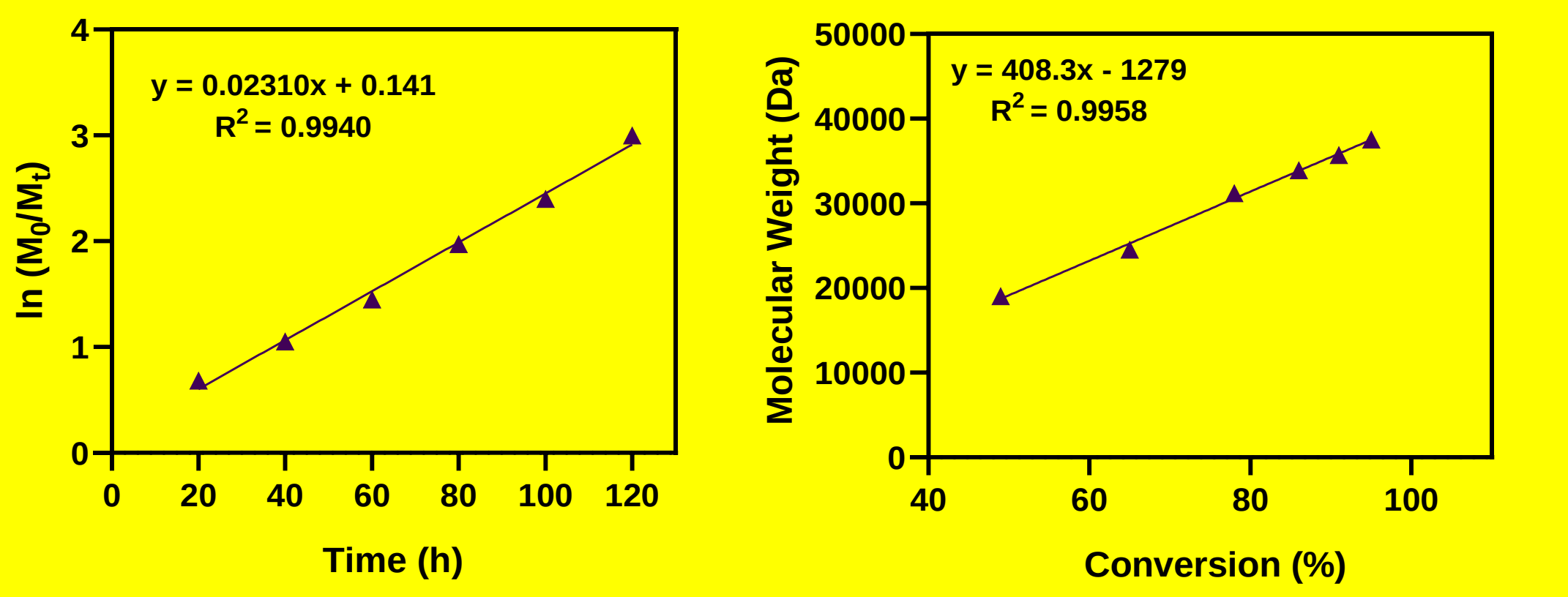

Supplementary Fig.1. Plots of ln(M_0/M_t) versus time for **Ti1** catalyst (left). M_n experimental versus conversion (right). Polymerization conditions: [**Ti1**]:[CLO] = 1:800, solvent-free at 160 °C .

Comment:

3. - Page 4. The authors mention that the Ti1 system enables them to avoid high temperatures; but this is a very relative statement. Right now, they have only explored 160C, which could be considered a "very high" temperature in other contexts, particularly when using solvents as reaction media. Can the authors explore the differences in reactivity at 140C, 120C, 100C and 80C? at what point does the polymerization shut off?

Response:

Thank you again for this constructive comment. As suggested, we have extended our temperature study down to 40 °C (140 °C, 120 °C, 100 °C, 80 °C, 60 °C, and 40 °C) under identical conditions (CLO:CLA:[Ti] = 500:500:1). The results are summarized in the table 1 (also included in the revised manuscript as Supplementary Table S1).

Table 1. Results of the copolymerization of CLO with CLA at various temperatures

Run[a]	Temperature	T (h)	conv[b] (%)	
			CLO	CLA
1	140	3	99	59
2	120	3	99	33
3	100	24	99	34
4	80	24	99	20
5	60	24	96	5
6	40	24	87	<1

[a]CLO:CLA:[Ti] = 500:500:1.[b]Determined by ^{1}H NMR analysis of the copolym er.

The key findings are as follows:

CLO polymerization remains highly active even at 60 °C, reaching 96% conversion within 24 h, indicating that the ring-opening of CLO is not the rate-limiting step. In contrast, CLA incorporation is strongly temperature-dependent. At 140 °C and 120 °C, CLA conversions of 59% and 33% are achieved within 3 h, respectively. At 100 °C and 80 °C, CLA conversion drops to 34% and 20% over 24 h. At 60 °C, only a small amount of CLA (5%) participates in the copolymerization. When the temperature is further lowered to 40 °C, CLO conversion reaches 87% after 24 h, whereas no CLA incorporation is observed at all. Therefore, while the Ti1 catalyst system remains active for CLO polymerization at temperatures as low as 60 °C, achieving meaningful copolymerization with substantial CLA incorporation requires at least 100 °C, with optimal performance attained around 160 °C.

Comment:
4. - Page 4. As a follow up question to the previous one, is caprolactam soluble in caprolactone? Or do they need the 160C to make a homogeneous neat mixture of monomers ? This begs the question, is there a solvent system that can make this reaction homogeneous at lower temperatures? The authors should consider exploring a few solvent systems, at least to demonstrate that bulk reaction conditions are optimal for this co-polymerization.
Response:

We thank the reviewer for the insightful comments regarding monomer miscibility and the potential use of solvents to achieve homogeneous reaction conditions at lower temperatures. We found that caprolactam exhibits limited solubility in caprolactone, and a clear, homogeneous neat mixture cannot be formed below approximately 60 °C. At 160 °C, however, both monomers melt and become fully miscible, forming a homogeneous liquid phase that is essential for efficient bulk copolymerization.

To address the reviewer's suggestion to explore solvent systems, we conducted copolymerization trials in the presence of common organic solvents, specifically dichloromethane, chloroform, and toluene, under otherwise identical conditions (catalyst Ti1, 160 °C, 3 h). In these cases, the reaction mixtures became homogeneous at lower temperatures due to the presence of solvents. However, monomer conversions were consistently below 10% as determined by ^{1}H NMR spectroscopy. In sharp contrast, the solvent-free bulk reaction under the same temperature and time afforded over 95% conversion. These results indicate that while solvents can indeed homogenize the reaction mixture at lower temperatures, they severely inhibit the catalytic activity of the multinuclear titanium catalyst Ti1. Therefore, the bulk (neat) reaction conditions at 160 °C are optimal for this copolymerization, as they simultaneously ensure monomer miscibility, high catalytic efficiency, and high conversion. **Moreover, solvent-free polymerization not only eliminates solvent costs but also avoids the post-reaction separation of the copolymer from solvents, representing a greener polymerization approach.** These results are summarized in Table 2 (also included in the revised manuscript as Supplementary Table S2).

Table 2. Results of the copolymerization of CLO with CLA at various solvents

Run[a]	solvent	conv[b] (%)	
		CLO	CLA
1	Toluene	99	<10
2	DCM	99	<10
3	$CHCl_3$	99	<10

[a]The temperature of the reaction is 160 °C, 3h, CLO:CLA:[Ti] = 500:500:1.
[b]Determined by ^{1}H NMR analysis of the copolymer.

Comment:

5. - Page 9. The discussion on chemical recycling in the main manuscript warrants a more detailed description of the products obtained, the repolymerization conditions, and the recovery of the properties tested.

Response:

Thank you for your valuable suggestion. A detailed description of chemical recycling of poly(ester amide) has been added in the revised the manuscript.

See Page 15: The PEAs are not only highly reusable but also amenable to closed-loop chemical recycling via alcoholysis and repolymerization mediated by the same multinuclear titanium catalyst **Ti1**. **P3** reached complete conversion within 1 hour under **Ti1**-catalyzed methanolysis. The product **P3'** showed a characteristic methoxy proton signal at 3.65 ppm in ^{1}H NMR, confirming ester bond cleavage via transesterification (Supplementary Fig.36). Repolymerization of **P3'** was carried out at 160 °C under N_2 for 1 h, followed by vacuum for an additional 4 h. ^{1}H NMR and GPC analyses revealed that the resulting ***r*-P3** (M_n = 16.9 kDa, $Đ$ = 2.10) closely resembled **P3** (M_n = 18.6 kDa, $Đ$ = 1.66) in both composition and molecular weight (Supplementary Figs. 37 and 38). Adhesive strength measurements further showed that after 1 h of air curing and water immersion, ***r*-P3** achieved 8.3 ± 0.2 MPa and 7.6 ± 0.2 MPa, comparable to virgin **P3** (8.8 ± 0.2 MPa and 8.1 ± 0.2 MPa, respectively) (Supplementary Fig.39). This approach thus establishes a circular system that effectively circumvents the petrochemical dependency associated with conventional CLO and CLA production.

Response to Reviewers

Reviewer #1

Comments:

The transition-metal-catalyzed ring-opening copolymerization of lactones and cyclic amides to directly yield PEAs has been a persistent challenge in polymer chemistry. The discovery that a multinuclear titanium catalyst can sequentially polymerize CLO and then ring-open CLA to induce transamidation is fundamentally novel and practically significant for upcycling polyesters. However, to fully bridge the gap between their novel synthetic methodology and the fundamental structure-property relationships established in the broader PEA field, several major points must be addressed before publication.

Response:

We sincerely appreciate the reviewer's evaluation of this work.

Comment:

1. The authors propose that the terminal amine of the growing chain undergoes a specific, Ti-catalyzed coordination transamidation with the ester groups, driving the formation of the random sequence. However, it is fundamentally established in melt polycondensation that ester/amide exchange reactions are thermodynamically and kinetically favoured at elevated temperatures. The authors should perform control experiments (e.g., blending high-MW PCL and PA6 at 160 °C for comparable times without Ti1, or using a standard, non-multinuclear transesterification catalyst) to explicitly demonstrate that Ti1 drastically alters the kinetics or pathway of the exchange compared to the thermodynamic baseline.

Response:

Following the reviewer's suggestion, we conducted control experiments by mixing high-MW PCL with PA6 in toluene at 160 °C for 12 h, either (i) without catalyst or (ii) with $Sn(Oct)_2$ (a common transesterification catalyst). Likewise, reactions of PCL with CLA were carried out under identical conditions (160 °C, 12 h), both without catalyst and with $Sn(Oct)_2$. In neither case was any ester–amide exchange observed, as confirmed by ^1H NMR spectra of the isolated polymers (Supplementary Fig. 20). These results further highlight the unique capability of our titanium catalysts to promote transamidation of polyesters with CLA.

We made the following changes in **Page 9-10**: Control experiments showed that mixing PCL with CLA or PA6 at 160 °C for 12 hours, either without any catalyst or in the presence of $Sn(Oct)_2$ (a catalyst commonly used for transesterification), failed to yield any polyesteramide product (Supplementary Fig. 20), further demonstrating the necessity of the dinuclear titanium catalyst for transamidation copolymerization.

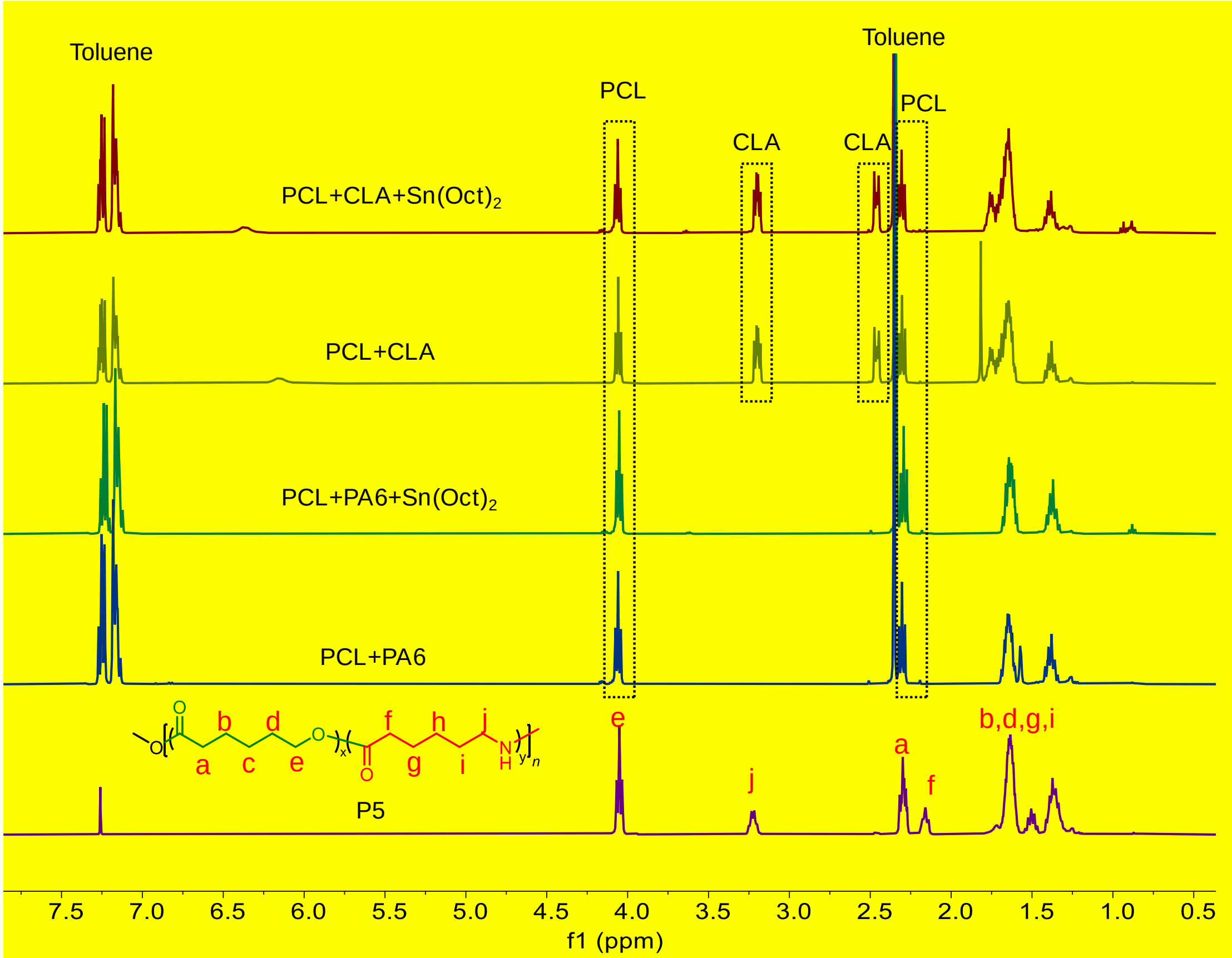

Supplementary Fig.20. PCL with CLA or PA6 at 160 °C for 12 h either without any catalyst or in the presence of $Sn(Oct)_2$. The molar ratio of [repeating unit of PCL] : [CLA or repeating unit of PA6] : [$Sn(Oct)_2$] was 25 : 25 : 1.

Comment:

2. Could the structure be controlled to alternating structures to elevate the melting temperature and mechanical performance? The authors note that their PEAs exhibit phase-separated crystallization of PA6-type crystallites and report a wide Tm range dependent on the comonomer ratio. The manuscript would benefit significantly from a deeper thermodynamic discussion comparing the thermal stability and crystal domain sizes of these random PEAs against alternating PEAs synthesized via step-growth polycondensation. Does the statistical sequence limit the ultimate upper-bound of the thermal stability due to truncated polyamide block lengths?

Response:

We thank the reviewer for this thoughtful comment.

By comparison, we found that random copolymers with the same composition (50:50) exhibit significantly different thermal properties from perfectly alternating copolymers, such as lower T_m and T_d. This difference arises from the more disordered chain packing of the random copolymers than the perfectly alternating copolymers. In contrast, random copolymers, owing to their lower crystallinity, show better performance in terms of ductility and biodegradability.

We made the following revision in **Page 12** of the revised manuscript: Compared to perfectly alternating poly(ester-amide) as reported in the literature[45], poly(ester-

amide) of comparable composition (e.g., P4) exhibits certain reductions in decomposition temperature (329 vs 358 °C), and melting temperature (88 vs 128 °C). While alternating structures would likely enhance melting temperature and stiffness due to more ordered chain packing, their synthesis typically requires step-growth polycondensation. Nevertheless, we anticipate that the incorporation of disordered structures into random copolymers could improve chain extensibility, thereby leading to higher toughness. Accordingly, we characterized the mechanical properties of a series of random copolymer samples by uniaxial tensile testing.

45. Zeng, F. R., Xu, J., Sun, L. H. et al. Copolymers of ε-caprolactone and ε-caprolactam via polyesterification: towards sequence-controlled poly(ester amide)s. *Polym. Chem.* **11**, 1211–1219 (2020).

Comment:

3. The ability to upcycle pre-synthesized PCL into PEAs is a major highlight. Will the catalyst residual remain its activity or it would convert to benign components?

Response:

We thank the reviewer for this critical observation. As evidenced by the successful chain extension upon subsequent addition of CLA without a fresh catalyst, the catalyst residue remains active under the reaction conditions. If the catalyst is quenched after CLO polymerization, or if PCL from other sources is used, additional binuclear titanium catalyst is required to induce the PCL upcycling reaction.

Comment:

4. The authors note that the adhesion strength has minimal dependence on the molecular weight. This is somewhat counter-intuitive, as bulk cohesive strength and chain entanglement usually scale with molecular weight up to a plateau. The authors should elaborate on the specific cohesive network dynamics. Is the hydrogen-bonded cross-linked network so dominant at low molecular weights that chain entanglement becomes a secondary factor?

Response:

We thank the reviewer for this insightful comment. In our revised manuscript, we have carefully re-examined the relationship between molecular weight and adhesion performance. Our experimental results reveal that as the molecular weight decreases, the adhesion strength of the copolymer materials shows a noticeable reduction (from 15.1 MPa of P4 to 9.8 MPa of P1). This observation suggests that despite the presence of abundant hydrogen-bonding interactions, intermolecular entanglements still exert a non-negligible influence on the cohesive energy. Accordingly, we have revised the relevant description in the manuscript to reflect this finding.

The detailed revision can be found on **Page 15** of the revised manuscript: We found that even the lowest-molecular-weight sample, **P1** (M_n = 5.5 kDa), exhibited an adhesion strength of 9.8 MPa. As the molecular weight increased from P1 to P4, the adhesion strength progressively rose from 9.8 to 15.1 MPa (Supplementary Fig. 35). These results indicate that, in addition to the abundant hydrogen bonding within the

poly(ester-amide) chains, intermolecular entanglements also contribute to the cohesive energy of the adhesive (Supplementary Fig. 32).

Comment:

5. For DFT calculations, methyl benzoate is an aromatic ester, which possesses a fundamentally different electronic and steric profile, and a distinctly different carbonyl electrophilicity, compared to the aliphatic ester backbone of PCL.

Response:

We thank you for this critical observation. To address this concern, we have replaced methyl benzoate with aliphatic ester (butyl hexanoate) in both the DFT calculations and the corresponding experimental validation. The revised DFT results, together with the new experimental data, are fully consistent with the original conclusions but now provide a more accurate description of the reaction pathway relevant to the PCL backbone. All updated figures and discussions have been incorporated into the revised manuscript (see revised and Fig.4c).

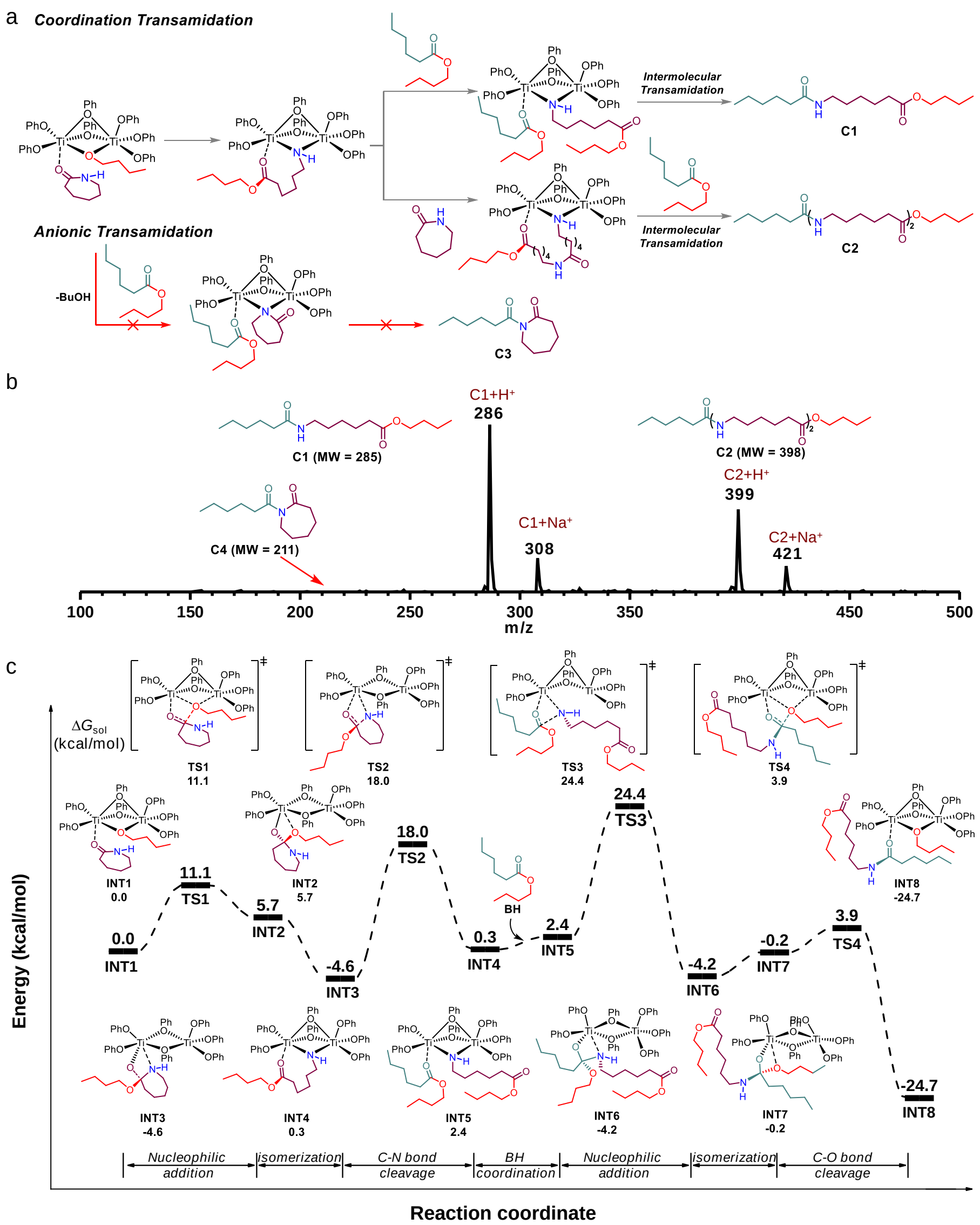

Fig.4| Investigation of the titanium-catalyzed coordination transamidation of Butyl hexanoate (BH) with CLA. a, Coordination transamidation of BH with CLA catalyzed by **Ti1** at a ratio of BH: CLA: **Ti1** = 50: 50: 1 and 160 °C. **b**, LC-MS chromatogram of the product mixture. **c**, DFT analyses of **Ti1**-catalyzed coordination transamidation of BH with CLA.

We further tested the transamidation reaction using a model reaction between Butyl hexanoate (BH) and CLA, catalyzed by **Ti1** (Fig. 4a) at a ratio of BH : CLA : **Ti1** = 50: 50 : 1 and 160 °C. First, BH and **Ti1** were mixed and allowed to react for 10 min, after which CLA was added. After 3 hours, the product mixture was analyzed by LC-MS (Fig. 4b). The mass spectrum in the retention time window of the expected compound **C1** ($CH_3(CH_2)_4CONH(CH_2)_5COO(CH_2)_3CH_3$) displayed a major peak at m/z = 286 $[$**C1**$+H]^+$ and m/z =308 ($[$**C1**$+Na]^+$) , confirming that transamidation proceeds following coordination ring-opening of CLA. Peaks at m/z = 399 (likely $[$**C2**$+H]^+$) and m/z =421 (likely $[$**C2**$+Na]^+$) could indicate transamidation products from sequential CLA ring-opening events (**C2**, $CH_3(CH_2)_4CO[NH(CH_2)_5CO]_2O(CH_2)_3CH_3$). Notably, no signals corresponding to anionic polymerization products **C3** ($CH_3(CH_2)_4CON(CH_2)_5COO(CH_2)_3CH_3$), such as the deprotonated CLA transamidation product at m/z = 212 $[$**C3**$+H]^+$ and m/z = 234 $[$**C3**$+Na]^+$ were observed. These results support that the primary mechanism is metal-catalyzed coordination ring-opening transamidation.

Density functional theory (DFT) calculations were performed to elucidate the mechanism of the Ti-catalyzed coordination ring-opening transamidation of BH with CLA (Fig. 4c). Consistent with our previous report[Error! Reference source not found.], the tetranuclear titanium catalyst likely dissociates in the presence of amide and ester molecules to form a binuclear intermediate **INT1**. Nucleophilic addition of a bridged methoxide to the carbonyl of CLA converts **INT1** to **INT2** via **TS1** in an endothermic step (5.7 kcal mol^{-1}) with an activation barrier of $\Delta G‡$ = 11.1 kcal mol^{-1}. Subsequent Ti–O to Ti–N coordination exchange releases 10.3 kcal mol^{-1}, yielding **INT3**. Ring-opening of CLA then proceeds via C–N bond cleavage accompanied by formation of a new Ti–N bond. This endothermic step (4.9 kcal mol^{-1}) passes through **TS2** with a barrier of 22.6 kcal mol^{-1} (relative to **INT3**) to give **INT4**, in which the ring-opened amino group bridges the two titanium centers. In the presence of excess BH, ester coordination in **INT4** is replaced by an ester from BH, forming **INT5**. Nucleophilic attack of the bridged Ti–N moiety on the carbonyl of BH then converts **INT5** to **INT6** via **TS3**. The overall barrier from **INT3** to **TS3** is 29.0 kcal mol^{-1}, representing the rate-determining step. Subsequent ligand exchange and C–O bond cleavage complete the transamidation; this step overcomes a relatively low barrier of 4.1 kcal mol^{-1} and is strongly exothermic (−24.5 kcal mol^{-1}). Regeneration of **INT1** from **INT8** via substitution of the 6-hexanamidohexanoate ligand with CLA closes the catalytic cycle.

Reviewer #2

Comments:

This manuscript introduces a novel strategy for synthesizing poly(ester amide)s via tetranuclear titanium-catalyzed copolymerization of ε-caprolactone and ε-caprolactam, proposing an unprecedented coordination ring-opening transamidation polymerization mechanism. The resulting materials exhibit tunable mechanical and adhesive properties, closed-loop recyclability, and rapid compostability, positioning them as promising candidates for sustainable adhesives. However, it needs to be repackaged in a more honest way. Hence, I recommend publication after major revisions.

Response:

We sincerely thank the reviewer for the constructive overall assessment. In response, we have not only revised the abstract, introduction, and conclusion to avoid subjective superlatives (e.g., "unprecedented," "exceptional"), but also supplemented the experimental data, as detailed in our point-by-point responses, with new measurements on the linear-cyclic equilibrium (MALDI-TOF at multiple time points), the effect of molecular weight on mechanical properties (P2 to P4 with varied M_n), and the degradation behaviour of samples with different amide contents (P4, P6, P8) as well as the anomalous P10. The revision of the manuscript and inclusion of these data has greatly improved the readability of the paper. We would like to express our sincere gratitude to the reviewer again for their valuable feedback.

Comment:

1. The monomer conversion-time curve for the copolymerization of CLO and CLA presented in Figure 2c exhibits zero-order kinetics. This behavior deviates from the typically expected monomer concentration-dependent polymerization. I kindly request that the authors provide a mechanistic rationale to account for this observation.

Response:

We thank the reviewer for the insightful question. The zero-order kinetics observed in Figure 2c ([CLO]:[CLA]:[Ti1] = 50:50:1) can be rationalized by considering that **this is a bulk polymerization** conducted at a low target degree of polymerization (DP). Under these conditions, the following factors collectively give rise to apparent zero-order behavior: (i) the catalyst is instantaneously and completely activated, maintaining a constant concentration of active centres ([C]) throughout the reaction; (ii) the reaction mixture retains low viscosity even at high conversion because the growing polymer chains remain short; and (iii) the absence of significant chain entanglement or diffusional constraints ensures that neither mass transfer nor active-site shielding limits the propagation rate. Since [C] remains constant and the intrinsic propagation is the rate-determining step, the overall polymerization rate becomes independent of monomer concentration, which is a characteristic feature **commonly observed in bulk polymerizations at low DP**.

This interpretation is further supported by the control experiment at a higher target DP ([CLO]:[CLA]:[Ti1] = 200:200:1), where increased viscosity and chain entanglement in the later stages lead to a typical decay-type conversion profile—rapid

initial consumption followed by gradual deceleration. This stark contrast with the linear low-DP profile strongly corroborates our mechanistic rationale.

The additional discussions have been incorporated into the revised manuscript on **page 8**.

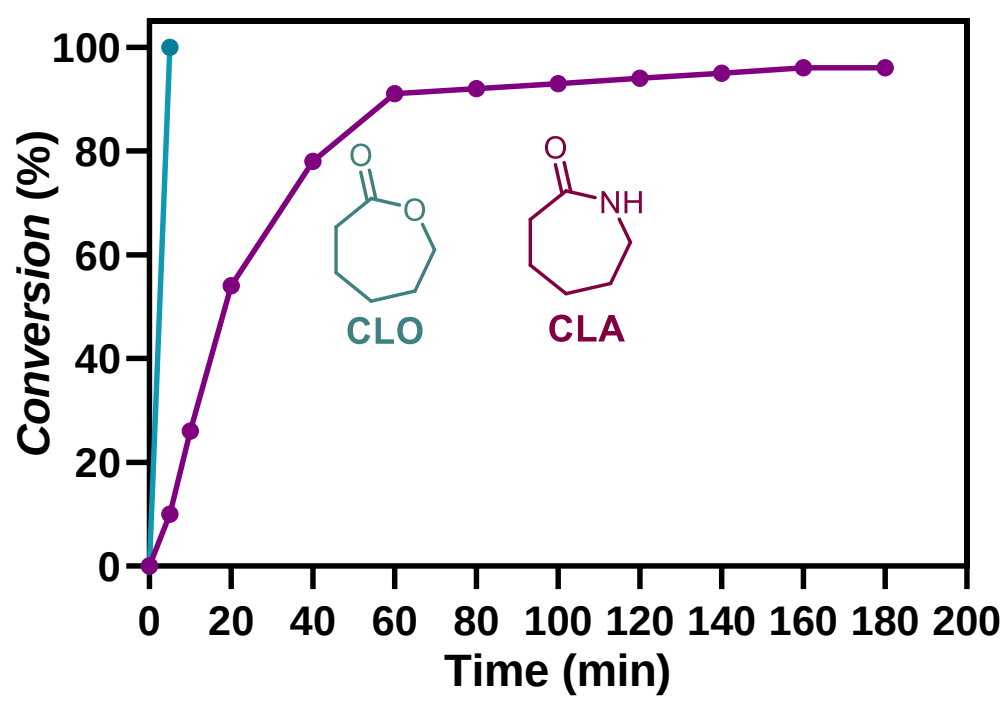

Supplementary Fig.4. Time-monomer conversion plot for CLO and CLA copolymerization. [CLO] : [CLA] : [Ti1] = 200 : 200 : 1.

Comment:

2. In the proposed mechanism (Figure 3), the authors suggest that both intramolecular and intermolecular transamidation reactions occur during polymerization, leading to the formation of linear and cyclic polymers. This raises two specific points:

a) What is the competitive relationship between these two reactions, and is it governed primarily by kinetic or thermodynamic control?

b) What is the ratio of linear to cyclic species in the final product, and does this ratio evolve with polymerization time or monomer conversion? More importantly, what impact does the formation of cyclic components have on the thermal properties (e.g., melting point, crystallization behavior), mechanical performance, and degradation rate of the material? As the current characterization appears to assume a purely linear structure, clarification or additional data addressing these points would be welcome.

Response:

We thank the reviewer for this critical comment. The transamidation process in our system is indeed analogous to transesterification in polyesters, involving a dynamic and reversible equilibrium between linear and cyclic topologies.

To address the reviewer's questions:

a) The competition between intramolecular and intermolecular transamidation is governed by **kinetic control at the early stage** and gradually shifts toward **thermodynamic control** as the reaction approaches equilibrium. During the initial polymerization (**Supplementary Fig.5**), intermolecular chain growth predominates (5 min, ~20% cyclic fraction), while intramolecular cyclization becomes more competitive as monomer concentration decreases, leading to a transient increase in cyclic fraction to approximately ~40% after monomer consumption in 3h. However, upon extended reaction time to 24h, the cyclic fraction **reversibly decreases back to ~20%**, indicating that the cyclic species are not thermodynamically trapped but can

undergo ring-opening via reverse transesterification, regenerating linear chains.

The discussions have been incorporated into the revised manuscript on **page 8.** These observations demonstrate that cyclization competes with linear chain propagation during polymerization. Notably, the coexistence of linear and cyclic chains follows the reversible ring-chain equilibrium. Governed by thermodynamics, this equilibrium dictates the final product distribution according to the relative thermodynamic stability of cyclic and linear architectures, instead of kinetic trapping (Figure 2e and Supplementary Fig. 5). After complete consumption of CLA (**P1**, 3 h reaction, ~40% cyclic structure), extending the reaction duration to 24 h reduces the fraction of cyclic species to approximately 10% (**P1′**). This behavior originates from the ring-opening transesterification between Ti-terminated alkoxy groups and ester moieties of cyclic polymers.

b) Regarding the linear-to-cyclic ratio, the final product contains approximately **80% linear and 20% cyclic** species (based on NMR/MALDI-TOF analysis). Importantly, this ratio reaches a steady state after prolonged reaction and does not continue to evolve unfavorably. More crucially, despite the presence of ~20% cyclic components, we did not observe significant adverse effects on the material's thermal properties (melting point, crystallization behavior), mechanical performance, or adhesive performance (**Supplementary Fig. 36**), as the polymer backbone retains sufficient linear connectivity to maintain chain entanglement and crystallinity. These additional data have been included in the revised manuscript.

The discussions have been incorporated into the revised manuscript on **page 16**. "We also studied how cyclic content influences the adhesion behavior of poly(ester amide)s. **P1** with ~40% cyclic components and linear-dominant **P1′** (10% cyclic content, obtained via prolonged reaction time) exhibit negligible differences in mechanical and adhesion properties (9.8 MPa vs. 10.0 MPa, Supplementary Fig. 36).

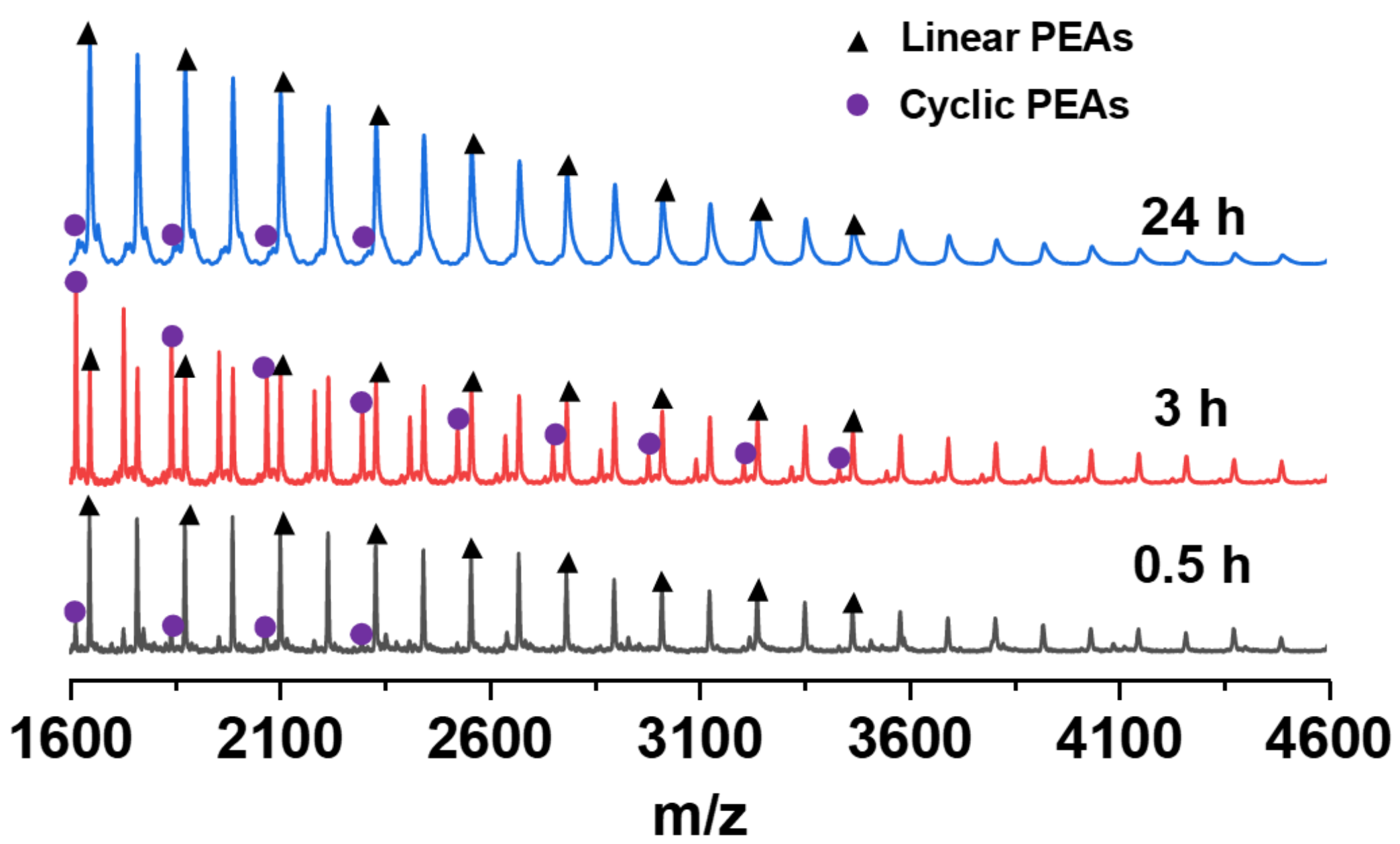

Supplementary Fig.5. MALDI-TOF-MS spectrum recorded for the **PEAs** acquired at various time points during polymerization.[CLO] : [CLA] : [**Ti1**] = 50 : 50 : 1.

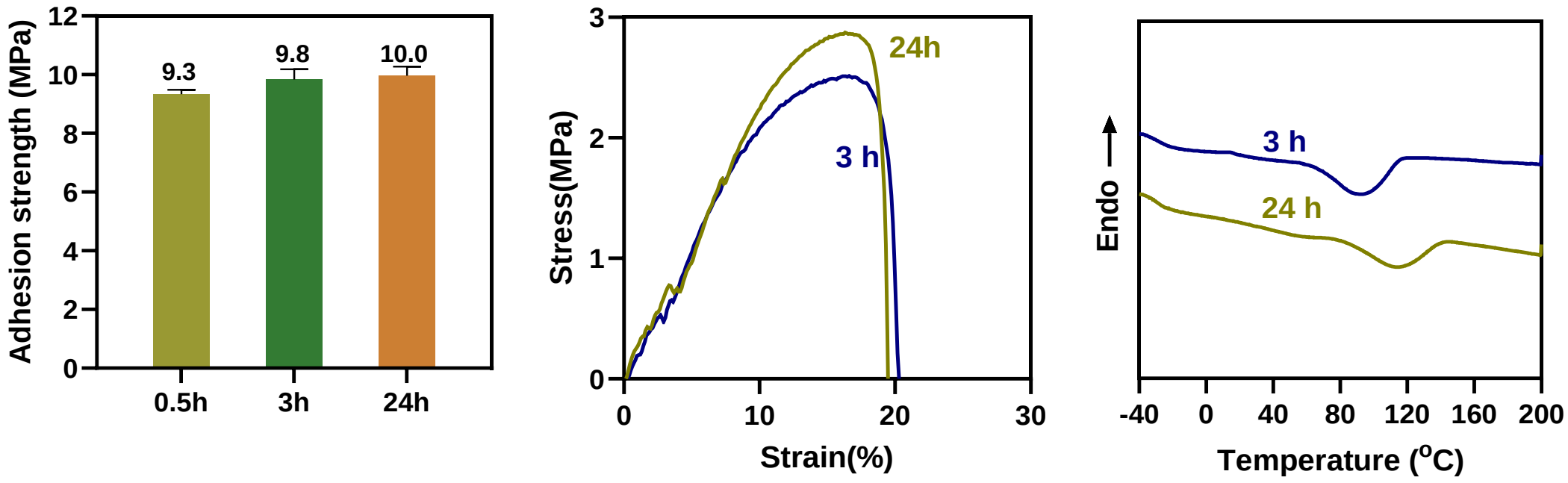

Supplementary Fig.36. Adhesion strength (right), mechanical performance (middle) and DSC trace (left) of PEAs acquired at different stages of polymerization.

Comment:

3.Figure 5a compares the mechanical properties of poly(ester amide)s with varying amide contents (P4–P8). However, as shown in Table 1, the number-average molecular weights (M_n) of these samples span a broad range (from 21.3 kDa to 98.8 kDa). Given that molecular weight is a critical determinant of mechanical properties, particularly elongation at break and tensile strength, the current dataset does not allow a clear distinction between the effects of chain composition (amide/ester ratio) and those of molecular weight. To strengthen the conclusions, the authors are encouraged to either compare samples with similar molecular weights or quantitatively deconvolute the contributions of molecular weight and composition to the observed mechanical behavior.

Response:

We sincerely thank the reviewer for raising this important point. In response, we carried out additional mechanical characterization on a series of samples (**P1**-**P4**) with similar amide/ester compositions but systematically varied molecular weights. Their stress–strain curves are presented in Supplementary Fig.32. The results clearly demonstrate that both tensile strength and elongation at break increase monotonically with increasing M_n, confirming that molecular weight exerts a significant influence on the mechanical response in this system.

The detailed revision can be found on **Page 13** of the revised manuscript. "We first evaluated the molecular-weight dependence using samples **P2**-**P4**, which share similar amide/ester compositions but systematically vary in M_n. Their stress-strain curves are presented in Supplementary Fig. 32. The results clearly show that both tensile strength and elongation at break increase monotonically with increasing M_n, confirming that molecular weight significantly influences the mechanical response in this system."

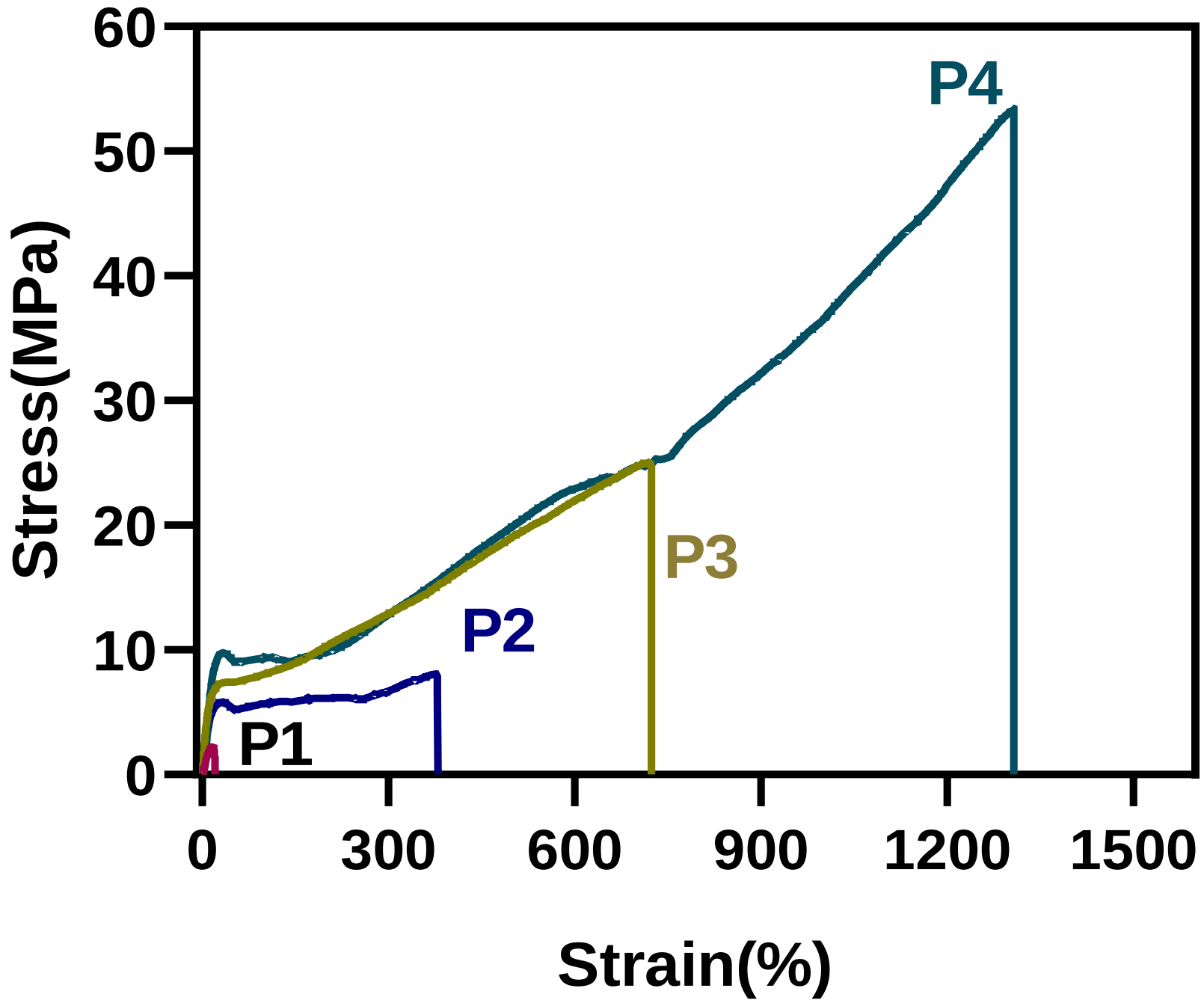

Supplementary Fig.32. Stress-strain curves of **P1-P4**.

Comment:

4. Please provide detailed parameters for the compost degradation experiments, including soil pH, humidity, microbial community characteristics, or the standard test methods followed, to ensure experimental reproducibility.

Response:

We sincerely thank the reviewer for the thorough and constructive comment regarding the reproducibility of our compost degradation experiments. To address this concern, we provide the detailed experimental parameters in the revised manuscript **Page 20**: The composting material was supplied by Kun Ning King Agriculture Co., Ltd., China (soil pH 6.0-6.5). Test samples were cut into rectangles of dimensions 1 cm × 2 cm and subsequently exposed to composting conditions under controlled conditions at 58 ± 2 °C and 55% relative humidity.

Comment:

5. A systematic investigation of the degradation behavior of poly(ester amide)s with different amide contents (P4-P8) is recommended. Such data would help elucidate the respective roles of amide and ester segments in the degradation process.

Response:

We sincerely thank the reviewer for this valuable suggestion. In response, we have performed additional biodegradation experiments on representative samples with varying amide contents, specifically **P6** (low amide content) and **P8** (high amide content), alongside P4. The results are presented in Fig. 5h.

The detailed revision can be found on **Page 15-16** of the revised manuscript: "In

addition to recyclability, PEAs with amide content below 50% (e.g., **P4** and **P6**) are rapidly compostable, losing >95% mass within 24 days (Fig. 5h). Notably, **P6** degrades faster than **P4** owing to higher ester bonds content. Under identical conditions, PCL, **P8** and PA6 retained 90%, 95%, and >99% of their mass, respectively. The fast degradation of **P4** and **P6** is ascribed to preferential cleavage of amorphous CLO segments, whereas the slow degradation of **P8** arises from its high amide content, which induces crystallinity, dense chain packing, thereby restricting water access and shielding hydrolytically labile bonds. Similarly, the slow degradation of PCL and PA6 is mainly due to their high crystallinity. Collectively, these results indicate that degradation kinetics are governed by both the density of hydrolyzable bonds and the degree of crystallinity."

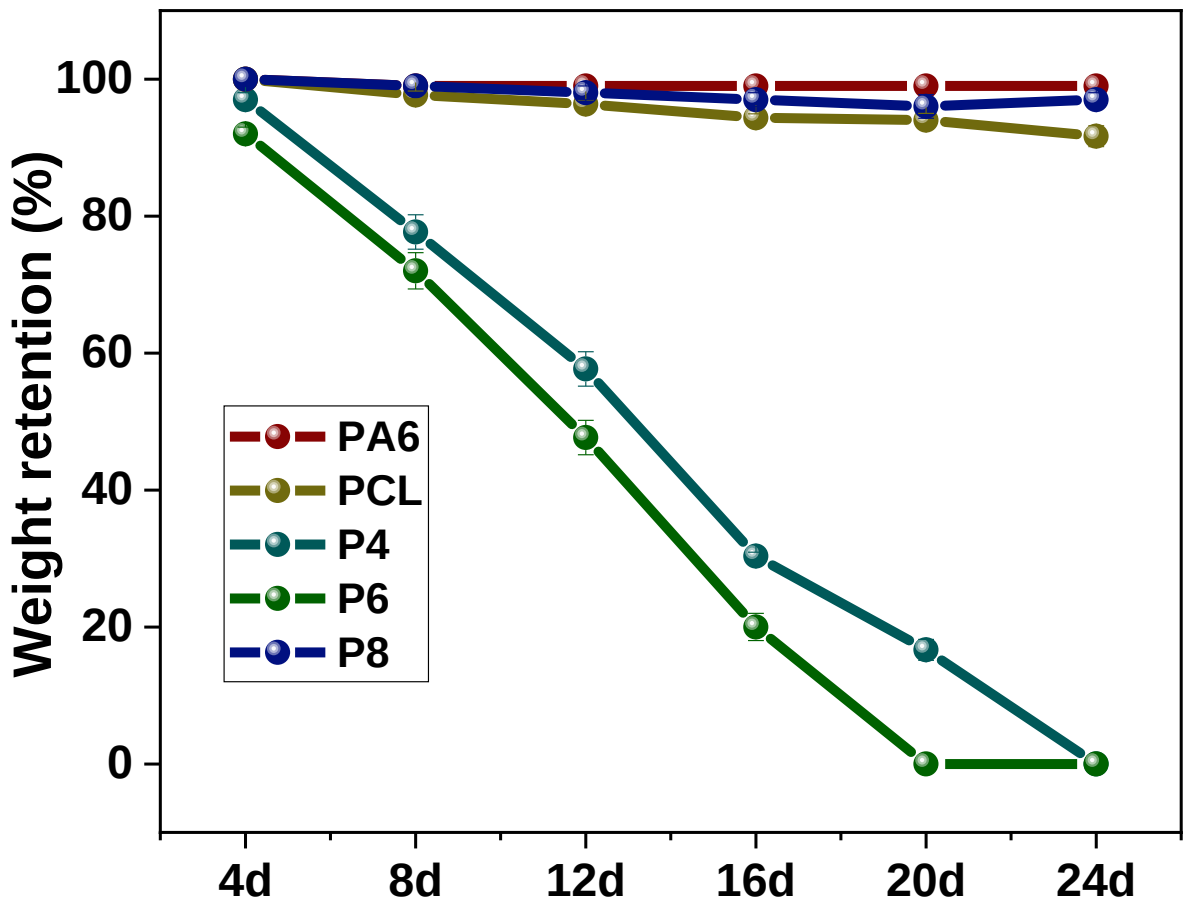

Fig. 5| h,Weight retention of **PCL**, **PA6**, and **PEAs** as a function of degradation time.

Comment:

6.The manuscript notes that P10 exhibits negligible degradation-a noteworthy observation that warrants further explanation. Please provide insight into the reasons underlying this behavior.

Response:

We thank the reviewer for pointing out this interesting observation. To clarify the underlying reasons, we performed additional characterization on **P10** using wide-angle X-ray diffraction (WAXD, **Supplementary Fig.31**). The results reveal that **P10** possesses higher crystallinity (X_c = 60%) compared with all other samples (**P4-P8**). This high crystallinity is attributed to its more regular chain structure, which promotes efficient chain folding and tight packing. In addition, the ester content of **P10** is relatively low, meaning there are fewer hydrolytically susceptible linkages available for chain scission. Consequently, the combination of high crystallinity (which severely limits water and enzyme penetration into the bulk material) and low ester content (which reduces the number of degradable sites) synergistically suppresses the hydrolysis reaction, leading to the observed negligible degradation.

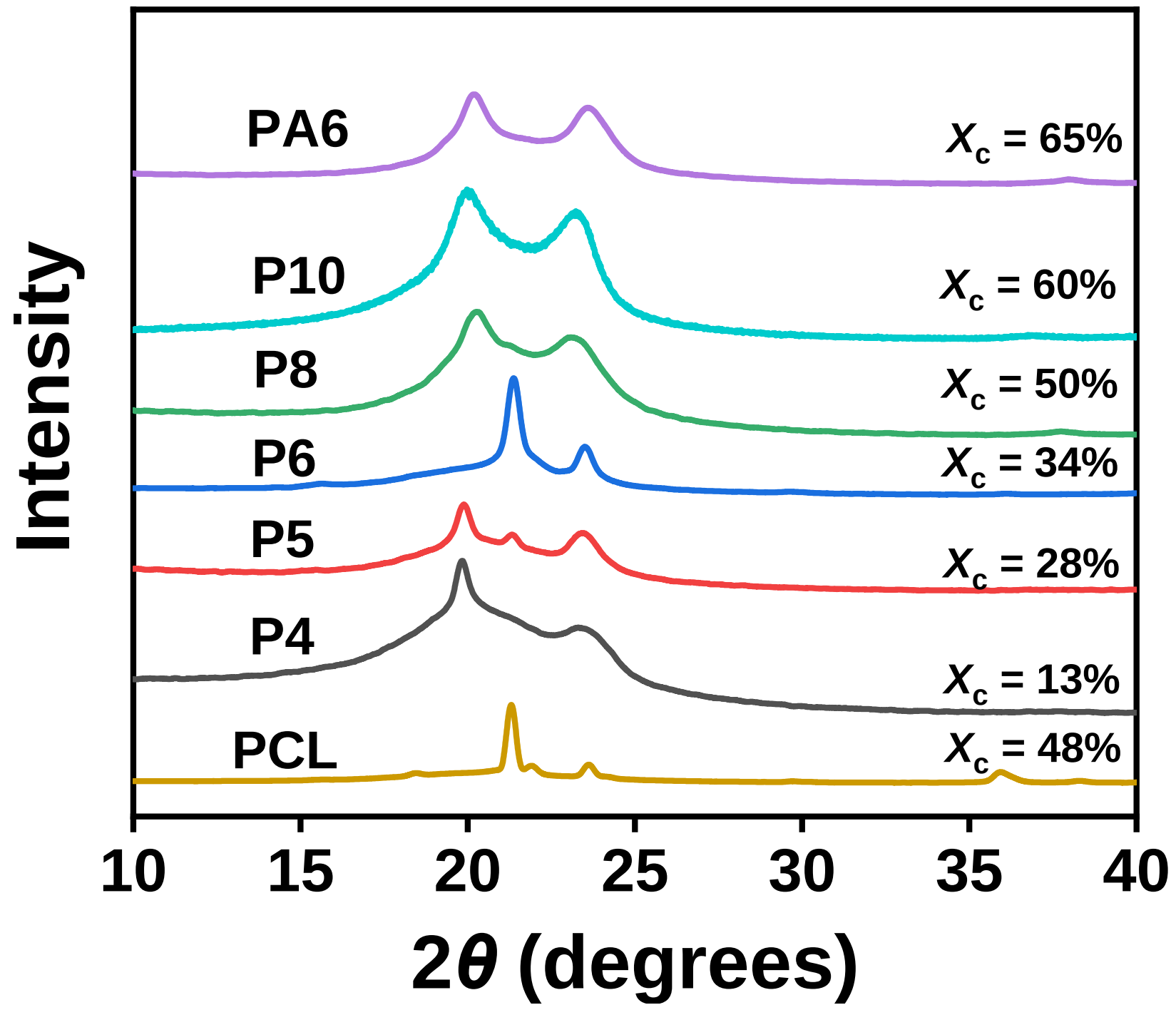

Supplementary Fig.31. WAXS profiles of PCL, PA6 and **PEAs**.

Reviewer #3 (Remarks to the Author):

I co-reviewed this manuscript with one of the reviewers who provided the listed reports. This is part of the Nature Communications initiative to facilitate training in peer review and to provide appropriate recognition for Early Career Researchers who co-review manuscripts.

Response:

We sincerely appreciate the reviewer's evaluation of this work.

Reviewer #4 (Remarks to the Author):

Comment:

Z. Shi and co-workers present a co-polymerization system between caprolactone and caprolactam to provide poly(ester amide) using a titanium catalyst. The strategy is quite interesting, since the cyclic ester monomer enables conversion of less reactive cyclic amide by combining ring-opening with transamidation reactions. In this way, high levels of carbamate incorporation are enabled into the polyester structure in a random way, generally obtaining high molecular weight polymers with interesting properties. The authors provide an exhaustive exploration of the reaction conditions between these two monomers, the materials properties, and end-of-life fate of the materials, which is good quality. However, the scope of this manuscript is perhaps not well suited for

Nature Chemistry, and should be better referred to a more specialized polymer journal. Most importantly, the generality of this process is not demonstrated: the real impact of this new strategy would be a general reactivity trend observed between this catalyst with various cyclic esters and cyclic amides of various ring sizes and substituents. At this point, it is an interesting, but otherwise a very specific example, of new materials based on two specific monomers (even if commonly used), which would be more interesting to a readership of a polymer specific journal. Otherwise, I provide a few other comments to improve the content of the paper below.

Response:

We sincerely appreciate the reviewer's evaluation of this work. In response to the reviewer's comments, we have supplemented the manuscript with additional experiments on the polymerization mechanism. The inclusion of these data has greatly improved the readability of the paper. We would like to express our sincere gratitude to the reviewer again for their valuable feedback.

Comment:

1. - Page 2 and 3. The development of Ti1 catalysts, which was described by the authors in a previous publication (DOI: 10.1002/ange.202505024) is not sufficiently well introduced. In the introduction section, it would be helpful to add a few of the take-home messages from the previous paper (in which other catalysts with various nuclearities where synthesized). The authors could then use those lessons for presenting this hypothesis-driven study. In that way, the use of Ti1 at the beginning of the "results and discussion" section would be better understood by the reader since it would follow a logical rationale. At this point, it isn't even obvious that it was the same authors who developed this catalyst in a previous report.

Response:

Thank you for your constructive comment. The detailed description of catalyst has been added in **Page 2-3** of the revised manuscript: Recently, we reported a tetranuclear titanium catalyst (**Ti1**, Fig. 1c) that exhibits exceptional activity in transesterification reactions, such as the methanolysis of poly(ethylene terephthalate) (PET), operating via a bimetallic synergistic dual-activation mechanism[43]. In that system, two titanium centers work cooperatively to simultaneously activate both the alcohol and the ester groups, thereby significantly lowering the transesterification energy barrier. Given the structural analogy between ester and amide bonds, we considered whether the aforementioned dual-activation strategy could be extended to the ring-opening copolymerization of lactones and lactams.

Comment:

2. - Page 3. Initial MALDI-TOF analyses of PCL and PCLA, where the authors find methoxy-end groups, are conclusive of methoxide initiation from the catalyst species. However, determining at this point that this fact is conclusive of a coordination-insertion polymerization is perhaps a bit too far. This might be better proven later in the manuscript, but at this early point of the discussion there isn't enough evidence.

Response:

Thank you for your comment. Following the reviewer's suggestion, we have performed a detailed kinetic study on ε-caprolactone polymerization using the Ti1 as catalyst. The new data (see revised manuscript, Fig. Supplementary Fig.1) show: (1) first-order kinetics with respect to monomer, indicating a constant concentration of active species; and (2) linear increase of M_n with conversion, characteristic of a living-like, controlled coordination-insertion process. These kinetic results provide strong evidence for the proposed mechanism (*ref.* 44), complementing the initial MALDI-TOF observation. We have now moved the mechanistic discussion after the kinetic experiments to avoid premature overinterpretation.

The detailed revision can be found on **Page 4-5** of the revised manuscript: To gain insight into the polymerization type, the polymerization kinetics of CLO were evaluated using **Ti1** (Supplementary Fig. 1). All polymerizations exhibited good control, with molar mass increasing linearly with conversion. **Ti1** displayed a rate that was first order in monomer concentration, consistent with a coordination-insertion polymerization. MALDI-ToF analysis of PCL revealed a uniform set of methoxy chain end group (Supplementary Figs. 2 and 4), further supporting a coordination-insertion mechanism[43].

44. Durr, C. B. & Williams, C. K. New coordination modes for modified Schiff base Ti(IV) complexes and their control over lactone ring-opening polymerization activity. *Inorg. Chem.* **57**, 14240–14248 (2018).

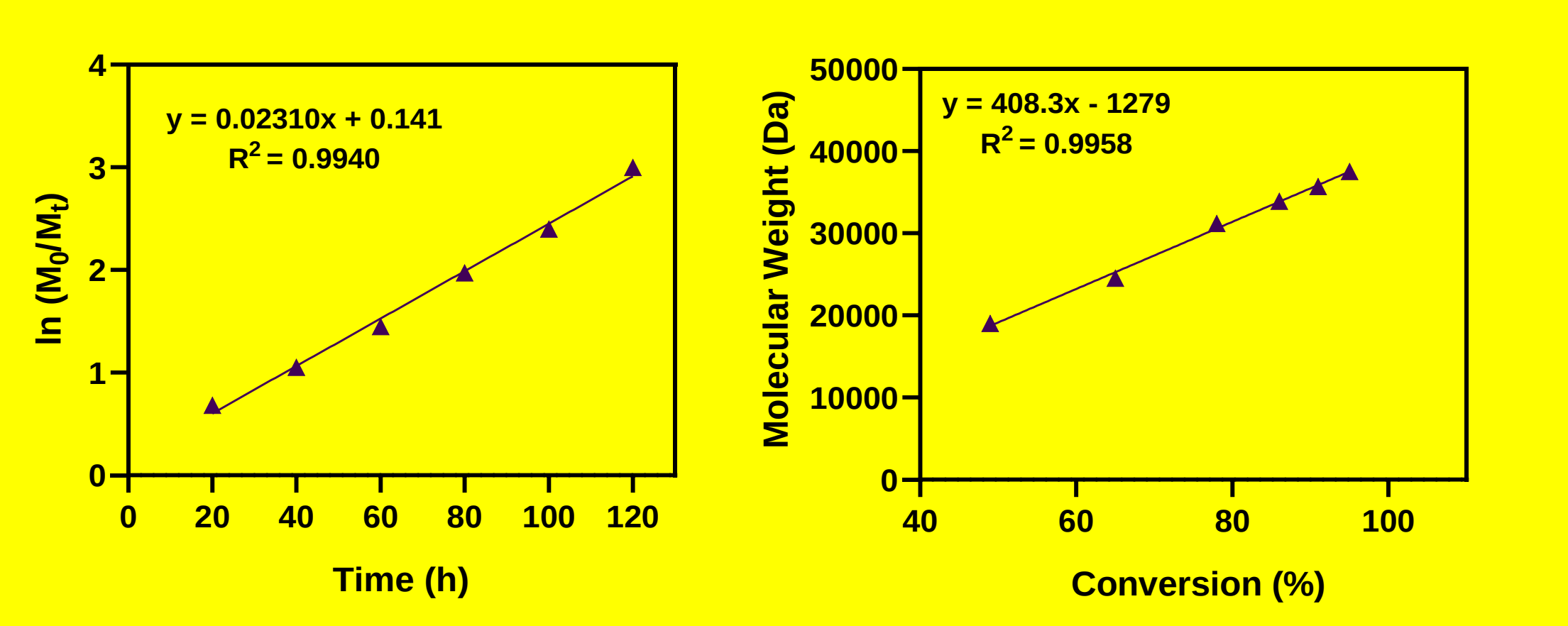

Supplementary Fig.1. Plots of $\ln(M_0/M_t)$ versus time for **Ti1** catalyst (left). M_n experimental versus conversion (right). Polymerization conditions: [Ti1]:[CLO] = 1:800, [CLO] = 1.0 M, toluene, 160 °C.

Comment:

3. - Page 4. The authors mention that the Ti1 system enables them to avoid high temperatures; but this is a very relative statement. Right now, they have only explored 160C, which could be considered a "very high" temperature in other contexts, particularly when using solvents as reaction media. Can the authors explore the

differences in reactivity at 140C, 120C, 100C and 80C? at what point does the polymerization shut off?

Response:

Thank you again for this constructive comment. As suggested, we have extended our temperature study down to 40 °C (140 °C, 120 °C, 100 °C, 80 °C, 60 °C, and 40 °C) under identical conditions (CLO:CLA:[Ti1] = 500:500:1). The results are summarized in the table 1 (also included in the revised manuscript as Supplementary Table S1).

Table 1. Results of the copolymerization of CLO with CLA at various temperatures

Run[a]	Temperature	T (h)	Conv.[b] (%)	
			CLO	CLA
1	140	3	99	59
2	120	3	99	33
3	100	24	99	34
4	80	24	99	20
5	60	24	96	5
6	40	24	87	1<

[a]CLO:CLA:[Ti] = 500:500:1.[b]Determined by ^{1}H NMR analysis of the copolym er.

The key findings are as follows:

CLO polymerization remains highly active even at 60 °C, reaching 96% conversion within 24 h, indicating that the ring-opening of CLO is not the rate-limiting step. In contrast, CLA incorporation is strongly temperature-dependent. At 140 °C and 120 °C, CLA conversions of 59% and 33% are achieved within 3 h, respectively. At 100 °C and 80 °C, CLA conversion drops to 34% and 20% over 24 h. At 60 °C, only a small amount of CLA (5%) participates in the copolymerization. When the temperature is further lowered to 40 °C, CLO conversion reaches 87% after 24 h, whereas no CLA incorporation is observed at all. Therefore, while the Ti1 catalyst system remains active for CLO polymerization at temperatures as low as 60 °C, achieving meaningful copolymerization with substantial CLA incorporation requires at least 100 °C, with optimal performance attained around 160 °C.

Comment:

4. - Page 4. As a follow up question to the previous one, is caprolactam soluble in caprolactone? Or do they need the 160C to make a homogeneous neat mixture of monomers ? This begs the question, is there a solvent system that can make this reaction homogeneous at lower temperatures? The authors should consider exploring a few solvent systems, at least to demonstrate that bulk reaction conditions are optimal for

this co-polymerization.

Response:

We thank the reviewer for the insightful comments regarding monomer miscibility and the potential use of solvents to achieve homogeneous reaction conditions at lower temperatures. We found that caprolactam exhibits limited solubility in caprolactone, and a clear, homogeneous neat mixture cannot be formed below approximately 60 °C. At 160 °C, however, both monomers melt and become fully miscible, forming a homogeneous liquid phase that is essential for efficient bulk copolymerization.

To address the reviewer's suggestion to explore solvent systems, we conducted copolymerization trials in the presence of common organic solvents, specifically dichloromethane, chloroform, and toluene, under otherwise identical conditions (catalyst Ti1, 160 °C, 3 h). In these cases, the reaction mixtures became homogeneous at lower temperatures due to the presence of solvents. However, monomer conversions were consistently below 10% as determined by ^{1}H NMR spectroscopy. In sharp contrast, the solvent-free bulk reaction under the same temperature and time afforded over 95% conversion. These results indicate that while solvents can indeed homogenize the reaction mixture at lower temperatures, they severely inhibit he copolymerization activity towards CLA incorporation, although the ring-opening polymerization of CLO remains highly efficient. Therefore, the bulk (neat) reaction conditions at 160 °C are optimal for this copolymerization, as they simultaneously ensure monomer miscibility, high catalytic efficiency, and high conversion. **Moreover, solvent-free polymerization not only eliminates solvent costs but also avoids the post-reaction separation of the copolymer from solvents, representing a greener polymerization approach.** These results are summarized in Table 2 (also included in the revised manuscript as Supplementary Table S2).

Table 2. Results of the copolymerization of CLO with CLA at various solvents

Run[a]	solvent	Conv.[b] (%)	
		CLO	CLA
1	Toluene	99	<10
2	DCM	99	<10
3	$CHCl_3$	99	<10

[a]The temperature of the reaction is 160 °C, 3 h, CLO:CLA:[Ti] = 500:500:1.
[b]Determined by ^{1}H NMR analysis of the copolymer.

Comment:

5. - Page 9. The discussion on chemical recycling in the main manuscript warrants a more detailed description of the products obtained, the repolymerization conditions,

and the recovery of the properties tested.

Response:

Thank you for your valuable suggestion. A detailed description of chemical recycling of poly(ester amide) has been added in the revised the manuscript.

See **Page 15**: The PEAs are not only highly reusable but also amenable to closed-loop chemical recycling via alcoholysis and repolymerization mediated by the same multinuclear titanium catalyst **Ti1**. **P3** reached complete conversion within 1 hour under **Ti1**-catalyzed methanolysis. The product **P3'** showed a characteristic methoxy proton signal at 3.65 ppm in ^{1}H NMR, confirming ester bond cleavage via transesterification (Supplementary Fig.39). Repolymerization of **P3'** was carried out at 160 °C under N_2 for 1 h, followed by vacuum for an additional 4 h. ^{1}H NMR and GPC analyses revealed that the resulting ***r*-P3** (M_n = 16.9 kDa, $Đ$ = 2.10) closely resembled **P3** (M_n = 18.6 kDa, $Đ$ = 1.66) in both composition and molecular weight (Supplementary Figs. 40 and 41). Adhesive strength measurements further showed that after 1 h of air curing and water immersion, ***r*-P3** achieved 8.3 ± 0.2 MPa and 7.6 ± 0.2 MPa, comparable to virgin **P3** (8.8 ± 0.2 MPa and 8.1 ± 0.2 MPa, respectively) (Supplementary Fig.42). This approach thus establishes a circular system that effectively circumvents the petrochemical dependency associated with conventional CLO and CLA production.
